# Justice Evaluation of the Income Distribution (JEID): Development and validation of a short scale for the subjective assessment of objective differences in earnings

**DOI:** 10.1371/journal.pone.0281021

**Published:** 2023-01-26

**Authors:** Désirée Nießen, Jule Adriaans, Stefan Liebig, Clemens M. Lechner

**Affiliations:** 1 GESIS–Leibniz Institute for the Social Sciences, Mannheim, Germany; 2 Faculty of Sociology, Bielefeld University, Bielefeld, Germany; 3 Institute of Sociology, Freie Universität Berlin, Berlin, Germany; Polytechnic Institute of Coimbra: Instituto Politecnico de Coimbra, PORTUGAL

## Abstract

Justice evaluations are proposed to provide a link between the objective level of inequality and the consequences at the individual and societal level. Available instruments, however, focus on the subjective perception of inequality and income distributions. In light of findings that subjective perceptions of inequality and income levels can be biased and subject to method effects, we present the newly developed Justice Evaluation of the Income Distribution (JEID) Scale, which captures justice evaluations of the actual earnings distribution. JEID comprises five items that provide respondents with earnings information for five groups at different segments along the distribution of earnings in a given country. We provide a German-language and an English-language version of the scale. The German-language version was developed and validated based on three comprehensive heterogeneous quota samples from Germany; the translated English-language version was validated in one comprehensive heterogeneous quota sample from the UK. Using latent profile analysis and *k*-means clustering, we identified three typical response patterns, which we labeled “inequality averse,” “bottom-inequality averse,” and “status quo justification.” JEID was found to be related to normative orientations in the sense that egalitarian views were associated with stronger injustice evaluations at the bottom and top ends of the earnings distribution. With a completion time of between 1.50 and 2.75 min, the JEID scale can be applied in any self-report survey in the social sciences to investigate the distribution, precursors, and consequences of individuals’ subjective evaluations of objective differences in earnings.

## Introduction

While global inequality shows a declining trend [1], considerable inequalities in income within and between countries persist worldwide [2]. A substantial literature aims to assess the extent, dynamics, and consequences of these objective inequalities (for an overview, see [3]). However, another branch of literature focuses on the determinants and consequences of subjective evaluations of these inequalities. Justice theory proposes that this subjective perspective on inequality is crucial because the consequences of economic inequality for behavioral and attitudinal outcomes depend on subjective evaluations of existing inequality as just vs. unjust, as legitimate vs. illegitimate, or as fair vs. unfair [4–7]. Indeed, research has shown that subjective evaluations of economic inequality are related to a wide scope of consequences, ranging from well-being [8–11] to political attitudes [12–14].

Despite the scholars of inequality and justice’s inherent interest in the link between objective inequalities, their evaluation, and their consequences, appropriate measures to assess subjective evaluations of *objective* inequalities are in short supply. Previous attempts to measure subjective evaluations of income inequality have highlighted the level of inequality that respondents perceived [4–6]. We argue that by failing to provide respondents with information on actual differences in income, these evaluations lack a link to the actual income distribution that would allow statements to be made about what differences in income individuals tolerate or perceive as just. This is especially relevant because previous research documents that individuals, on average, misperceive economic inequalities [15–19].

To address this gap in existing measurement instruments, we developed the Justice Evaluation of the Income Distribution (JEID) Scale, which links respondents’ subjective evaluations of earnings directly to the objective level of earnings differences within a given country. It does so by asking respondents to evaluate the earnings of five groups—exemplified by typical occupations—that represent five different segments of the earnings distribution. Furthermore, by eliciting evaluations across the earnings distribution, the JEID scale allows researchers to identify critical segments of the distribution of earnings at which people perceive a *justice deficit/gap* [20]. This is important because empirical evidence suggests that people do not consider income inequality to be problematic in itself [21]. Rather, they criticize extreme levels of social inequality [22]. In the following, we describe the theoretical background and the development of the JEID scale.

## Theoretical background

### Justice evaluations

Earnings inequality is the result of a distribution process. In this sense, the evaluation of earnings inequality falls into the domain of distributive justice [23]. More specifically, and staying within the terminology of empirical justice research, it is an *outcome-related* justice evaluation [7]—that is, it refers to individuals’ sense of justice with regard to the outcomes of a distributive process (i.e., how just are the rewards they or others receive [20, 23]). Following Janmaat’s [24] classification of subjective views on inequality, the JEID scale aims to capture “normative evaluations of existing inequality (*i*.*e*. thoughts about how desirable or good the current situation is)” (p. 359).

The empirical investigation of such outcome-related justice evaluations is rooted in the *justice evaluation function* [23, 25]. According to this line of research, people evaluate rewards (e.g., earnings) by comparing the reward that is actually received (denoted as A) with the reward that is perceived to be just (denoted as C). The assumption is that the justice evaluation of a reward (J) can be expressed as the natural logarithm of the ratio of the actual reward (A) to the reward that is seen as just (C): J = ln(A/C). Following Jasso [25], justice evaluations can be depicted as a continuous variable: Zero represents perfect justice, whereas positive values represent unjust overreward, and negative values represent unjust underreward. The justice evaluation function applies to the evaluation of one’s own rewards (*reflexive* justice evaluation) as well as to the evaluation of the rewards of others (*non-reflexive* justice evaluation) and the corresponding distribution [26]. The JEID scale falls into the latter category of non-reflexive justice evaluation. The *justice evaluation function* emphasizes that both the actual reward and the reward that is considered just are crucial in shaping justice evaluations. It thus provides the theoretical link between subjective perceptions and the actual distribution that has been missing in previous attempts to assess perceptions of justice in terms of the actual extent of differences in earnings within a country.

Individuals differ in what they perceive to be a just reward, and these perceptions are shaped by a number of factors related both to individual characteristics of the rewardees and to observers’ ideas of what the overall distribution of rewards should look like [27]. *Equity theory* [28–29] points to the importance of considering inputs that are exchanged for (monetary) rewards. For example, observers of justice expect higher just rewards in return for increased investments, for example, in education or experience. Equity is often described as the dominant principle of distributive justice in the sphere of income and work [30–31]. However, three further fundamental principles of distributive justice have been identified by justice theorists: (a) need, according to which goods and burdens should be allocated based on individual needs; (b) equality, according to which goods and burdens should be distributed equally; and (c) entitlement, according to which status should be the key determinant of just distributions [30, 32].

### Limitations of existing measures

The subjective evaluation of income from work and income inequality more generally has received wide attention in previous research, but the available instruments focused on measuring the evaluation of subjectively perceived levels of inequality [4–6] and thus failed to provide a link from actual inequalities to the justice evaluations of these inequalities. A first, very basic approach used in past research asks respondents to indicate whether they think that income differences in their country are too large. As this single item combines “the perception of income inequality and the respondents’ opinion about the fairness” (p. 137) [6], it lacks comparability across individuals and assesses subjective justice evaluations of subjectively perceived income differences. Moreover, the item asks only for a broad evaluation of income differences, such that respondents and researchers do not know where inequalities occur in the income distribution.

The latter issue is addressed by a second approach, where respondents are asked to evaluate visual representations of hypothetical income distributions. These evaluations are then compared with the actual income distribution. In the ISSP, for example, respondents are presented with five diagrams showing ideal-typical social systems and corresponding descriptions. They are then asked to choose the diagram that, in their view, best describes the current situation in their country and to indicate which distribution they would prefer [33]. Whereas this approach allows for evaluations to distinguish what kind of income distribution respondents perceive and prefer, they refer to stylized ideal types.

A third approach is one that has been used in several cross-national survey programs, such as the International Social Justice Project (ISJP [34]) and the International Social Survey Programme (ISSP [33]), and offers a more fine-grained analysis of perceived earnings inequality compared to the stylized income distributions. Respondents are asked, first, to estimate the actual earnings of people in example occupations representing different levels of the earnings distribution, and, second, to indicate what they think people in those jobs ought to be paid [20]. The relationship between the perceived actual and perceived just earnings of chief executive officers (CEOs) and unskilled workers is then used as a measure of perceived actual and desired inequality [35–37]. However, this approach suffers from focusing on a small set of very specific occupations and therefore does not provide a measure of the justice of the income distribution as a whole. Moreover, extreme earnings values reported in open questions on actual and just earnings may bias results [38].

All three approaches confound two separate phenomena: respondents’ *perceptions* of income distributions (and the corresponding level of income inequality) and their *evaluations* of that income distribution (and the corresponding income inequality). This introduces imprecision into the link between empirical assessment and the underlying construct. This imprecision is exemplified by the fact that previous research has found that survey respondents misperceive inequality [15–19]. Survey respondents in Germany and other European countries tend to assume that there are more people at the lower income levels in society than is the case in the actual income distribution [19]. Therefore, it is not surprising that, “when perceived [as opposed to actual] income inequality is low, fewer people will consider the differences in incomes ‘too large’” (p. 2) [39]. This bias in the assessment of income distribution is critical because empirical evidence suggests that people tend to evaluate in particular the extremes of the income distribution—for example, poverty and extreme wealth—as unjust [22]. Moreover, a recent study comparing estimates of perceived (and preferred) inequality across different measures revealed strong method effects [38]. To better understand the link between objective income inequality—as substantiated in the earnings distribution—and its theorized consequences, a measurement instrument that assesses subjective evaluations of the justice of the actual earnings distribution and that covers a wide range of the income distribution is therefore crucial. The JEID scale was developed to address this need.

## Properties of the JEID scale and aims of the present studies

The JEID scale was designed to measure the construct *subjective evaluation of the objective earnings distribution*. In order to (a) link justice evaluations of the earnings distribution to the actual distribution of earnings and (b) make evaluations comparable across individuals, the scale provides contextual information on average gross earnings and typical occupations. Respondents are asked whether they find low, middle, upper-middle, high, and top incomes unfairly low, fair, or unfairly high. This operationalization is characterized by two crucial choices: First, it acknowledges that respondents in general population surveys (i.e., laypersons) struggle to think in terms of distributions and attempts to capture complex perceptions of inequality are subject to substantive method effects [38, 40, 41]. Instead, we argue that it is more intuitive for respondents to focus on specific points in a distribution (e.g., what the earnings of the worst-off look like). Accordingly, respondents are presented with information about the earnings distribution in the form of specific income groups with accompanying information of where in the distribution a specific income group is placed (e.g., what is the share of people who earn more or less than them). Second, the proposed JEID scale focuses on income from (dependent) work. We opted for this narrow income concept because earnings are the main source of household income both in the UK and in Germany and capture an important element of economic inequality. We discuss limitations of these two design choices in the concluding section of the paper.

This approach of providing context information on actual earnings and occupations when assessing respondents’ sense of justice was first introduced in the employee survey Legitimation of Inequality over the Life-Span (LINOS [42]) and as part of the European Social Survey (ESS) Round 9 module “Justice and Fairness in Europe” [43]. However, these first empirical forays were limited to isolated evaluations of earnings at the very bottom and the very top of the earnings distribution. Furthermore, in the case of the ESS, example occupations were not included, and respondents were thus provided with less context information. Providing example occupations is, however, highly relevant because individuals differentiate between legitimate and illegitimate inequalities, and occupations carry information on ability, training, and status, all of which are central in determining justice evaluations of earnings [23, 44].

With the JEID scale, we developed this novel approach further to allow respondents to state their personal sense of justice based on the same objective facts, thus ensuring the comparability across individuals of the perceptions of the earnings distribution that underlie these justice evaluations. By extending measurement to a broad earnings spectrum, it is possible to identify the critical segments of the earnings distribution at which justice deficits/gaps [20] are perceived. Additionally, the JEID scale is based on the *justice evaluation function*, which is widely used in the empirical justice literature. As analogous measurements of reflexive justice evaluations are available, the JEID scale allows the comparison/contrast of the evaluation of the earnings across the income distribution (i.e., the rewards of others) with the evaluation of own rewards.

The aim of the present studies was threefold: (a) to develop and validate the German-language JEID scale based on three quota samples representing the heterogeneity of the adult population in Germany in terms of age, sex, and educational attainment; (b) to adapt the validated German-language scale to the English language; and (c) to validate the English-language version of the JEID scale and to compare the scale properties of both language versions.

## Method

### Samples

To develop the JEID scale and investigate its psychometric properties, we assessed JEID in three Web-based surveys (using computer-assisted self-administered interviewing [CASI]) that we conducted via the online access panel provider responding AG. Data collection took place in Germany in January/February 2020 (Study 1) and in August 2020 (Study 2), and parallel in Germany and the UK in July 2021 (Study 3). We drew quota samples that represented the heterogeneity of the adult population in terms of age, sex, and educational attainment. Data from the last German Census (2011) were used as a reference for Studies 1 and 2 (https://ergebnisse.zensus2011.de/?locale=en). Data from the German Microcensus from 2018 were used as a reference for Study 3 (https://www-genesis.destatis.de/genesis/online?locale=en). To avoid bias introduced by a lack of reading/language proficiency, only native speakers were recruited. We explained our research goal (investigation of the quality of several questionnaires) to the participants. Respondents consented to their participation in an anonymous online survey and received a small financial incentive for their participation; no sensitive material was collected; and no special ethical review and approval was required under German law. We adhered to ethical standards comparable to the 1964 Declaration of Helsinki.

To assess test–retest reliability, we invited a subsample in all three studies to participate in a follow-up survey 2 weeks after the median day of the main survey. Only respondents who completed the full questionnaire (i.e., respondents who did not abort the survey prematurely) were included in the analyses. The gross sample sizes were *N*_Study 1_ = 531, *N*_Study 2_ = 618, *N*_Study 3, Germany_ = 463, and *N*_Study 3, UK_ = 483. After excluding invalid cases, the net sample sizes were *N*_Study 1_ = 486 (retest: *N*_Study 1_ = 189), *N*_Study 2_ = 618 (retest: *N*_Study 2_ = 299), and *N*_Study 3, Germany_ = 420 (retest: *N*_Study 3, Germany_ = 202), and *N*_Study 3, UK_ = 440 (retest: *N*_Study 3, UK_ = 199). Table 1 shows in detail the sample characteristics and their distribution.

**Table 1:** Sample characteristics by study.

	Study 1	Study 2	Study 3	
			Germany	UK
*N* (retest)	486 (189)	618 (299)	420 (202)	440 (199)
Mean age in years (*SD*) [range]	44.3 (14.8) [18–69]	43.4 (14.9) [18–69]	43.6 (14.0) [18–65]	44.4 (13.1) [19–65]
Proportion of women in % (*n*)	51.2 (249)	49.5 (306)	51.2 (215)	49.8 (219)
Educational attainment in % (*n*)				
Low	34.3 (167)	36.1 (223)	26.0 (109)	24.3 (107)
Intermediate	34.0 (165)	32.8 (203)	33.8 (142)	33.0 (145)
High	31.7 (154)	31.1 (192)	40.2 (169)	42.7 (188)

*Note*. The German educational attainment levels were as follows: low = no educational qualification/basic school-leaving qualification (*ohne Bildungsabschluss/Hauptschulabschluss*); intermediate = intermediate school-leaving qualification (*Mittlere Reife/Realschulabschluss*); high = entrance qualification for a university of applied sciences/general higher education entrance qualification (*Fachhochschulreife/Abitur*). The UK educational attainment levels were as follows: low = never went to school, Skills for Life/1–4 GCSEs A*–C or equivalent; intermediate = 5 or more GCSEs A*–C/vocational GCSE/GNVQ intermediate or equivalent; high = 2 or more A-levels or equivalent.

### Measures

#### JEID scale

The JEID scale comprises five newly developed items that together depict a broad range of earnings—from the lower end, through the middle range, to the upper end of the earnings distribution. To define five earnings categories, we used information on the average earnings of five income groups (gross monthly earnings in euros/pounds), representing the 10th, 50th, 80th, 90th, and 99th percentile of the earnings distribution within the population of Germany/UK. We opted for these five percentiles to capture income groups across the distribution that are clearly distinguishable for respondents and at the same restrict the number of items to the JEID scale allowing for integration into survey programs that face strict time restrictions.

Each income group is described based on three *typical* example occupations within that group. The occupational examples are typical in the sense that they are most prevalent in the respective percentile. Moreover, in order to eliminate potential bias introduced through gender stereotypes, we chose the occupations such that one example occupation was male-dominated, one female-dominated, and one gender-mixed. All contextual information refers to full-time employees (at least 35 hours/week) and is based on the—at the time of conducting the studies—latest versions of the German Socio-Economic Panel (SOEP; Version 34 [45]) from 2017 for the German-language version and on the UK Household Longitudinal Study “Understanding Society” (Wave 9 [46]) from 2017/2018 for the English-language version. Respondents were asked to indicate whether they perceived the earnings of each specific group to be unfairly low, fair, or unfairly high.

We tested different response scale lengths across the first two studies to achieve appropriately differentiated responses while at the same time minimizing cognitive load. We tested a 7-point response scale in Study 1, and a 9-point response scale in Study 2. The leftmost (lowest) scale point was labeled *unfairly low*, the middle scale point was labeled *fair*, the rightmost (highest) scale point was labeled *unfairly high*. The other scale points had no verbal labels. In Study 1 and Study 2, half of the participants (approx. 50% per quota) received a response scale with numerical labels in addition to verbal labels, and the other half received a response scale without numerical labels. In the first condition, numerical labels ranging from –4/–5 to +4/+5 were shown below the verbal labels, with the scale midpoint labeled as 0. There were no statistically significant differences in average responses between the condition without numerical labels and the condition with numerical labels—either for the five items individually or for the overall scale mean. Hence, it was possible to pool responses from the two groups for the main analyses (see S1 Appendix). In our subsequent analyses, we coded the scale points such that the scale ranged from 1 (*ungerecht niedrig/unfairly low*), through 4/5 (*gerecht/fair*), to 7/9 (*ungerecht hoch*/*unfairly high*).

**Fig 1:**
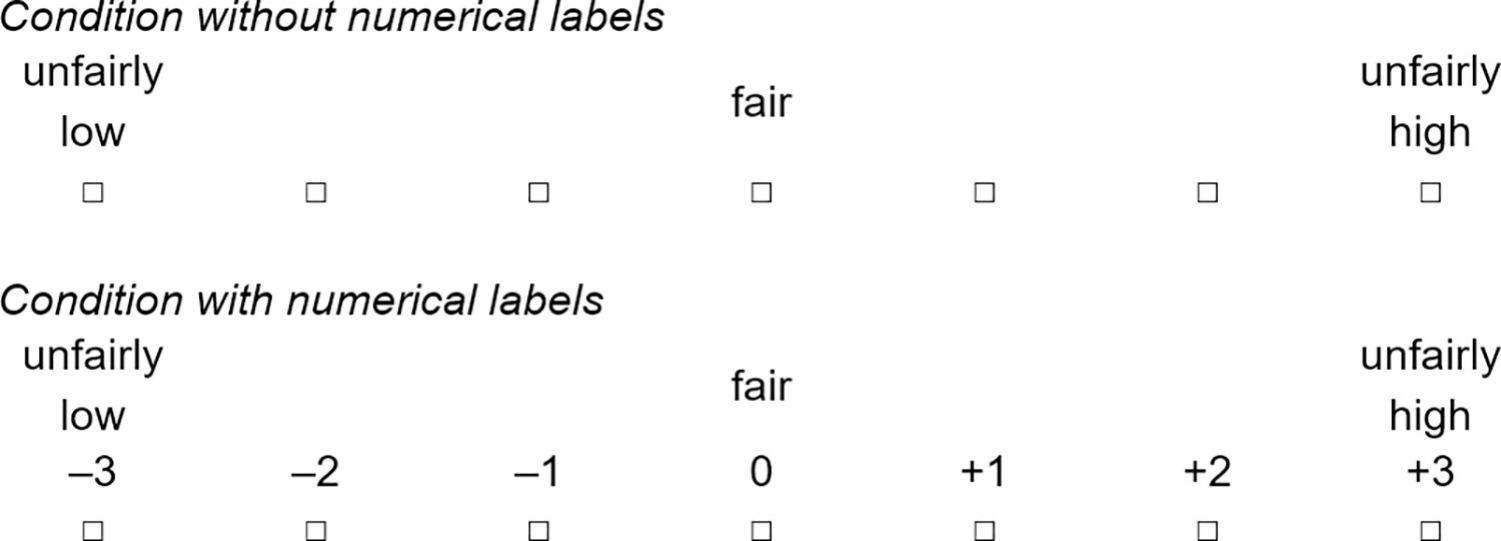
Response scale format of the final JEID scale used in study 3.

Before the items and the response scale were administered in Study 1, a preliminary version underwent cognitive pretesting to optimize them and to assess the length of the response scale, the need for numerical labels, and the amount of and necessity for context-related information in the instruction and the items (for more detailed information, see [47]). During the scale development process, we optimized and slightly adapted the instruction and items as well as the response scale. In Study 3, we used the final JEID scale with a 7-point response scale without numerical labels (see Fig 1).

The final instruction wording, item wordings, and response scale wordings of both language versions of the scale used in Study 3 are presented in Table 2 (the answer sheet of the German-language JEID scale can be found in the S2 Appendix⸺this answer sheet represents the final item wordings and the final optimal response scale used in Study 3; the answer sheet of the English-language JEID scale can be found in the S3 Appendix). The German-language JEID scale was translated into English using the TRAPD (Translation, Review, Adjudication, Pretesting, and Documentation [48]) approach. First, one scale expert (English native speaker) and one professional translator (non-native English speaker) translated the instructions and item wordings and the response scale labels of the JEID scale into English independently of each other. Second, an adjudication meeting was held in which the two translators, a psychological expert, and an expert in questionnaire translation reviewed the translation proposals and developed the final translation. The translation procedure for the JEID scale differed slightly from the full TRAPD approach because one of the initial translations was not provided by a professional translator and the other was not provided by an English native speaker. The completion time for both language versions was 1.50–2.75 min (Germany: *M* = 166.90 s, *SD* = 479.04, *Mdn* = 81.05 s; UK: *M* = 165.44 s, *SD* = 522.03, *Mdn* = 79.00 s; these values are based on the retest of Study 3).

**Table 2:** Wording of the JEID items by language version.

	German-language version	English-language version
Instruction	Nun stellen wir Ihnen ein paar Fragen zu Einkommensunterschieden in Deutschland.	We would now like to ask you a few questions about income differences in the UK.
	Aktuelle Umfrageergebnisse zeigen, dass Geringverdiener in Deutschland durchschnittlich **1.500 Euro** im Monat erhalten. Durchschnittsverdiener erhalten durchschnittlich **2.900 Euro** im Monat, Gutverdiener durchschnittlich **4.100 Euro** und Besserverdiener durchschnittlich **6.700 Euro**. Die Topverdiener in Deutschland erhalten mehr als **11.000 Euro** im Monat.	Current survey results show that low-income earners in the UK make **£1,100** on average per month. Middle-income earners make **£2,100** on average per month, upper-middle-income earners make **£3,300** on average per month, and high-income earners make **£6,000** on average per month. Top-income earners in the UK make more than **£8,500** per month.
	Diese Zahlen beziehen sich auf monatliche **Bruttoeinkommen** von vollzeitbeschäftigten Angestellten. Mit dem Bruttoeinkommen meinen wir das, was jemand, der in **Vollzeit** arbeitet, monatlich vor Abzug von Steuern und Sozialversicherungsbeiträgen verdient.	These figures refer to the **gross monthly income** of full-time employees. By gross income we mean the amount earned per month by someone who works **full-time** before deductions for taxes and social security contributions.
	Im Folgenden möchten wir gerne von Ihnen wissen, wie gerecht Sie diese Einkommen finden.	We would now like to ask you how fair you find these incomes.
Item 1	**Geringverdiener** wie z. B. **Reinigungskräfte, Friseure oder Paketboten** [cleaners, hairdressers, or couriers] verdienen brutto durchschnittlich **1.500 Euro** im Monat. Damit verdienen sie weniger als **90 %** aller Angestellten in Deutschland.	**Low-income earners** such as **cleaners, shop salespeople, or couriers** make **£1,100** (gross) on average per month. This means that they earn less than **90%** of all employees in the UK.
	Finden Sie das Einkommen von Geringverdienern in Deutschland ungerecht niedrig, gerecht oder ungerecht hoch?	Do you think that the income of low-income earners in the UK is unfairly low, fair, or unfairly high?
Item 2	**Durchschnittsverdiener** wie z. B. **Krankenschwestern/Krankenpfleger, Buchhalter oder Elektriker** [nurses, bookkeepers, or electricians] verdienen brutto durchschnittlich **2.900 Euro** im Monat. Damit liegen sie mit ihrem Einkommen im Mittelfeld.	**Middle-income earners** such as **nurses, office clerks, or social workers** make **£2,100** (gross) on average per month. This means that their income is in the mid-range.
	Finden Sie das Einkommen von Durchschnittsverdienern in Deutschland ungerecht niedrig, gerecht oder ungerecht hoch?	Do you think that the income of middle-income earners in the UK is unfairly low, fair, or unfairly high?
Item 3	**Gutverdiener** wie z. B. **Lehrer, Polizisten oder Softwareentwickler** [teachers, police officers, or software developers] verdienen brutto durchschnittlich **4.100 Euro** im Monat. Damit verdienen sie mehr als **80 %** aller Angestellten in Deutschland.	**Upper-middle-income earners** such as **teachers, police officers, or programmers** make **£3,300** (gross) on average per month. This means that they earn more than **80%** of all employees in the UK.
	Finden Sie das Einkommen von Gutverdienern in Deutschland ungerecht niedrig, gerecht oder ungerecht hoch?	Do you think that the income of upper-middle-income earners in the UK is unfairly low, fair, or unfairly high?
Item 4	**Besserverdiener** wie z. B. **Ärzte, Ingenieure oder Universitätsprofessoren** [doctors, engineers, or university professors] verdienen brutto durchschnittlich **6.700 Euro** im Monat. Damit verdienen sie mehr als **90 %** aller Angestellten in Deutschland.	**High-income earners** such as **doctors, engineers, or department managers** make **£6,000** (gross) on average per month. This means that they earn more than **90%** of all employees in the UK.
	Finden Sie das Einkommen von Besserverdienern in Deutschland ungerecht niedrig, gerecht oder ungerecht hoch?	Do you think that the income of high-income earners in the UK is unfairly low, fair, or unfairly high?
Item 5	**Topverdiener** wie z. B. **Geschäftsführer, Bankdirektoren oder Unternehmensberater** [chief executive officers, bank directors, or management consultants] verdienen brutto mehr als **11.000 Euro** im Monat. Damit verdienen sie mehr als **99 %** aller Angestellten in Deutschland.	**Top-income earners** such as **chief executives, bank directors, or management consultants** make more than **£8,500** (gross) per month. This means that they earn more than **99%** of all employees in the UK.
	Finden Sie das Einkommen von Topverdienern in Deutschland ungerecht niedrig, gerecht oder ungerecht hoch?	Do you think that the income of top-income earners in the UK is unfairly low, fair, or unfairly high?

*Note*. The English-language job titles in square brackets in Column 2 are provided for information in this paper only and were not included in the surveys. The question wording presented to respondents uses the broad but very common term “income” (and “Einkommen” in the German-language version) instead of “earnings” in combination with a definition of the underlying income concept.

#### Value orientations

As proposed in Section 2, just rewards in a given situation are guided by the principles of distributive justice, namely, equality, equity, need, and entitlement. Research suggests that people (and societies) differ with regard to which of these distributive rules they believe should guide the allocation of goods and burdens within a society [32, 49, 50]. Accordingly, we expected that such normative orientations coincide with the evaluation of existing inequalities. Preference for normative principles that suggest limiting inequality (such as a preference for the allocation of goods within a society based on the principle of need or equality) would be associated with perceptions of more severe injustice with regard to the extremes of the earnings distribution. By contrast, we expected normative orientations that provide justification for inequality based on the status quo or on individual differences in contributions (such as the distributive principle of entitlement or equity) to be associated with perceptions of less severe injustice. We used two measures of value orientations to test these assumptions.

As a first measure, we used the four subdimensions of the *Basic Social Justice Orientation* (BSJO) scale (both language versions: [32])—namely, need, equity, equality, and entitlement—to assess agreement with each of the five distributive principles. The BSJO scale comprises a total of 12 items (4 items for each subdimension) to be answered on a 5-point rating scale. For the analyses, we coded responses to range from 1 (*strongly disagree*) to 5 (*strongly agree*). The scale has satisfactory psychometric properties [32].

In addition to the assessment of normative preferences (BSJO scale), we used the *Left–Right Self-Placement* scale (both language versions: [51]) to operationalize political preferences. The scale assesses political orientation on a 10-point response scale ranging from 1 (*left*) to 10 (*right*); it is frequently used in social surveys such as the ESS and the German General Social Survey (ALLBUS). In line with the general stance on income inequality associated with the political left and right, we assumed that respondents who tended toward the left would evaluate the observed earnings distribution as more unjust than those who tended toward the right.

#### Sociodemographic characteristics

Justice theory suggests that non-reflexive justice evaluations are not strictly impartial—that is, they are not completely independent of the individual’s own situation [52, 53]. For example, women and individuals with a high level of education have been shown to hold more egalitarian views [32, 54]. There is mixed evidence with regard to age: Whereas Hülle et al. [32] reported that younger respondents expressed more egalitarian views, Forsé and Parodi [55] found the opposite tendency. Following the assumptions of relative deprivation theory [56–58], individuals’ own positions in the earnings distribution may provide an important frame of reference [6], with those at the bottom of the distribution holding more negative evaluations of the existing inequalities and those at the top of the distribution holding more positive evaluations.

Accordingly, we analyzed age, sex, educational attainment, and gross income as antecedents of the justice evaluation of the earnings distribution. *Sex* was operationalized as *male* (1) and *female* (2). *Age* was surveyed in years.

To measure *educational attainment*, we slightly adapted a question used in the ESS [59] and asked for respondents’ highest level of education. After combining similar educational groups, the following categories remained: *no educational qualification/basic school-leaving qualification* (1), *intermediate school-leaving qualification* (2), and *entrance qualification for a university of applied sciences/general higher education entrance qualification* (3).

To assess *gross income*, we combined and adapted two questions, one of which was used in the ESS [59] and the other in ALLBUS [60]. The adapted open-ended item read as follows: “How high is your own gross monthly income (employee: the amount before deductions for tax and social security; self-employed: average gross monthly income before deductions for overhead)?”

#### Social desirability

Income inequality features prominently in public discourse, and its growth and alleged detrimental effects are the subject of regular media coverage [61]. Moreover, political parties and other institutions place the reduction of income inequalities prominently on their agendas (e.g., Goal 10 of the United Nations 2030 Agenda for Sustainable Development, “Reduce inequality within and among countries”). This may suggest that a negative attitude toward income inequality is seen as socially desirable. Therefore, we investigated a possible distortion of respondents’ item responses by social desirability.

We examined the susceptibility of JEID to two aspects of socially desirable responding (exaggerating positive qualities and minimizing negative qualities) with the *Social Desirability–Gamma Short Scale* (KSE-G; German-language version: [62]; English-language version: [63]). KSE-G comprises six items to be answered on a 5-point rating scale ranging from 1 (*does not apply at all*) to 5 (*applies completely*). To ensure that high values represented high levels of socially desirable responding, we recoded the items of the subdimension “minimizing negative qualities.” KSE-G has satisfactory psychometric properties [63] and has been used, for example, in ALLBUS.

### Analytical strategy

Our analyses of JEID comprised three parts. First, we performed descriptive analyses, investigating the response distribution and comparing the distribution between the two response scale lengths (seven vs. nine categories) to identify the best response scale. Second, we investigated psychometric properties of the JEID scale—namely, its objectivity, reliability, and validity. These properties can be used to assess the quality of a scale, and the documentation of an instrument should provide appropriate information about which quality criteria are met.

Third, because the JEID scale comprises five items for the evaluation of five income groups that should not only be considered individually but also together, we examined the internal structure (i.e., different types of justice evaluations) of JEID. We used two methods to determine whether justice evaluations can be divided into different response types: (a) latent profile analysis (LPA) and (b) *k-*means clustering [64]. In both methods, groups are formed based on the response patterns across the five items. LPA aims to identify latent subpopulations within a population at a categorical level, whereas *k*-means clustering aims to partition a sample into different clusters at a manifest level. LPA provides slightly clearer quantitative criteria for choosing the correct number of profiles. By contrast, *k*-means clustering requires the use of different techniques to extract the optimal number of clusters: the elbow method, the average silhouette method [65], and the gap statistic method [66]. The rationale for performing both LPA and the *k*-means clustering was twofold: first, to find clusters that ideally are independent of the chosen method; second, for user-friendliness, because some users will prefer LPA and others *k*-means clustering.

With the exception of LPAs, we ran all statistical analyses with R (Version 4.0.3). We used the following packages: car [67], cluster [68], data.table [69], dplyr [70], factoextra [71], ggplot2 [72], ggridges [73], psych [74], and MplusAutomation [75]. For the LPAs, we used Mplus (Version 8.4 [76]). The code of all analyses can be found in the S4 Appendix.

## Results

### Descriptive statistics

#### Distribution of answers by study

**Fig 2:**
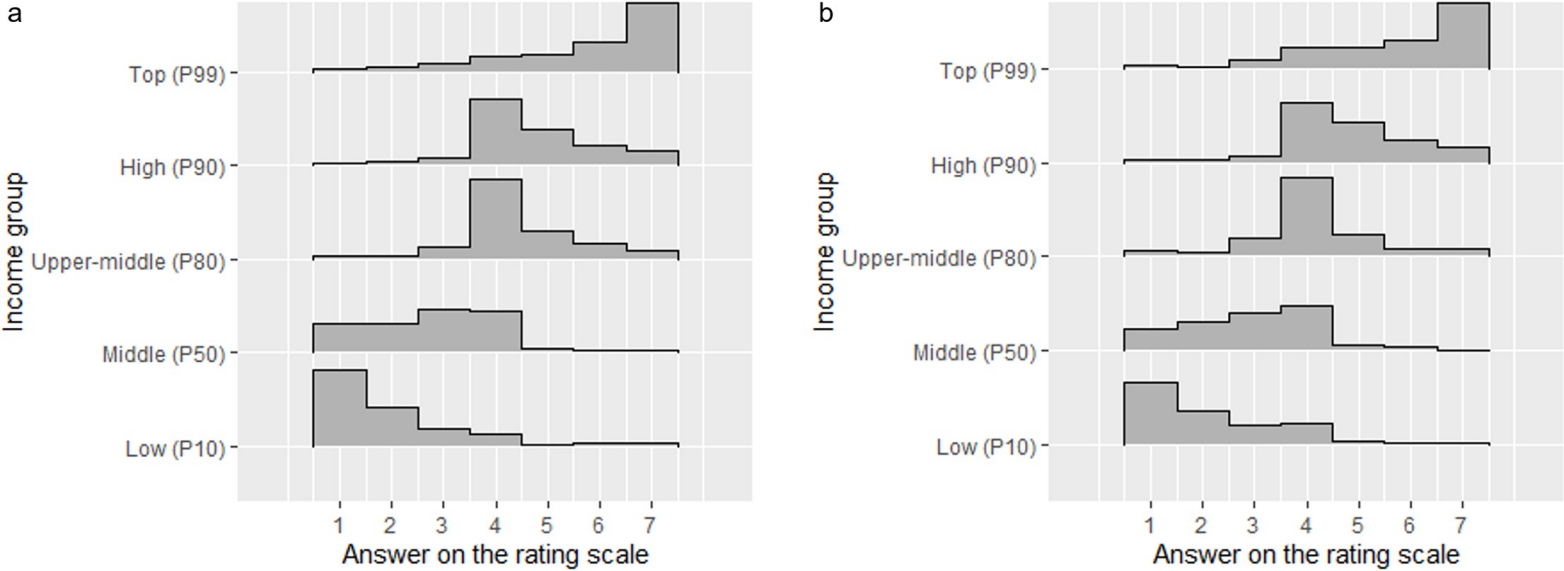
Distribution of the answer categories of the JEID Items for study 3 by country. *Note*. P = percentile. a: Germany (*N =* 420). b: UK (*N =* 440).

In the first step, we took a look at the distribution of answers by study. Table 3 shows the descriptive statistics (i.e., means, standard deviations, skewness, and kurtosis) for the five items as well as for the scale mean, separately for Studies 1, 2, and 3 (Germany and the UK). The distributions of justice evaluations for the five income groups from Study 3 (Germany and the UK) are additionally depicted in Fig 2.

**Table 3:** Descriptive statistics by study for the JEID items.

	*M*				*SD*				Skewness				Kurtosis			
	Study 1	Study 2	Study 3		Study 1	Study 2	Study 3		Study 1	Study 2	Study 3		Study 1	Study 2	Study 3	
Income group			Germany	UK			Germany	UK			Germany	UK			Germany	UK
Scale mean	3.93	4.89	3.92	3.96	0.71	1.01	0.72	0.76	0.02	−0.16	−0.12	−0.22	0.71	1.86	1.69	2.68
Low (P10)	1.96	2.53	2.00	2.23	1.38	1.90	1.41	1.42	1.77	1.38	1.77	1.20	2.98	1.59	2.94	1.08
Middle (P50)	2.87	3.59	2.83	3.02	1.22	1.86	1.26	1.30	0.31	0.44	0.30	0.24	0.20	0.06	0.07	−0.09
Upper-middle (P80)	4.34	5.42	4.39	4.09	1.15	1.59	1.15	1.19	0.14	0.15	0.13	0.05	1.03	0.99	1.02	1.35
High (P90)	4.71	5.77	4.67	4.75	1.19	1.62	1.22	1.19	0.13	0.06	0.14	−0.01	0.20	0.75	0.19	0.44
Top (P99)	5.73	7.17	5.72	5.73	1.59	2.06	1.59	1.49	−1.14	−1.03	−1.19	−1.11	0.42	0.32	0.53	0.65

*Note*. P = percentile. The rating scale ranged from 1 (*unfairly low*) to 7 (*unfairly high*) in Study 1 (*N =* 486) and Study 3 (Germany: *N =* 420, UK: *N =* 440), and from 1 (*unfairly low*) to 9 (*unfairly high*) in Study 2 (*N* = 618).

As the results show, the distributions of answers to the five items and the scale means were comparable across all studies and countries: On average, evaluations tended most toward “unfairly low” for the incomes of low-income earners and “unfairly high” for the incomes of top-income earners. Evaluations generally differed substantially between income groups, with the exception that, in the German samples, the incomes of upper-middle- and high-income earners were rated as almost equally (un)fair. The variance within the evaluation of each item ranged between 1.15 < *SD* < 1.59 (Study 1 and Study 3 [Germany], 7-point scale), 1.19 < *SD* < 1.49 (Study 3 [UK], 7-point scale), and 1.59 < *SD* < 2.06 (Study 2, 9-point scale).

The average evaluation of the incomes of low- and middle-income earners was below the midscale point (i.e., tending toward “unfairly low”), and the average evaluation of the income of the other three income groups was above the midscale point (i.e., tending toward “unfairly high”).

### Psychometric quality criteria

In the second step, we investigated psychometric properties of the JEID scale in both studies—specifically, its objectivity, reliability, and validity.

#### Objectivity

A scale is objective if three conditions are met: objectivity of application (the scale works independently of the administrator), objectivity of evaluation (the scale works independently of the evaluator of the instrument), and objectivity of interpretation (unambiguous and user-independent rules are provided). The JEID scale ensures (a) objectivity of application through the standardized questionnaire format and written instructions; (b) objectivity of evaluation through the fixed scoring rules and labeled response categories; and (c) objectivity of interpretation through the reference ranges presented in the S5 Appendix, which are based on Study 3 (Germany and the UK).

#### Test–retest reliability

As an estimate for the reliability of the JEID scale, we computed the test–retest stability, *r*_tt_, over a period of about 2 weeks in all studies. Because the test–retest stability is sensitive not only to measurement error but also to state fluctuations in the attribute in question [77], test–retest stability is best understood as a lower-bound estimate of reliability. The test–retest correlations are displayed in Table 4.

**Table 4:** Reliability estimates for the JEID items by study.

	Study 1		Study 2		Study 3			
Income group	(*N* = 189)		(*N* = 299)		Germany (*N* = 420)		UK (*N* = 440)	
	*r*_tt_	95% CI	*r*_tt_	95% CI	*r*_tt_	95% CI	*r*_tt_	95% CI
Low (P10)	.61	[.51, .69]	.43	[.33, .51]	.37	[.25, .48]	.65	[.56, .72]
Middle (P50)	.56	[.46, .65]	.51	[.42, .59]	.33	[.20, .45]	.46	[.34, .56]
Upper-middle (P80)	.33	[.20, .45]	.31	[.21, .42]	.36	[.24, .48]	.35	[.22, .47]
High (P90)	.29	[.15, .41]	.43	[.34, .52]	.42	[.30, .53]	.29	[.16, .41]
Top (P99)	.44	[.31, .54]	.46	[.36, .54]	.42	[.29, .52]	.42	[.30, .53]

*Note*. CI = confidence interval; P = percentile.

The test–retest reliability for the five individual items (i.e., income groups) of the JEID scale was moderate for Germany and the UK. However, because the five items belong together, the reliability of the entire scale should also be considered. Therefore, we also analyzed the total reliability across all items using a within-person profile similarity (i.e., the correlation of each person’s response patterns across the two measurement occasions), and we aggregated this correlation across the entire samples. The results showed that the 7-point response scale (Studies 1 and 3: *r* = .73; UK: *r* = .71) proved to be more reliable over time than the 9-point response scale (Study 2: *r* = .66) and sufficient for research purposes [78]. Overall, the profile similarity results indicated that the response patterns across all items were quite stable between test and retest.

#### Validity evidence based on the relationship between scores on the JEID scale and on scales measuring other variables

We examined evidence based on the relationship between scores on the items of the JEID scale and on scales measuring other variables described in the Method section. As we computed this kind of evidence based on manifest (scale) scores, the reported correlations (see Table 5) are subject to attenuation and represent the lower bound of the true associations. The interpretation of the correlation coefficients is based on the meta-analytic effect size guidelines for individual differences proposed by Gignac and Szodorai [79]: relatively small effects (*r* ≥ .10), typical (medium) effects (*r* ≥ .20), and relatively large effects (*r* ≥ 30). According to these guidelines, a correlation of *r* = .19 corresponds to the 50th percentile of a meta-analytical distribution of correlations in individual differences research. For this reason, we highlight medium to large effects in bold in Table 5.

**Table 5:** Correlations of the JEID items with other relevant measures by study.

	Study 1		Study 2		Study 3			
Variable Income group					Germany		UK	
	*r*	95% CI	*r*	95% CI	*r*	95% CI	*r*	95% CI
*Value orientations*								
Basic social justice orientation^a,c^								
Need								
Low (P10)	**–.26**	[–.34,–.18]	**–.32**	[–.40,–.23]	-		–.17	[–.26,–.08]
Middle (P50)	–.18	[–.27,–.09]	**–.22**	[–.31,–.13]	-		–.09	[–.18, .00]
Upper-middle (P80)	–.00	[–.09, .09]	.06	[–.04, .15]	-		–.06	[–.15, .03]
High (P90)	.09	[–.00, .17]	.07	[–.03, .16]	-		.00	[–.09, .10]
Top (P99)	.14	[–.05, .23]	**.25**	[.16, .34]	-		.18	[.09, .27]
Equity								
Low (P10)	–.00	[–.09, .09]	.06	[–.03, .16]	-		.10	[.01, .20]
Middle (P50)	.02	[–.07, .10]	.04	[–.06, .13]	-		.11	[.02, .20]
Upper-middle (P80)	–.01	[–.10, .08]	–.01	[–.10, .09]	-		.07	[–.02, .17]
High (P90)	–.05	[–.14, .04]	–.05	[–.15, .05]	-		–.06	[–.15, .04]
Top (P99)	–.09	[–.18,–.01]	–.02	[–.12, .08]	-		–.03	[–.12, .06]
Equality								
Low (P10)	**–.25**	[–.33,–.16]	**–.30**	[–.39,–.21]	-		–.17	[–.26,–.08]
Middle (P50)	–.10	[–.18,–.01]	**–.20**	[–.29,–.10]	-		–.03	[–.12, .07]
Upper-middle (P80)	.11	[.02, .20]	.13	[.04, .22]	-		.03	[–.07, .12]
High (P90)	**.21**	[.12, .29]	.18	[.08, .27]	-		.05	[–.04, .14]
Top (P99)	.18	[.09, .27]	.19	[.10, .28]	-		.11	[.01, .20]
Entitlement								
Low (P10)	.12	[.03, .21]	**.22**	[.13, .31]	-		**.22**	[.13, .30]
Middle (P50)	.06	[–.03, .14]	.19	[.10, .29]	-		.19	[.10, .28]
Upper-middle (P80)	–.14	[–.22,–.05]	–.07	[–.16, .03]	-		.09	[–.00, .18]
High (P90)	–.18	[–.27,–.09]	–.13	[–.22,–.03]	-		–.11	[–.20,–.01]
Top (P99)	**–.27**	[–.35,–.19]	**–.30**	[–.39,–.21]	-		**–.21**	[–.30,–.12]
Left–right placement^b^								
Low (P10)	.15	[.05, .24]	.15	[.07, .23]	.05	[–.08, .17]	**.32**	[.20, .44]
Middle (P50)	.14	[.05, .24]	**.20**	[.12, .28]	.06	[–.06, .19]	**.33**	[.21, .45]
Upper-middle (P80)	–.08	[–.17, .02]	.10	[.01, .18]	.09	[–.03, .22]	.16	[.02, .29]
High (P90)	–.12	[–.22,–.03]	.02	[–.07, .10]	.03	[–.10, .16]	–.04	[–.18, .09]
Top (P99)	–.10	[–.20,–.01]	–.13	[–.21,–.05]	–.01	[–.14, .11]	**–.22**	[–.34,–.09]
*Sociodemographic characteristics*								
Female								
Low (P10)	–.02	[–.11, .07]	–.12	[–.20,–.04]	.00	[–.09, .10]	–.16	[–.25,–.07]
Middle (P50)	–.10	[–.18,–.01]	–.08	[–.15, .00]	–.04	[–.13, .06]	–.19	[–.28,–.10]
Upper-middle (P80)	.04	[–.05, .13]	.03	[–.04, .11]	.09	[–.00, .18]	–.10	[–.19,–.01]
High (P90)	–.02	[–.11, .07]	–.01	[–.09, .07]	.03	[–.06, .13]	.04	[–.06, .13]
Top (P99)	.03	[–.06, .12]	.07	[–.00, .15]	.09	[–.01, .18]	.08	[–.02, .17]
Age								
Low (P10)	**–.20**	[–.29,–.12]	**–.25**	[–.32,–.17]	–.18	[–.27,–.09]	–.12	[–.21,–.03]
Middle (P50)	–.14	[–.23,–.05]	**–.22**	[–.29,–.14]	–.06	[–.15, .04]	–.03	[–.12, .07]
Upper-middle (P80)	–.15	[–.23,–.06]	–.03	[–.11, .05]	.01	[–.08, .11]	–.10	[–.19,–.00]
High (P90)	–.03	[–.12, .06]	–.00	[–.08, .08]	.06	[–.03, .16]	.03	[–.06, .13]
Top (P99)	.06	[–.03, .15]	.16	[.08, .23]	.15	[.05, .24]	.06	[–.03, .15]
Educational attainment								
Low (P10)	.14	[.06, .23]	.06	[–.02, .14]	.08	[–.01, .18]	.07	[–.02, .16]
Middle (P50)	.10	[.01, .19]	.01	[–.07, .09]	–.01	[–.11, .08]	–.09	[–.18, .00]
Upper-middle (P80)	–.04	[–.12, .05]	–.03	[–.11, .05]	–.14	[–.23,–.05]	–.11	[–.21,–.02]
High (P90)	–.05	[–.14, .04]	–.02	[–.10, .06]	–.03	[–.13, .07]	–.02	[–.11, .07]
Top (P99)	–.07	[–.16, .02]	–.01	[–.09, .07]	.00	[–.09, .10]	.08	[–.01, .17]
Gross income								
Low (P10)	.08	[–.02, .18]	.01	[–.08, .10]	.04	[–.07, .15]	.12	[.01, .23]
Middle (P50)	–.01	[–.11, .08]	.07	[–.02, .16]	–.02	[–13, .08]	.08	[–.03, .19]
Upper-middle (P80)	–.07	[–.17, .03]	.04	[–.05, .13]	–.03	[–.14, .08]	–.02	[–.13, .09]
High (P90)	–.09	[–.19, .01]	.01	[–.08, .10]	–.04	[–.14, .07]	–.05	[–.16, .06]
Top (P99)	–.12	[–.22,–.02]	.01	[–.08, .10]	–.10	[–.20, .01]	–.02	[–.13, .09]
*Social desirability* ^a,c^								
Exaggerating positive qualities								
Low (P10)	–.14	[–.23,–.05]	**–.22**	[–.31,–.12]	-		.06	[–.03, .15]
Middle (P50)	–.12	[–.21,–.03]	–.11	[–.20,–.01]	-		.08	[–.01, .17]
Upper-middle (P80)	.04	[–.05, .13]	.04	[–.05, .14]	-		.07	[–.03, .16]
High (P90)	.14	[.05, .22]	.02	[–.08, .11]	-		–.02	[–.11, .07]
Top (P99)	.14	[.05, .23]	.16	[.06, .25]	-		–.02	[–.11, .07]
Minimizing negative qualities								
Low (P10)	–.18	[–.26,–.09]	–**.35**	[–.43,–.26]	-		**–.29**	[–.37,–.20]
Middle (P50)	–.14	[–.23,–.06]	–**.22**	[–.31,–.12]	-		**–.26**	[–.34,–.17]
Upper-middle (P80)	–.02	[–.10, .07]	.06	[–.04, .15]	-		–.19	[–.28,–.10]
High (P90)	.08	[–.01, .17]	.12	[.03, .22]	-		.01	[–.08, .11]
Top (P99)	.17	[.08, .26]	**.28**	[.18, .36]	-		.18	[.08, .27]

*Note*. P = percentile. Coefficients with *r* ≥ |.20| are in bold type. Study 1—*N =* 486 (*N*_Basic social justice orientation_ = 485; *N*_Left–right placement_ = 425; *N*_Gross income_ = 393; *N*_Trust in government_ = 461). Study 2—*N* = 618 (*N*_Basic social justice orientation_ = 414; *N*_Left–right placement_ = 544; *N*_Gross income_ = 470; *N*_Trust in government_ = 389; *N*_Social desirability_ = 417). Study 3: Germany—*N* = 420 (*N*_Left–right placement_ = 234; *N*_Gross income_ = 342); UK—*N* = 440 (*N*_Left–right placement_ = 211; *N*_Gross income_ = 320).

^a^ For these variables, only two thirds of the sample in Study 2 were surveyed due to the planned-missingness design.

^b^ For these variables, only two thirds of the sample in Study 3 (Germany and the UK) were surveyed due to the planned-missingness design.

^c^ For these variables, each respondent received only two thirds of items within one scale and subscale in the UK sample due to the planned-missingness design. Therefore, the reported correlations may underestimate or overestimate the true associations.

Although the JEID response scale is not linear, and it ranges from “unfairly low,” through “fair,” to “unfairly high,” we can still interpret the linear correlations in a meaningful way. This is because—as can be seen from the item distributions (see Section 5.1.1)—for each item, almost all individuals fell either between “unfairly low” and the midpoint (“fair”) or between the midpoint and “unfairly high.” Thus, it is always clear what a positive/negative correlation means—namely, a shift toward the midpoint or away from the midpoint.

JEID correlated with basic social justice orientations and political orientations. We observed the strongest correlations for three of the four principles of distributive justice measured with the BSJO scale: Respondents who tended to prefer principles according to which benefits and burdens should be distributed equally (equality principle) or based on individual needs (need principle) were more likely to evaluate the income of low- and middle-income earners as “unfairly low” and the income of (high- and) top-income earners as “unfairly high.” In other words, they evaluated inequality as more unjust. By contrast, respondents who showed stronger support for the entitlement principle, according to which the distribution of benefits and burdens should be based on status, tended to evaluate the income of low- and middle-income earners more positively (i.e., as fairer). These respondents also perceived lower levels of overreward for the income of high- and top-income earners, thus showing an overall higher tolerance for inequality.

In addition, the further respondents moved from left to right on the political orientation scale, the less they tended to rate the extremes of the earnings distribution as unfair. This association, which was particularly strong in the UK, is also in line with evidence showing that liberals hold less system-justifying attitudes than conservatives [80].

Turning to sociodemographic characteristics, there were no differences in justice evaluations of the earnings distribution by sex for any of the five items of the JEID scale in the German samples. By contrast, in the UK sample, we found that women were more likely to evaluate low to upper-middle incomes more negatively, tending toward “unfairly low.” The same was true of older respondents—especially in the German samples—who tended to rate low (and middle) incomes as “unfairly low.” This is in line with studies that have found women and older people to hold more egalitarian views [55]. There were no consistent associations between JEID and educational attainment and income across the studies. This might indicate that individuals’ justice evaluations of the earnings distribution do not reflect their own positions in the income hierarchy, but rather are shaped by normative preferences as to how goods and burdens should be allocated within a society.

Furthermore, JEID showed small-to-large correlations with both subdimensions of social desirability responding in Germany (in the UK, there were associations only with minimizing negative qualities). *Exaggerating positive qualities* depicts the self-deceptive enhancement component of communion-induced socially desirable responding, whereas *minimizing negative qualities* depicts the impression-management component [63]. Both subdimensions were related to evaluations of the incomes of low- and middle-income earners as “unfairly low” and evaluations of the incomes of top-income earners as “unfairly high,” indicating that evaluating inequality as unjust is seen as socially desirable in Germany and the UK.

### Internal structure: Types of justice evaluations

In the fourth step, we examined evidence based on the internal structure of JEID (i.e., different types of justice evaluations). They suggest three distinct response profiles in Studies 1 and 2. The small groups (i.e., profile shares) identified in solutions with a higher number of profiles can be assigned to the three larger profiles because they differ only in the intercept and slope, not in the profile. The results of the LPAs can be found in the S6 and S7 Appendices, separately for the three studies. Given the similar pattern for Studies 1 and 2, we conducted LPAs in a confirmatory fashion in Study 3, testing only up to three profile solutions by means of multigroup LPA models with measurement equivalence across countries in all items and equal latent profile probabilities across countries. As the results show, the three-profile solution from Studies 1 and 2 could be replicated in Study 3 with similar response patterns.

**Fig 3:**
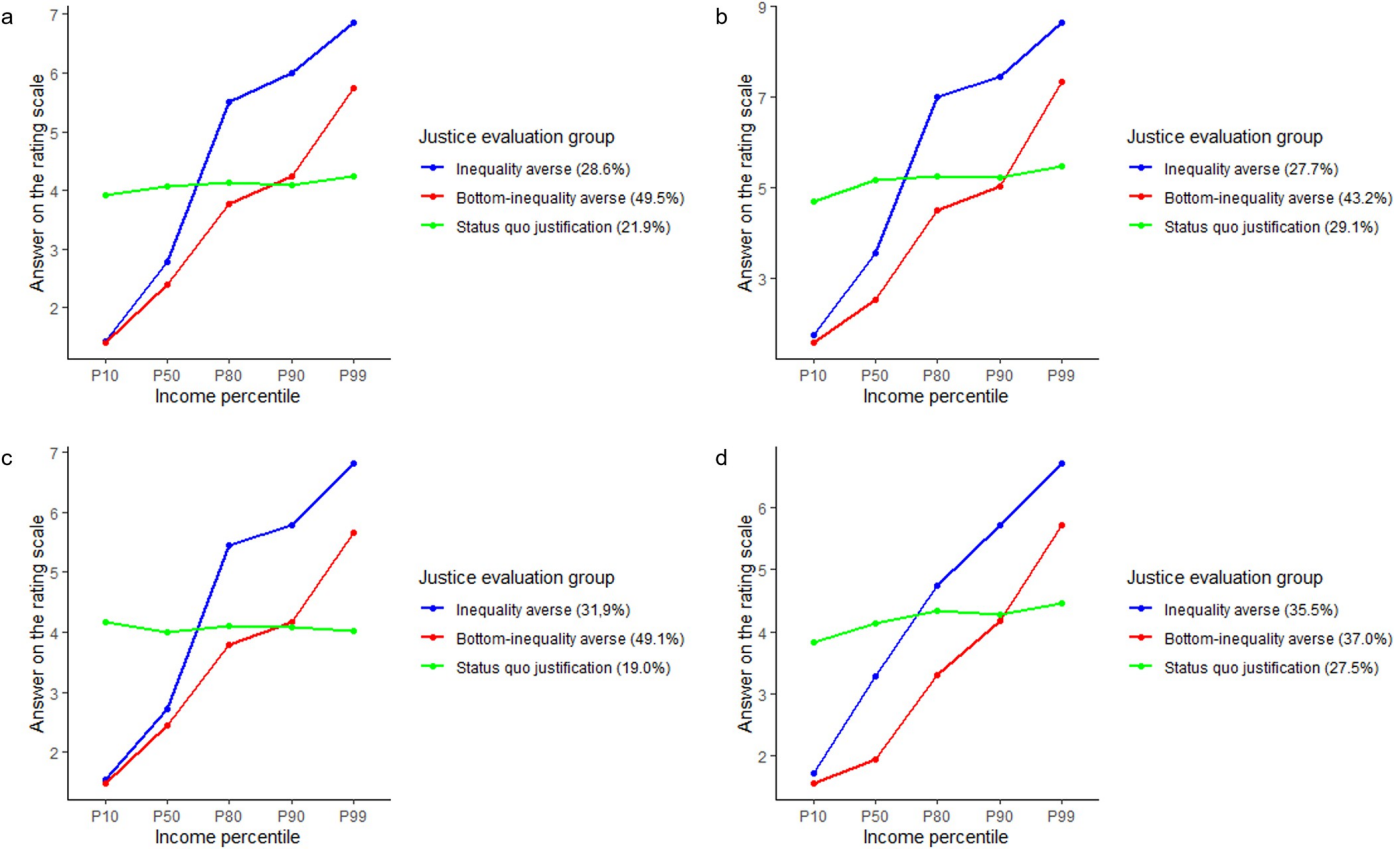
Average answers of the extracted clusters by study. *Note*. a: Study 1. The rating scale ranged from 1 (*unfairly low*) to 7 (*unfairly high*). *n*_Inequality averse_ = 139, *n*_Bottom-inequality averse_ = 240, *n*_Status quo justification_ = 106. b: Study 2. The rating scale ranged from 1 (*unfairly low*) to 9 (*unfairly high*). *n*_Inequality averse_ = 171, *n*_Bottom-inequality averse_ = 267, *n*_Status quo justification_ = 180. c: Study 3 (Germany). The rating scale ranged from 1 (*unfairly low*) to 7 (*unfairly high*). *n*_Inequality averse_ = 134, *n*_Bottom-inequality averse_ = 206, *n*_Status quo justification_ = 80. d: Study 3 (UK). The rating scale ranged from 1 (*unfairly low*) to 7 (*unfairly high*). *n*_Inequality averse_ = 156, *n*_Bottom-inequality averse_ = 163, *n*_Status quo justification_ = 121.

The *k*-means clustering showed a similar pattern: S8 Appendix displays the results for these three techniques by study. Using the elbow method, the average silhouette method, and the gap statistic method, three groups belonged to the optimal number of clusters among all three approaches in Study 2. We extracted the same three groups for all three studies. Fig 3 depicts the average answers of each extracted justice evaluation group for the five income groups separately for Study 1 (Fig 3A), Study 2 (Fig 3B), and Study 3 (Germany: Fig 3C, UK: Fig 3D). The figures show that the extracted clusters are comparable across all studies.

The first group, which we labeled *inequality averse*, tended to evaluate the bottom end of the earnings distribution as “unfairly low” and the incomes at the upper-middle to the top end of the distribution as “unfairly high,” indicating an injustice perception with regard to both the very bottom and the top end of the earnings distribution. This group accounted for almost one third of the respondents in all German samples and slightly more than one third in the UK sample.

The second group, which we labeled *bottom-inequality averse*, displayed a tendency to evaluate the bottom end of the earnings distribution as “unfairly low,” the middle- and upper-middle-income groups as “fair,” and only the top end as “unfairly high.” Thus, in contrast to the first group, this group—which accounted for almost half of the respondents in all German samples and slightly more than one third of respondents in the UK sample—perceived only very low incomes as “unfairly low” and only extremely high incomes as “unfairly high.”

The third extracted group, which we labeled *status quo justification*, evaluated the entire earnings distribution as more or less “fair,” and therefore tended to accept the status quo as “fair.” This group accounted for between a quarter and just under one third of respondents in all samples (German and UK) and showed only a very slight tendency to evaluate the income of the lowest income group as slightly “unfairly low” and the income of the highest income group as slightly “unfairly high.” This slight tendency was more pronounced in Study 2 and in the UK compared to Studies 1 and Study 3.

A more detailed comparison between the three groups in the German samples and the UK sample revealed two minor but nevertheless noteworthy differences. First, we found that all groups accounted for a more or less equal proportion of the respondents in the UK sample, whereas the percentage distribution among the three groups varied in the German samples. In Germany, the largest share (around 43–50%) of respondents belonged to the *bottom-inequality averse* group. Second, the response profiles of the *bottom-inequality averse* and the *status quo justification* groups were almost identical across all studies (Germany and the UK). By contrast, the *inequality averse* group showed a similar evaluation for the 80th and 90th percentile in all three German samples, producing a plateau in the profile, whereas in the UK sample, the profile of this group showed a positive monotone trend, evaluating higher incomes as tending more in the direction of unfair overreward.

Interestingly, both the three-profiles solution of the LPAs and of *k*-means clustering led to three very similar patterns. The only group that looked slightly different was the bottom-inequality averse group. In the case of the LPAs, this group tended to rate the income of top-income earners as “fair,” whereas in the *k*-means clusters, it was rated as “unfairly high.” Thus, we were able to replicate the three clusters or profiles representing different response patterns across the five JEID items with different methods (LPA and *k*-means clustering), different datasets (Study 1, Study 2, and Study 3), and in two different countries (Germany and the UK). From this, we conclude that the three clusters represent robust, replicable groups that differ in their justice evaluations of the earnings distribution. Hence, the items of JEID can be understood as a joint measure of the justice evaluation of the earnings distribution, which can be captured by (latent) profiles.

## Discussion and conclusion

The aim of the present study was to develop and validate a German-language and an English-language scale measuring the subjective justice evaluation of the actual earnings distribution. It is the first measure that allows researchers (a) to capture subjective evaluations of the earnings distribution that are linked to the actual earnings differences within a society and (b) at the same time, to identify segments of the earnings distribution at which people perceive a so-called justice deficit/gap [19]. Such a scale is especially relevant because, first, previous research suggests that subjective perceptions of income inequality are only weakly related to actual levels of inequality [81], and, second, subjective evaluations of objective inequalities are assumed to play an important role in the link between the actual level of inequality and its individual- and societal-level outcomes [7]. We addressed this need for a measurement instrument that links justice evaluations of the earnings distribution to the actual differences in earnings by proposing the five-item JEID scale, which provides respondents with information on the actual earnings distribution/inequality within a given country (here: Germany and the UK).

The JEID scale builds on the justice evaluation function and provides a measure analogous to existing measures assessing the justice of own earnings, which are widely used in empirical justice research [26]. Moreover, it links respondents’ evaluations to context information about average gross earnings and typical occupations, thereby making the justice evaluation of the earnings of five income groups at different segments of the earnings distribution comparable across individuals. Our results are based on three comprehensive samples representing the heterogeneity of the adult population in Germany and one comprehensive sample representing the heterogeneity of the adult population in the UK. Testing various response scale formats, we found that a 7-point and a 9-point rating scale performed almost equally well. Accordingly, either format may be used for the JEID scale. However, in keeping with the goal of optimizing precision and minimizing cognitive load, we recommend using a 7-point response scale without numerical values (i.e., the response scale we used in Study 3 in Germany and the UK).

Within the retest subsamples, JEID showed moderate test–retest reliabilities, thus indicating that each JEID item may not constitute a stable attitude in its own right, but rather may be subject to situational fluctuations. However, the results of the profile similarity across all five items showed substantial stability of the response profiles, especially for the recommended 7-point response scale in both language versions. Furthermore, answers followed a normal distribution across all five items: In all studies and in both countries, respondents tended to rate the bottom end of the earnings distribution as “unfairly low,” the upper-middle income group as “fair,” the top end as “unfairly low,” and the other groups in between.

Furthermore, as expected, in Germany and the UK, JEID was correlated in theoretically plausible ways with normative preferences and political stance. Egalitarian views were associated with stronger injustice perceptions at both ends of the earnings distribution, whereas entitlement views (status as a guiding principle for allocation) were linked to stronger injustice tolerance across the entire earnings distribution. By contrast, sociodemographic characteristics pertaining to individuals’ own positions in the social stratification hierarchy seemed less relevant for justice evaluations of the earnings distribution across both countries underlining the nature of JEID as a non-reflexive (i.e., other-directed) evaluation. In addition, correlations with social desirability suggested the susceptibility for social desirability responding at the extremes of the earnings distribution in both countries.

Because the five JEID items belong to the same underlying construct (i.e., the justice evaluation of the actual earnings distribution), we determined whether justice evaluations across the five items correspond to underlying response types by means of latent profile and cluster analysis. Across all five items, we were able to identify three distinct response patterns, which we labeled *inequality averse* (injustice evaluations across the whole earnings distribution), *bottom-inequality averse* (injustice evaluations only at the endpoints of the earnings distribution, with a focus on the lowest earnings), and *status quo justification* (no injustice evaluations across the entire earnings distribution captured). These response patterns proved to be a robust and multiply replicable structure with converging results obtained by both *k*-means clustering and LPAs as well as across three independent datasets in Germany and one dataset in the UK. The consistent response profiles underline that the five justice evaluations are not isolated evaluations but relate to a broader underlying concept, namely, the justice evaluation of the earnings distribution. Researchers who apply the JEID scale could use these profiles in further analyses—for example, to tackle the question of whether respondents in the *status quo justification* group also react indifferently to other forms of inequality. Another possibility would be to explore whether individuals in the *bottom-inequality aversion* group support anti-poverty policies but oppose redistributive policies.

With the JEID scale, we present a novel instrument that has the potential to fill an important gap in the methodological toolbox of social scientists interested in studying the inequality–evaluation–consequences link. By providing reference information on various income groups, the JEID scale captures respondents’ assessment of the actual earnings distribution rather than eliciting evaluations based on common misperceptions of the extent of earnings inequality. Providing information on typical occupations further acknowledges that individuals differentiate between illegitimate and legitimate inequalities based on deservingness and merit. Moreover, the JEID scale allows researchers to identify segments of the earnings distribution where injustice perceptions with regard to earnings inequality arise, thereby highlighting potential justice deficits. However, previous research has argued that just earnings and just inequality may be inconsistent [27, 82]. That is, if an observer of justice determined a just income for every person in a social aggregate, the corresponding distribution may still not be considered just. Accordingly, JEID does not allow conclusions to be drawn about a level of inequality that respondents will perceive as just but rather provides insights into justice deficits in the actual earnings distribution.

Three limitations—two relating to the JEID scale and one relating to the present studies—should be pointed out. First, because society is constantly changing, the context information provided must be updated at regular intervals. This is especially true for the information on earnings, whereas occupations can be assumed to be relatively stable across time. Second, interpretation of JEID is limited to the specific income concept used (e.g., earnings). Future research may provide respondent with information on the distribution of incomes encompassing also the work of self-employed and other sources than labor (e.g., pension, social assistance, and capital income). Third, our studies were based on quota samples and restricted to respondents in a Web-based survey (CASI). Although quota samples are superior to highly selective convenience samples in that they represent the heterogeneity of the population, they are non-random. Thus, we cannot generalize our findings to the entire German and UK populations, respectively. Further research using representative samples is thus warranted, and adaptation of the instrument to other survey modes is generally conceivable.

To conclude, in the present paper, we presented a German-language and an English-language version of the newly developed Justice Evaluation of the Income Distribution (JEID) Scale. Our validation studies show that individuals’ justice evaluation of the earnings distribution can be measured by the subjective evaluation of five exemplary income groups along the distribution. Across all analyses, our results were very similar in both Germany and the UK, indicating a comparable justice evaluation of the earnings distribution in these two countries. The scale is recommended for use in self-report surveys.

## Supporting information

S1 Appendix Response scale format by condition and study. (PDF)

S2 Appendix Answer sheet of the JEID scale (German-language version). (PDF)

S3 Appendix Answer sheet of the JEID scale (English-language version, UK). (PDF)

S4 Appendix R code for analysis. (PDF)

S5 Appendix Reference ranges of the JEID items based on study 3 for the total population and for sex and age groups, separately for Germany and the UK. (PDF)

S6 Appendix Results of the latent profile analyses by study. (PDF)

S7 Appendix Sample mean distribution of the JEID items resulting from latent profile analysis, by profile number and study. (PDF)

S8 Appendix Results of three methods to extract the optimal number of clusters, by study. (PDF)
