# Supplementary material for: Justice Evaluation of the Income Distribution (JEID): Development and validation of a short scale for the subjective assessment of objective differences in earnings
Source: PLoS One. 2023 Jan 26;18(1):e0281021. doi: 10.1371/journal.pone.0281021 (PMC9879472; doi:10.1371/journal.pone.0281021)
Supplement: S1 Appendix — (PDF) [file pone.0281021.s001.pdf]

Nießen, Adriaans, Liebig, & Lechner (2023). Justice Evaluation of the Income Distribution (JEID): Development and validation of a short scale for the subjective assessment of objective differences in earnings. *Plos One*.

S1 Appendix

Figure S1

Response Scale Format by Condition and Study

a

Condition without numerical labels

unfairly low				fair				unfairly high
□	□	□	□	□	□	□	□	□

Condition with numerical labels

unfairly low				fair				unfairly high
–4	–3	–2	–1	0	+1	+2	+3	+4
□	□	□	□	□	□	□	□	□

b

Condition without numerical labels

unfairly low			fair			unfairly high
□	□	□	□	□	□	□

Condition with numerical labels

unfairly low			fair			unfairly high
–3	–2	–1	0	+1	+2	+3
□	□	□	□	□	□	□

Note. a: Study 1. b: Study 2.

Table S1

Descriptive Statistics and t *Tests by Study and Response Scale Format for the JEID Items*

	M		*SD*		Skewness		Kurtosis		*t* test	
Income group	A	B	A	B	A	B	A	B	*t*	*p*
Study 1										
Scale mean	3.89	3.96	0.69	0.72	−0.09	0.10	−0.01	1.26	–1.06	.290
Low (P10)	1.87	2.05	1.24	1.51	1.74	1.69	3.15	2.39	–1.48	.140
Middle (P50)	2.88	2.87	1.13	1.31	0.12	0.42	0.14	0.12	0.04	.970
Upper-middle (P80)	4.36	4.33	1.13	1.18	0.29	0.01	0.94	1.04	0.34	.734
High (P90)	4.67	4.76	1.21	1.18	0.13	0.14	0.18	0.19	–0.80	.425
Top (P99)	5.68	5.79	1.60	1.57	−1.11	−1.16	0.37	0.45	–0.77	.440
Study 2										
Scale mean	4.87	4.92	0.96	1.07	−0.22	−0.14	2.36	1.40	–0.57	.568
Low (P10)	2.58	2.48	1.90	1.91	1.31	1.46	1.44	1.73	0.64	.522
Middle (P50)	3.55	3.63	1.84	1.88	0.38	0.51	−0.13	0.20	–0.48	.633
Upper-middle (P80)	5.35	5.49	1.49	1.70	0.09	0.16	1.41	0.58	–1.08	.279
High (P90)	5.69	5.84	1.52	1.72	0.15	−0.03	1.11	0.42	–1.22	.262
Top (P99)	7.18	7.15	1.99	2.13	−1.01	−1.04	0.38	0.21	0.16	.876

Note. A = response scale without additional numerical labels; B = response scale with numerical labels; P = percentile. The rating scale ranged from 1 (*unfairly low*) to 7 (*unfairly high*) in Study 1 (N = 486; n_A = 243, n_B = 243) and from 1 (*unfairly low*) to 9 (*unfairly high*) in Study 2 (N = 618; n_A = 321, n_B = 297).
