# Supplementary material for: Justice Evaluation of the Income Distribution (JEID): Development and validation of a short scale for the subjective assessment of objective differences in earnings
Source: PLoS One. 2023 Jan 26;18(1):e0281021. doi: 10.1371/journal.pone.0281021 (PMC9879472; doi:10.1371/journal.pone.0281021)
Supplement: S2 Appendix — (PDF) [file pone.0281021.s002.pdf]

Nießen, Adriaans, Liebig, & Lechner (2023). Justice Evaluation of the Income Distribution (JEID): Development and validation of a short scale for the subjective assessment of objective differences in earnings. *Plos One*.

S2 Appendix: Answer Sheet of the JEID Scale (German-Language Version)

Nun stellen wir Ihnen ein paar Fragen zu Einkommensunterschieden in Deutschland.

Aktuelle Umfrageergebnisse zeigen, dass Geringverdiener in Deutschland durchschnittlich **1.500 Euro** im Monat erhalten. Durchschnittsverdiener erhalten durchschnittlich **2.900 Euro** im Monat, Gutverdiener durchschnittlich **4.100 Euro** und Besserverdiener durchschnittlich **6.700 Euro**. Die Topverdiener in Deutschland erhalten mehr als **11.000 Euro** im Monat.

Diese Zahlen beziehen sich auf monatliche **Bruttoeinkommen** von vollzeitbeschäftigten Angestellten.[1] Mit dem Bruttoeinkommen meinen wir das, was jemand, der in **Vollzeit** arbeitet, monatlich vor Abzug von Steuern und Sozialversicherungsbeiträgen verdient.

Im Folgenden möchten wir gerne von Ihnen wissen, wie gerecht Sie diese Einkommen finden.

	ungerecht niedrig			gerecht			ungerecht hoch
**Geringverdiener** wie z. B. **Reinigungskräfte, Friseure oder Paketboten** verdienen brutto durchschnittlich **1.500 Euro** im Monat. Damit verdienen sie weniger als **90 %** aller Angestellten in Deutschland.<br>Finden Sie das Einkommen von Geringverdienern in Deutschland ungerecht niedrig, gerecht oder ungerecht hoch?	☐	☐	☐	☐	☐	☐	☐
**Durchschnittsverdiener** wie z. B. **Krankenschwestern/Krankenpfleger, Buchhalter oder Elektriker** verdienen brutto durchschnittlich **2.900 Euro** im Monat. Damit liegen sie mit ihrem Einkommen im Mittelfeld.<br>Finden Sie das Einkommen von Durchschnittsverdienern in Deutschland ungerecht niedrig, gerecht oder ungerecht hoch?	☐	☐	☐	☐	☐	☐	☐
**Gutverdiener** wie z. B. **Lehrer, Polizisten oder Softwareentwickler** verdienen brutto durchschnittlich **4.100 Euro** im Monat. Damit verdienen sie mehr als **80 %** aller Angestellten in Deutschland.<br>Finden Sie das Einkommen von Gutverdienern in Deutschland ungerecht niedrig, gerecht oder ungerecht hoch?	☐	☐	☐	☐	☐	☐	☐
**Besserverdiener** wie z. B. **Ärzte, Ingenieure oder Universitätsprofessoren** verdienen brutto durchschnittlich **6.700 Euro** im Monat. Damit verdienen sie mehr als **90 %** aller Angestellten in Deutschland.<br>Finden Sie das Einkommen von Besserverdienern in Deutschland ungerecht niedrig, gerecht oder ungerecht hoch?	☐	☐	☐	☐	☐	☐	☐
**Topverdiener** wie z. B. **Geschäftsführer, Bankdirektoren oder Unternehmensberater** verdienen brutto mehr als **11.000 Euro** im Monat. Damit verdienen sie mehr als **99 %** aller Angestellten in Deutschland.<br>Finden Sie das Einkommen von Topverdienern in Deutschland ungerecht niedrig, gerecht oder ungerecht hoch?	☐	☐	☐	☐	☐	☐	☐

[1] Bezugsdatum: 2017.
