# Supplementary material for: Justice Evaluation of the Income Distribution (JEID): Development and validation of a short scale for the subjective assessment of objective differences in earnings
Source: PLoS One. 2023 Jan 26;18(1):e0281021. doi: 10.1371/journal.pone.0281021 (PMC9879472; doi:10.1371/journal.pone.0281021)
Supplement: S3 Appendix — (PDF) [file pone.0281021.s003.pdf]

S3 Appendix: Answer Sheet of the JEID Scale (English-Language Version, UK)

We would now like to ask you a few questions about income differences in the UK.

Current survey results show that low-income earners in the UK make **£1,100** on average per month. Middle-income earners make **£2,100** on average per month, upper-middle-income earners make **£3,300** on average per month, and high-income earners make **£6,000** on average per month. Top-income earners in the UK make more than **£8,500** per month.

These figures refer to the **gross monthly income** of full-time employees.[1] By gross income we mean the amount earned per month by someone who works **full-time** before deductions for taxes and social security contributions.

We would now like to ask you how fair you find these incomes.

	unfairly low			fair			unfairly high
**Low-income earners** such as **cleaners, shop salespeople, or couriers** make **£1,100** (gross) on average per month. This means that they earn <u>less</u> than **90%** of all employees in the UK.<br>Do you think that the income of low-income earners in the UK is unfairly low, fair, or unfairly high?	☐	☐	☐	☐	☐	☐	☐
**Middle-income earners** such as **nurses, office clerks, or social workers** make **£2,100** (gross) on average per month. This means that their income is in the <u>mid-range</u>.<br>Do you think that the income of middle-income earners in the UK is unfairly low, fair, or unfairly high?	☐	☐	☐	☐	☐	☐	☐
**Upper-middle-income earners** such as **teachers, police officers, or programmers** make **£3,300** (gross) on average per month. This means that they earn <u>more</u> than **80%** of all employees in the UK.<br>Do you think that the income of upper-middle-income earners in the UK is unfairly low, fair, or unfairly high?	☐	☐	☐	☐	☐	☐	☐
**High-income earners** such as **doctors, engineers, or department managers** make **£6,000** (gross) on average per month. This means that they earn <u>more</u> than **90%** of all employees in the UK.<br>Do you think that the income of high-income earners in the UK is unfairly low, fair, or unfairly high?	☐	☐	☐	☐	☐	☐	☐
**Top-income earners** such as **chief executives, bank directors, or management consultants** make more than **£8,500** (gross) per month. This means that they earn <u>more</u> than **99%** of all employees in the UK.<br>Do you think that the income of top-income earners in the UK is unfairly low, fair, or unfairly high?	☐	☐	☐	☐	☐	☐	☐

[1] Reference date: 2017/2018.
