# Supplementary material for: Justice Evaluation of the Income Distribution (JEID): Development and validation of a short scale for the subjective assessment of objective differences in earnings
Source: PLoS One. 2023 Jan 26;18(1):e0281021. doi: 10.1371/journal.pone.0281021 (PMC9879472; doi:10.1371/journal.pone.0281021)
Supplement: S4 Appendix — (PDF) [file pone.0281021.s004.pdf]

S4 Appendix: R Code for Analysis

```
####################
#Analysis of Study 1
####################

#Clear workspace (run if desired)
rm(list = ls())

# #List of project directories
# dirs <- list(
#   data = "...",
#   analysis = "...")

#Load required packages
if (!require(car)) {install.packages("car")}; library(car)
if (!require(psych)) {install.packages("psych")}; library(psych)
if (!require(data.table)) {install.packages("data.table")}; library(data.table)
if (!require(dplyr)) {install.packages("dplyr")}; library(dplyr)
if (!require(MplusAutomation)){install.packages("MplusAutomation")}; library(MplusAutomation)
if (!require(factoextra)) {install.packages("factoextra")}; library(factoextra)
if (!require(cluster)) {install.packages("cluster")}; library(cluster)
if (!require(ggplot2)) {install.packages("ggplot2")}; library(ggplot2)

#Load dataset
load(paste0(dirs$data, "JEID.rda"))

##########################################################################

#############################
#Step 0: Preparation of data
#############################

#Recode negative-worded items (and response scales)
JEID <- mutate(JEID,
        bsjne01r = 6-bsjne01,
        bsjeq02r = 6-bsjeq02,
        bsjea03r = 6-bsjea03,
        bsjen04r = 6-bsjen04,
        bsjne05r = 6-bsjne05,
        bsjen06r = 6-bsjen06,
        bsjea07r = 6-bsjea07,
        bsjeq08r = 6-bsjeq08,
        bsjeq09r = 6-bsjeq09,
        bsjne10r = 6-bsjne10,
        bsjea11r = 6-bsjea11,
```

```
		bsjen12r = 6-bsjen12,
		healthr = 6-health,
		ksenq04r = 6-ksenq04,
		ksenq05r = 6-ksenq05,
		ksenq06r = 6-ksenq06)

#Combine both conditions main survey
JEID <- mutate(JEID, jeid1 = coalesce(jedgv01a, jedgv01b))
JEID <- mutate(JEID, jeid2 = coalesce(jeddv02a, jeddv02b))
JEID <- mutate(JEID, jeid3 = coalesce(jedgu03a, jedgu03b))
JEID <- mutate(JEID, jeid4 = coalesce(jedbe04a, jedbe04b))
JEID <- mutate(JEID, jeid5 = coalesce(jedtp05a, jedtp05b))

#Combine both conditions retest
JEID <- mutate(JEID, jeid1r = coalesce(jedgv01ar, jedgv01br))
JEID <- mutate(JEID, jeid2r = coalesce(jeddv02ar, jeddv02br))
JEID <- mutate(JEID, jeid3r = coalesce(jedgu03ar, jedgu03br))
JEID <- mutate(JEID, jeid4r = coalesce(jedbe04ar, jedbe04br))
JEID <- mutate(JEID, jeid5r = coalesce(jedtp05ar, jedtp05br))

##########################################################################

##################################
#Step 1: Scale values and graphs
##################################

#Both conditions combined
JEID$jeid <- (JEID$jeid1+JEID$jeid2+JEID$jeid3+JEID$jeid4+JEID$jeid5)/5

describe(JEID$jeid1)
describe(JEID$jeid2)
describe(JEID$jeid3)
describe(JEID$jeid4)
describe(JEID$jeid5)
describe(JEID$jeid)

barplot(table(JEID$jeid1), xlab = "Answer categories", ylab = "Frequencies")
barplot(table(JEID$jeid2), xlab = "Answer categories", ylab = "Frequencies")
barplot(table(JEID$jeid3), xlab = "Answer categories", ylab = "Frequencies")
barplot(table(JEID$jeid4), xlab = "Answer categories", ylab = "Frequencies")
barplot(table(JEID$jeid5), xlab = "Answer categories", ylab = "Frequencies")
barplot(table(JEID$jeid), xlab = "Answer categories", ylab = "Frequencies")

#Without numerical label
JEID$jeida <- (JEID$jedgv01a+JEID$jeddv02a+JEID$jedgu03a+JEID$jedbe04a+JEID$jedtp05a)/5
```

```
describe(JEID$jedgv01a)
describe(JEID$jeddv02a)
describe(JEID$jedgu03a)
describe(JEID$jedbe04a)
describe(JEID$jedtp05a)
describe(JEID$jeida)

barplot(table(JEID$jedgv01a), xlab = "Answer categories", ylab = "Frequencies")
barplot(table(JEID$jeddv02a), xlab = "Answer categories", ylab = "Frequencies")
barplot(table(JEID$jedgu03a), xlab = "Answer categories", ylab = "Frequencies")
barplot(table(JEID$jedbe04a), xlab = "Answer categories", ylab = "Frequencies")
barplot(table(JEID$jedtp05a), xlab = "Answer categories", ylab = "Frequencies")
barplot(table(JEID$jeida), xlab = "Answer categories", ylab = "Frequencies")

#With numerical label
JEID$jeidb <- (JEID$jedgv01b+JEID$jeddv02b+JEID$jedgu03b+JEID$jedbe04b+JEID$jedtp05b)/5

describe(JEID$jedgv01b)
describe(JEID$jeddv02b)
describe(JEID$jedgu03b)
describe(JEID$jedbe04b)
describe(JEID$jedtp05b)
describe(JEID$jeidb)

barplot(table(JEID$jedgv01b), xlab = "Answer categories", ylab = "Frequencies")
barplot(table(JEID$jeddv02b), xlab = "Answer categories", ylab = "Frequencies")
barplot(table(JEID$jedgu03b), xlab = "Answer categories", ylab = "Frequencies")
barplot(table(JEID$jedbe04b), xlab = "Answer categories", ylab = "Frequencies")
barplot(table(JEID$jedtp05b), xlab = "Answer categories", ylab = "Frequencies")
barplot(table(JEID$jeidb), xlab = "Answer categories", ylab = "Frequencies")

################################################################################

############################
#Step 2: Group comparisons
############################

#t-tests
t.test(JEID$jedgv01a, JEID$jedgv01b, paired = FALSE)
t.test(JEID$jeddv02a, JEID$jeddv02b, paired = FALSE)
t.test(JEID$jedgu03a, JEID$jedgu03b, paired = FALSE)
t.test(JEID$jedbe04a, JEID$jedbe04b, paired = FALSE)
t.test(JEID$jedtp05a, JEID$jedtp05b, paired = FALSE)
t.test(JEID$jeida, JEID$jeidb, paired = FALSE)

################################################################################
```

```
####################
#Step 3: Reliability
####################

##Retest reliability

JEID$jeidr <- (JEID$jeid1r+JEID$jeid2r+JEID$jeid3r+JEID$jeid4r+JEID$jeid5r)/5

cor.test(JEID$jeid1, JEID$jeid1r, use = "pairwise.complete.obs")
cor.test(JEID$jeid2, JEID$jeid2r, use = "pairwise.complete.obs")
cor.test(JEID$jeid3, JEID$jeid3r, use = "pairwise.complete.obs")
cor.test(JEID$jeid4, JEID$jeid4r, use = "pairwise.complete.obs")
cor.test(JEID$jeid5, JEID$jeid5r, use = "pairwise.complete.obs")

######################################################################

##Individual profile correlation

#Reduce data to JEID items
JEID_only <- JEID %>% dplyr::select(jeid1:jeid5, jeid1r:jeid5r)
save(JEID_only, file = paste0(dirs$data, "JEID_only.Rda"))

#Create person ID
JEID_only <- cbind(ID = 1:nrow(JEID_only), JEID_only)
JEID_only

#Compute the correlation between two sets of items (1:5 and 6:10)
#for each person and append that "ipsative correlation" to the data
JEID_only_r <- JEID_only %>%
  group_by(ID) %>%
  mutate(ipscor = cor(x = c_across(jeid1:jeid5),
                      y = c_across(jeid1r:jeid5r))) %>%
  ungroup()
JEID_only_r

#Delete cases with missing values
JEID_only_r <- na.omit(JEID_only_r)
save(JEID_only_r, file = paste0(dirs$data, "JEID_only_r.Rda"))

#Summarize this correlation in the sample
JEID_only_r %>% summarize(mean_ipscor = mean(ipscor))

######################################################################

################################
```

```
#Step 4: Latent Profile Analysis
##################################

##Prepare dataset for LPAs in Mplus
prepareMplusData(df = JEID, filename = "JEID.dat.dat", inpfile = TRUE,
        keepCols = c("jeid1", "jeid2", "jeid3", "jeid4", "jeid5"))

##Sample Mplus code (3 profile solution)

#Step 1

# TITLE:          JEID - Latent profile analysis (3 categories)
#
# DATA:           FILE = "JEID_LPA.dat";
#
# VARIABLE:
# NAMES = jeid1-jeid5;
# CLASSES = c (3);
# MISSING = .;
#
# ANALYSIS:
# TYPE = MIXTURE;
# STARTS = 20000 5000;
# STITERATIONS = 50;

#Step 2

# TITLE:          JEID - Latent profile analysis (3 categories)
#
# DATA:           FILE = "JEID_LPA.dat";
#
# VARIABLE:
# NAMES = jeid1-jeid5;
# CLASSES = c (3);
# MISSING = .;
#
# ANALYSIS:
# TYPE = MIXTURE;
# STARTS = 0;
# OPTSEED = 274044;        !the best loglikelihood value found in Step 1
# LRTSTARTS =  0 0 1000 250;
#
# PLOT:
# TYPE = plot3;
# SERIES = jeid1 (1) jeid2 (2) jeid3 (3) jeid4 (4) jeid5 (5);
#
```

```
# OUTPUT:                  TECH11 TECH14;

###############################################################################

####################################
#Step 5: k-means cluster analysis
####################################

##Prepare data

#Reduce dataset to JEID-variables
JEID_reduced <- JEID %>% dplyr::select(jeid1:jeid5)

#Identifiy missing values
df <- na.omit(JEID_reduced)
JEID_reduced <- na.omit(JEID_reduced)

#Standardize the variables
df <- scale(df)
head(df, n = 20)

##Determine the optimal number of clusters

#Elbow method
fviz_nbclust(df, kmeans, method = "wss")

#Average silhouette method
fviz_nbclust(df, kmeans, method = "silhouette")

#Gap statistic method
gap_stat <- clusGap(df, FUN = kmeans, nstart = 25,
                    K.max = 10, B = 50)
print(gap_stat, method = "firstmax")
fviz_gap_stat(gap_stat)

##Extracting results

set.seed(123)

#k = 3
final <- kmeans(df, 3, nstart = 25)
print(final)

#Visualize the clusters
fviz_cluster(final, data = df)
```

```
#Add the point classifications to the original data
#and compute the mean of each variable by clusters
JEID_reduced %>%
  mutate(Cluster = final$cluster) %>%
  group_by(Cluster) %>%
  summarise_all("mean")
# Cluster jeid1 jeid2 jeid3 jeid4 jeid5
# <int> <dbl> <dbl> <dbl> <dbl> <dbl>
#    1  1.42  2.78  5.51  6.01  6.87
#    2  3.92  4.07  4.14  4.08  4.25
#    3  1.40  2.4   3.76  4.24  5.74

#Build new data frame
jeid_k3 <- data.frame(group = c("Inequality averse (28.6%)",
                                "Inequality averse (28.6%)",
                                "Inequality averse (28.6%)",
                                "Inequality averse (28.6%)",
                                "Inequality averse (28.6%)",
                                "Bottom-inequality averse (49.5%)",
                                "Bottom-inequality averse (49.5%)",
                                "Bottom-inequality averse (49.5%)",
                                "Bottom-inequality averse (49.5%)",
                                "Bottom-inequality averse (49.5%)",
                                "Status quo justification (21.9%)",
                                "Status quo justification (21.9%)",
                                "Status quo justification (21.9%)",
                                "Status quo justification (21.9%)",
                                "Status quo justification (21.9%)"),
                      income = c("P10", "P50", "P80", "P90", "P99",
                                 "P10", "P50", "P80", "P90", "P99",
                                 "P10", "P50", "P80", "P90", "P99"),
                      answer = c(1.42, 2.78, 5.51, 6.01, 6.87,
                                 1.40, 2.40, 3.76, 4.24, 5.74,
                                 3.92, 4.07, 4.14, 4.08, 4.25))
print(jeid_k3)

#Relevel group factor to change the order of the legend
jeid_k3$group <- factor(jeid_k3$group,
                        levels = c("Inequality averse (28.6%)",
                                   "Bottom-inequality averse (49.5%)",
                                   "Status quo justification (21.9%)"))

#Visualize the mean values per group
jeid_k3 %>%
  ggplot(aes(x = factor(income, level = c('P10', 'P50', 'P80', 'P90', 'P99')),
         y = answer,
```

```
        group = group, color = group)) +
  geom_line() + geom_point() + theme_classic() +
  xlab("Income percentile") + ylab("Answer on the rating scale") +
  geom_line(aes(color = group), size = 1) +
  scale_color_manual(values = c("blue", "red", "green")) +
  guides(color = guide_legend("Justice evaluation group"))

detach("package:dplyr", unload = TRUE)

################################################################################

##############################
#Step 6: Construct validity
##############################

##Value orientations

#Basic social justice orientation
describe(JEID$bsjne01)
JEID$need <- JEID$bsjne01r+JEID$bsjne05r+JEID$bsjne10r
JEID$equi <- JEID$bsjeq02r+JEID$bsjeq08r+JEID$bsjeq09r
JEID$equa <- JEID$bsjea03r+JEID$bsjea07r+JEID$bsjea11r
JEID$enti <- JEID$bsjen04r+JEID$bsjen06r+JEID$bsjen12r
describe(JEID$need)
describe(JEID$equi)
describe(JEID$equa)
describe(JEID$enti)

cor.test(JEID$jeid1, JEID$need, use = "pairwise.complete.obs")
cor.test(JEID$jeid2, JEID$need, use = "pairwise.complete.obs")
cor.test(JEID$jeid3, JEID$need, use = "pairwise.complete.obs")
cor.test(JEID$jeid4, JEID$need, use = "pairwise.complete.obs")
cor.test(JEID$jeid5, JEID$need, use = "pairwise.complete.obs")

cor.test(JEID$jeid1, JEID$equi, use = "pairwise.complete.obs")
cor.test(JEID$jeid2, JEID$equi, use = "pairwise.complete.obs")
cor.test(JEID$jeid3, JEID$equi, use = "pairwise.complete.obs")
cor.test(JEID$jeid4, JEID$equi, use = "pairwise.complete.obs")
cor.test(JEID$jeid5, JEID$equi, use = "pairwise.complete.obs")

cor.test(JEID$jeid1, JEID$equa, use = "pairwise.complete.obs")
cor.test(JEID$jeid2, JEID$equa, use = "pairwise.complete.obs")
cor.test(JEID$jeid3, JEID$equa, use = "pairwise.complete.obs")
cor.test(JEID$jeid4, JEID$equa, use = "pairwise.complete.obs")
cor.test(JEID$jeid5, JEID$equa, use = "pairwise.complete.obs")
```

```
cor.test(JEID$jeid1, JEID$enti, use = "pairwise.complete.obs")
cor.test(JEID$jeid2, JEID$enti, use = "pairwise.complete.obs")
cor.test(JEID$jeid3, JEID$enti, use = "pairwise.complete.obs")
cor.test(JEID$jeid4, JEID$enti, use = "pairwise.complete.obs")
cor.test(JEID$jeid5, JEID$enti, use = "pairwise.complete.obs")

#Left–right self-placement
describe(JEID$leri)
JEID$pleri <- recode(JEID$leri, "1 = 1; 2 = 2; 3 = 3; 4 = 4; 5 = 5; 6 = 6;
                     7 = 7; 8 = 8; 9 = 9; 10 = 10; 11 = NA")
describe(JEID$pleri)

cor.test(JEID$jeid1, JEID$pleri, use = "pairwise.complete.obs")
cor.test(JEID$jeid2, JEID$pleri, use = "pairwise.complete.obs")
cor.test(JEID$jeid3, JEID$pleri, use = "pairwise.complete.obs")
cor.test(JEID$jeid4, JEID$pleri, use = "pairwise.complete.obs")
cor.test(JEID$jeid5, JEID$pleri, use = "pairwise.complete.obs")

#Human values
describe(JEID$hvesd01)
JEID$hv01 <- recode(JEID$hvesd01, "1 = 6;  2 = 5; 3 = 4; 4 = 3; 5 = 2; 6 = 1; else = NA")
JEID$hv02 <- recode(JEID$hvesd02, "1 = 6;  2 = 5; 3 = 4; 4 = 3; 5 = 2; 6 = 1; else = NA")
JEID$hv03 <- recode(JEID$hvesd03, "1 = 6;  2 = 5; 3 = 4; 4 = 3; 5 = 2; 6 = 1; else = NA")
JEID$hv04 <- recode(JEID$hvesd04, "1 = 6;  2 = 5; 3 = 4; 4 = 3; 5 = 2; 6 = 1; else = NA")
JEID$hv05 <- recode(JEID$hvesd05, "1 = 6;  2 = 5; 3 = 4; 4 = 3; 5 = 2; 6 = 1; else = NA")
JEID$hv06 <- recode(JEID$hvesd06, "1 = 6;  2 = 5; 3 = 4; 4 = 3; 5 = 2; 6 = 1; else = NA")
JEID$hv07 <- recode(JEID$hvesd07, "1 = 6;  2 = 5; 3 = 4; 4 = 3; 5 = 2; 6 = 1; else = NA")
JEID$hv08 <- recode(JEID$hvesd08, "1 = 6;  2 = 5; 3 = 4; 4 = 3; 5 = 2; 6 = 1; else = NA")
JEID$hv09 <- recode(JEID$hvesd09, "1 = 6;  2 = 5; 3 = 4; 4 = 3; 5 = 2; 6 = 1; else = NA")
JEID$hv10 <- recode(JEID$hvesd10, "1 = 6;  2 = 5; 3 = 4; 4 = 3; 5 = 2; 6 = 1; else = NA")
JEID$hv11 <- recode(JEID$hvesd11, "1 = 6;  2 = 5; 3 = 4; 4 = 3; 5 = 2; 6 = 1; else = NA")
JEID$hv12 <- recode(JEID$hvesd12, "1 = 6;  2 = 5; 3 = 4; 4 = 3; 5 = 2; 6 = 1; else = NA")
JEID$hv13 <- recode(JEID$hvesd13, "1 = 6;  2 = 5; 3 = 4; 4 = 3; 5 = 2; 6 = 1; else = NA")
JEID$hv14 <- recode(JEID$hvesd14, "1 = 6;  2 = 5; 3 = 4; 4 = 3; 5 = 2; 6 = 1; else = NA")
JEID$hv15 <- recode(JEID$hvesd15, "1 = 6;  2 = 5; 3 = 4; 4 = 3; 5 = 2; 6 = 1; else = NA")
JEID$hv16 <- recode(JEID$hvesd16, "1 = 6;  2 = 5; 3 = 4; 4 = 3; 5 = 2; 6 = 1; else = NA")
JEID$hv17 <- recode(JEID$hvesd17, "1 = 6;  2 = 5; 3 = 4; 4 = 3; 5 = 2; 6 = 1; else = NA")
JEID$hv18 <- recode(JEID$hvesd18, "1 = 6;  2 = 5; 3 = 4; 4 = 3; 5 = 2; 6 = 1; else = NA")
JEID$hv19 <- recode(JEID$hvesd19, "1 = 6;  2 = 5; 3 = 4; 4 = 3; 5 = 2; 6 = 1; else = NA")
JEID$hv20 <- recode(JEID$hvesd20, "1 = 6;  2 = 5; 3 = 4; 4 = 3; 5 = 2; 6 = 1; else = NA")
JEID$hv21 <- recode(JEID$hvesd21, "1 = 6;  2 = 5; 3 = 4; 4 = 3; 5 = 2; 6 = 1; else = NA")

JEID$selfd <- (JEID$hv01+JEID$hv11)/2
JEID$power <- (JEID$hv02+JEID$hv17)/2
JEID$unive <- (JEID$hv03+JEID$hv08+JEID$hv19)/3
JEID$achie <- (JEID$hv04+JEID$hv13)/2
```

```
JEID$secur <- (JEID$hv05+JEID$hv14)/2
JEID$stimu <- (JEID$hv06+JEID$hv15)/2
JEID$confo <- (JEID$hv07+JEID$hv16)/2
JEID$tradi <- (JEID$hv09+JEID$hv20)/2
JEID$hedon <- (JEID$hv10+JEID$hv21)/2
JEID$benev <- (JEID$hv12+JEID$hv18)/2

describe(JEID$selfd)
describe(JEID$power)
describe(JEID$unive)
describe(JEID$achie)
describe(JEID$secur)
describe(JEID$stimu)
describe(JEID$confo)
describe(JEID$tradi)
describe(JEID$hedon)
describe(JEID$benev)

cor.test(JEID$jeid1, JEID$selfd, use = "pairwise.complete.obs")
cor.test(JEID$jeid2, JEID$selfd, use = "pairwise.complete.obs")
cor.test(JEID$jeid3, JEID$selfd, use = "pairwise.complete.obs")
cor.test(JEID$jeid4, JEID$selfd, use = "pairwise.complete.obs")
cor.test(JEID$jeid5, JEID$selfd, use = "pairwise.complete.obs")

cor.test(JEID$jeid1, JEID$power, use = "pairwise.complete.obs")
cor.test(JEID$jeid2, JEID$power, use = "pairwise.complete.obs")
cor.test(JEID$jeid3, JEID$power, use = "pairwise.complete.obs")
cor.test(JEID$jeid4, JEID$power, use = "pairwise.complete.obs")
cor.test(JEID$jeid5, JEID$power, use = "pairwise.complete.obs")

cor.test(JEID$jeid1, JEID$unive, use = "pairwise.complete.obs")
cor.test(JEID$jeid2, JEID$unive, use = "pairwise.complete.obs")
cor.test(JEID$jeid3, JEID$unive, use = "pairwise.complete.obs")
cor.test(JEID$jeid4, JEID$unive, use = "pairwise.complete.obs")
cor.test(JEID$jeid5, JEID$unive, use = "pairwise.complete.obs")

cor.test(JEID$jeid1, JEID$achie, use = "pairwise.complete.obs")
cor.test(JEID$jeid2, JEID$achie, use = "pairwise.complete.obs")
cor.test(JEID$jeid3, JEID$achie, use = "pairwise.complete.obs")
cor.test(JEID$jeid4, JEID$achie, use = "pairwise.complete.obs")
cor.test(JEID$jeid5, JEID$achie, use = "pairwise.complete.obs")

cor.test(JEID$jeid1, JEID$secur, use = "pairwise.complete.obs")
cor.test(JEID$jeid2, JEID$secur, use = "pairwise.complete.obs")
cor.test(JEID$jeid3, JEID$secur, use = "pairwise.complete.obs")
cor.test(JEID$jeid4, JEID$secur, use = "pairwise.complete.obs")
```

```
cor.test(JEID$jeid5, JEID$secur, use = "pairwise.complete.obs")

cor.test(JEID$jeid1, JEID$stimu, use = "pairwise.complete.obs")
cor.test(JEID$jeid2, JEID$stimu, use = "pairwise.complete.obs")
cor.test(JEID$jeid3, JEID$stimu, use = "pairwise.complete.obs")
cor.test(JEID$jeid4, JEID$stimu, use = "pairwise.complete.obs")
cor.test(JEID$jeid5, JEID$stimu, use = "pairwise.complete.obs")

cor.test(JEID$jeid1, JEID$confo, use = "pairwise.complete.obs")
cor.test(JEID$jeid2, JEID$confo, use = "pairwise.complete.obs")
cor.test(JEID$jeid3, JEID$confo, use = "pairwise.complete.obs")
cor.test(JEID$jeid4, JEID$confo, use = "pairwise.complete.obs")
cor.test(JEID$jeid5, JEID$confo, use = "pairwise.complete.obs")

cor.test(JEID$jeid1, JEID$tradi, use = "pairwise.complete.obs")
cor.test(JEID$jeid2, JEID$tradi, use = "pairwise.complete.obs")
cor.test(JEID$jeid3, JEID$tradi, use = "pairwise.complete.obs")
cor.test(JEID$jeid4, JEID$tradi, use = "pairwise.complete.obs")
cor.test(JEID$jeid5, JEID$tradi, use = "pairwise.complete.obs")

cor.test(JEID$jeid1, JEID$hedon, use = "pairwise.complete.obs")
cor.test(JEID$jeid2, JEID$hedon, use = "pairwise.complete.obs")
cor.test(JEID$jeid3, JEID$hedon, use = "pairwise.complete.obs")
cor.test(JEID$jeid4, JEID$hedon, use = "pairwise.complete.obs")
cor.test(JEID$jeid5, JEID$hedon, use = "pairwise.complete.obs")

cor.test(JEID$jeid1, JEID$benev, use = "pairwise.complete.obs")
cor.test(JEID$jeid2, JEID$benev, use = "pairwise.complete.obs")
cor.test(JEID$jeid3, JEID$benev, use = "pairwise.complete.obs")
cor.test(JEID$jeid4, JEID$benev, use = "pairwise.complete.obs")
cor.test(JEID$jeid5, JEID$benev, use = "pairwise.complete.obs")

##Sociodemographic characteristics

#Gender (1 = male, 2 = female)
describe(JEID$sex)
cor.test(JEID$jeid1, JEID$sex, use = "pairwise.complete.obs")
cor.test(JEID$jeid2, JEID$sex, use = "pairwise.complete.obs")
cor.test(JEID$jeid3, JEID$sex, use = "pairwise.complete.obs")
cor.test(JEID$jeid4, JEID$sex, use = "pairwise.complete.obs")
cor.test(JEID$jeid5, JEID$sex, use = "pairwise.complete.obs")

#Age
describe(JEID$age)
cor.test(JEID$jeid1, JEID$age, use = "pairwise.complete.obs")
cor.test(JEID$jeid2, JEID$age, use = "pairwise.complete.obs")
```

```
cor.test(JEID$jeid3, JEID$age, use = "pairwise.complete.obs")
cor.test(JEID$jeid4, JEID$age, use = "pairwise.complete.obs")
cor.test(JEID$jeid5, JEID$age, use = "pairwise.complete.obs")

#Educational attainment
describe(JEID$edu)
cor.test(JEID$jeid1, JEID$edu, use = "pairwise.complete.obs")
cor.test(JEID$jeid2, JEID$edu, use = "pairwise.complete.obs")
cor.test(JEID$jeid3, JEID$edu, use = "pairwise.complete.obs")
cor.test(JEID$jeid4, JEID$edu, use = "pairwise.complete.obs")
cor.test(JEID$jeid5, JEID$edu, use = "pairwise.complete.obs")

#Gross income
describe(JEID$inco02tb)
JEID$inco02 <- recode(JEID$inco02tb, "-99 = NA; else = JEID$inco02tb")
describe(JEID$inco02)

cor.test(JEID$jeid1, JEID$inco02, use = "pairwise.complete.obs")
cor.test(JEID$jeid2, JEID$inco02, use = "pairwise.complete.obs")
cor.test(JEID$jeid3, JEID$inco02, use = "pairwise.complete.obs")
cor.test(JEID$jeid4, JEID$inco02, use = "pairwise.complete.obs")
cor.test(JEID$jeid5, JEID$inco02, use = "pairwise.complete.obs")

##Justice consequences

#General life satisfaction
describe(JEID$gls)
cor.test(JEID$jeid1, JEID$gls, use = "pairwise.complete.obs")
cor.test(JEID$jeid2, JEID$gls, use = "pairwise.complete.obs")
cor.test(JEID$jeid3, JEID$gls, use = "pairwise.complete.obs")
cor.test(JEID$jeid4, JEID$gls, use = "pairwise.complete.obs")
cor.test(JEID$jeid5, JEID$gls, use = "pairwise.complete.obs")

#General health
describe(JEID$healthr)
cor.test(JEID$jeid1, JEID$healthr, use = "pairwise.complete.obs")
cor.test(JEID$jeid2, JEID$healthr, use = "pairwise.complete.obs")
cor.test(JEID$jeid3, JEID$healthr, use = "pairwise.complete.obs")
cor.test(JEID$jeid4, JEID$healthr, use = "pairwise.complete.obs")
cor.test(JEID$jeid5, JEID$healthr, use = "pairwise.complete.obs")

#Trust in government
describe(JEID$poltr)
JEID$poltrust <- recode(JEID$poltr, "1 = 1; 2 = 2; 3 = 3; 4 = 4; 5 = 5; 6 = 6;
                        7 = 7; 8 = 8; 9 = 9; 10 = 10; 11 = 11;
                        else = NA")
```

```
describe(JEID$poltrust)

cor.test(JEID$jeid1, JEID$poltrust, use = "pairwise.complete.obs")
cor.test(JEID$jeid2, JEID$poltrust, use = "pairwise.complete.obs")
cor.test(JEID$jeid3, JEID$poltrust, use = "pairwise.complete.obs")
cor.test(JEID$jeid4, JEID$poltrust, use = "pairwise.complete.obs")
cor.test(JEID$jeid5, JEID$poltrust, use = "pairwise.complete.obs")

##Social desirability

describe(JEID$ksepq01)
JEID$SDPQ <- JEID$ksepq01+JEID$ksepq02+JEID$ksepq03
JEID$SDNQ <- JEID$ksenq04r+JEID$ksenq05r+JEID$ksenq06r
describe(JEID$SDPQ)
describe(JEID$SDNQ)

cor.test(JEID$jeid1, JEID$SDPQ, use = "pairwise.complete.obs")
cor.test(JEID$jeid2, JEID$SDPQ, use = "pairwise.complete.obs")
cor.test(JEID$jeid3, JEID$SDPQ, use = "pairwise.complete.obs")
cor.test(JEID$jeid4, JEID$SDPQ, use = "pairwise.complete.obs")
cor.test(JEID$jeid5, JEID$SDPQ, use = "pairwise.complete.obs")

cor.test(JEID$jeid1, JEID$SDNQ, use = "pairwise.complete.obs")
cor.test(JEID$jeid2, JEID$SDNQ, use = "pairwise.complete.obs")
cor.test(JEID$jeid3, JEID$SDNQ, use = "pairwise.complete.obs")
cor.test(JEID$jeid4, JEID$SDNQ, use = "pairwise.complete.obs")
cor.test(JEID$jeid5, JEID$SDNQ, use = "pairwise.complete.obs")

################################################################################

###################################
#Step 7: Descriptive statistics
###################################

#Age
summary(JEID$age)
sd(JEID$age)

#Proportion of women
table(JEID$sex)

#Educational level
table(JEID$quota)
#Quote 1: male, lower education, 18-29
#Quote 2: male, lower education, 30-49
#Quote 3: male, lower education, 50-69
```

```
#Quote 4: male, middle education, 18-29
#Quote 5: male, middle education, 30-49
#Quote 6: male, middle education, 50-69
#Quote 7: male, upper education, 18-29
#Quote 8: male, upper education, 30-49
#Quote 9: male, upper education, 50-69
#Quote 10: female, lower education, 18-29
#Quote 11: female, lower education, 30-49
#Quote 12: female, lower education, 50-69
#Quote 13: female, middle education, 18-29
#Quote 14: female, middle education, 30-49
#Quote 15: female, middle education, 50-69
#Quote 16: female, upper education, 18-29
#Quote 17: female, upper education, 30-49
#Quote 18: female, upper education, 50-69
edu1 <- subset(JEID, quota == 1 | quota == 2 | quota == 3 | quota == 10 |
        quota == 11 | quota == 12)
edu2 <- subset(JEID, quota == 4 | quota == 5 | quota == 6 | quota == 13 |
        quota == 14 | quota == 15)
edu3 <- subset(JEID, quota == 7 | quota == 8 | quota == 9 | quota == 16 |
        quota == 17 | quota == 18)

describe(edu1$quota)
describe(edu2$quota)
describe(edu3$quota)

################################################################################
```

```
#####################
#Analysis of Study 2
#####################

#Clear workspace (run if desired)
rm(list = ls())

# #List of project directories
# dirs <- list(
#   data = "...",
#   analysis = "...")

#Load required packages
if (!require(car)) {install.packages("car")}; library(car)
if (!require(psych)) {install.packages("psych")}; library(psych)
if (!require(data.table)) {install.packages("data.table")}; library(data.table)
if (!require(dplyr)) {install.packages("dplyr")}; library(dplyr)
if (!require(MplusAutomation)){install.packages("MplusAutomation")}; library(MplusAutomation)
library(foreign)
if (!require(factoextra)) {install.packages("factoextra")}; library(factoextra)
if (!require(cluster)) {install.packages("cluster")}; library(cluster)
if (!require(ggplot2)) {install.packages("ggplot2")}; library(ggplot2)

#Load dataset
load(paste0(dirs$data, "JEID_2.rda"))

##########################################################################

#############################
#Step 0: Preparation of data
#############################

#Recode negative-worded items (and response scales)
JEID_2 <- mutate(JEID_2,
                 bsjne01r = 6-bsjne01,
                 bsjeq02r = 6-bsjeq02,
                 bsjea03r = 6-bsjea03,
                 bsjen04r = 6-bsjen04,
                 bsjne05r = 6-bsjne05,
                 bsjen06r = 6-bsjen06,
                 bsjea07r = 6-bsjea07,
                 bsjeq08r = 6-bsjeq08,
                 bsjeq09r = 6-bsjeq09,
                 bsjne10r = 6-bsjne10,
                 bsjea11r = 6-bsjea11,
                 bsjen12r = 6-bsjen12,
```

```
		healthr = 6-health,
		ksenq04r = 6-ksenq04,
		ksenq05r = 6-ksenq05,
		ksenq06r = 6-ksenq06)

#Build mean scale values
JEID_2 <- mutate(JEID_2, jeid = (jeid01+jeid02+jeid03+jeid04+jeid05)/5)
JEID_2 <- mutate(JEID_2, jeidr = (jeidr01+jeidr02+jeidr03+jeidr04+jeidr05)/5)

#Split samples
JEID_without <- subset(JEID_2, JEIDform == 2)
JEID_with <- subset(JEID_2, JEIDform == 1)

###############################################################################

###################################
#Step 1: Scale values and graphs
###################################

#Both conditions combined
describe(JEID_2$jeid01)
describe(JEID_2$jeid02)
describe(JEID_2$jeid03)
describe(JEID_2$jeid04)
describe(JEID_2$jeid05)
describe(JEID_2$jeid)

barplot(table(JEID_2$jeid01), xlab = "Answer categories", ylab = "Frequencies")
barplot(table(JEID_2$jeid02), xlab = "Answer categories", ylab = "Frequencies")
barplot(table(JEID_2$jeid03), xlab = "Answer categories", ylab = "Frequencies")
barplot(table(JEID_2$jeid04), xlab = "Answer categories", ylab = "Frequencies")
barplot(table(JEID_2$jeid05), xlab = "Answer categories", ylab = "Frequencies")
barplot(table(JEID_2$jeid), xlab = "Answer categories", ylab = "Frequencies")

#Without numerical label
describe(JEID_without$jeid01)
describe(JEID_without$jeid02)
describe(JEID_without$jeid03)
describe(JEID_without$jeid04)
describe(JEID_without$jeid05)
describe(JEID_without$jeid)

barplot(table(JEID_without$jeid01), xlab = "Answer categories", ylab = "Frequencies")
barplot(table(JEID_without$jeid02), xlab = "Answer categories", ylab = "Frequencies")
barplot(table(JEID_without$jeid03), xlab = "Answer categories", ylab = "Frequencies")
barplot(table(JEID_without$jeid04), xlab = "Answer categories", ylab = "Frequencies")
```

```
barplot(table(JEID_without$jeid05), xlab = "Answer categories", ylab = "Frequencies")
barplot(table(JEID_without$jeid), xlab = "Answer categories", ylab = "Frequencies")

#With numerical label
describe(JEID_with$jeid01)
describe(JEID_with$jeid02)
describe(JEID_with$jeid03)
describe(JEID_with$jeid04)
describe(JEID_with$jeid05)
describe(JEID_with$jeid)

barplot(table(JEID_with$jeid01), xlab = "Answer categories", ylab = "Frequencies")
barplot(table(JEID_with$jeid02), xlab = "Answer categories", ylab = "Frequencies")
barplot(table(JEID_with$jeid03), xlab = "Answer categories", ylab = "Frequencies")
barplot(table(JEID_with$jeid04), xlab = "Answer categories", ylab = "Frequencies")
barplot(table(JEID_with$jeid05), xlab = "Answer categories", ylab = "Frequencies")
barplot(table(JEID_with$jeid), xlab = "Answer categories", ylab = "Frequencies")

##########################################################################

###########################
#Step 2: Group comparisons
###########################

#t-tests
t.test(JEID_without$jeid01, JEID_with$jeid01, paired = FALSE)
t.test(JEID_without$jeid02, JEID_with$jeid02, paired = FALSE)
t.test(JEID_without$jeid03, JEID_with$jeid03, paired = FALSE)
t.test(JEID_without$jeid04, JEID_with$jeid04, paired = FALSE)
t.test(JEID_without$jeid05, JEID_with$jeid05, paired = FALSE)
t.test(JEID_without$jeid, JEID_with$jeid, paired = FALSE)

##########################################################################

#####################
#Step 3: Reliability
#####################

##Retest reliability

cor.test(JEID_2$jeid01, JEID_2$jeidr01, use = "pairwise.complete.obs")
cor.test(JEID_2$jeid02, JEID_2$jeidr02, use = "pairwise.complete.obs")
cor.test(JEID_2$jeid03, JEID_2$jeidr03, use = "pairwise.complete.obs")
cor.test(JEID_2$jeid04, JEID_2$jeidr04, use = "pairwise.complete.obs")
cor.test(JEID_2$jeid05, JEID_2$jeidr05, use = "pairwise.complete.obs")
```

```
############################################################################

##Individual profile correlation

#Reduce data to JEID items
JEID_2_only <- JEID_2 %>% dplyr::select(lfdn, jeid01:jeid05, jeidr01:jeidr05)
save(JEID_2_only, file = paste0(dirs$data, "JEID_2_only.Rda"))

#Compute the correlation between two sets of items (1:5 and 6:10)
#for each person and append that "ipsative correlation" to the data
JEID_2_only_r <- JEID_2_only %>%
  group_by(lfdn) %>%
  mutate(ipscor = cor(x = c_across(jeid01:jeid05),
                    y = c_across(jeidr01:jeidr05))) %>%
  ungroup()
JEID_2_only_r

#Delete cases with missing values
JEID_2_only_r <- na.omit(JEID_2_only_r)
save(JEID_2_only_r, file = paste0(dirs$data, "JEID_2_only_r.Rda"))

#Summarize this correlation in the sample
JEID_2_only_r %>% summarize(mean_ipscor = mean(ipscor))

############################################################################

##################################
#Step 4: Latent Profile Analysis
##################################

##Prepare dataset for LPAs in Mplus
prepareMplusData(df = JEID_2, filename = "JEID_2.dat", inpfile = TRUE,
             keepCols = c("jeid01", "jeid02", "jeid03", "jeid04", "jeid05"))

##Sample Mplus code (3 profile solution)

#Step 1

# TITLE:              JEID - Latent profile analysis (3 categories)
#
# DATA:               FILE = "JEID_2.dat";
#
# VARIABLE:
# NAMES = jeid01-jeid05;
# CLASSES = c (3);
# MISSING = .;
```

```
#
# ANALYSIS:
# TYPE = MIXTURE;
# STARTS = 20000 5000;
# STITERATIONS = 50;

#Step 2

# TITLE:          JEID - Latent profile analysis (3 categories)
#
# DATA:           FILE = "JEID_2.dat";
#
# VARIABLE:
# NAMES = jeid01-jeid05;
# CLASSES = c (3);
# MISSING = .;
#
# ANALYSIS:
# TYPE = MIXTURE;
# STARTS = 0;
# OPTSEED = 357229;       !the best loglikelihood value found in Step 1
# LRTSTARTS =  0 0 1000 250;
#
# PLOT:
# TYPE = plot3;
# SERIES = jeid01 (1) jeid02 (2) jeid03 (3) jeid04 (4) jeid05 (5);
#
# OUTPUT:         TECH11 TECH14;

################################################################################

###################################
#Step 5: k-means cluster analysis
###################################

##Prepare data

#Reduce dataset to JEID-variables
JEID_reduced <- JEID_2 %>% dplyr::select(jeid01:jeid05)

#Identifiy missing values
df <- na.omit(JEID_reduced)

#Standardize the variables
df <- scale(df)
head(df, n = 20)
```

```
##Determine the optimal number of clusters

#Elbow method
fviz_nbclust(df, kmeans, method = "wss")

#Average silhouette method
fviz_nbclust(df, kmeans, method = "silhouette")

#Gap statistic method
gap_stat <- clusGap(df, FUN = kmeans, nstart = 25,
                    K.max = 10, B = 50)
print(gap_stat, method = "firstmax")
fviz_gap_stat(gap_stat)

##Extracting results

set.seed(123)

#k = 3
final <- kmeans(df, 3, nstart = 25)
print(final)

#Visualize the clusters
fviz_cluster(final, data = df)

#Add the point classifications to the original data
#and compute the mean of each variable by clusters
JEID_reduced %>%
  mutate(Cluster = final$cluster) %>%
  group_by(Cluster) %>%
  summarise_all("mean")
# Cluster jeid01 jeid02 jeid03 jeid04 jeid05
# <int>  <dbl>  <dbl>  <dbl>  <dbl>  <dbl>
#    1   1.74   3.57   7.02   7.46   8.66
#    2   1.58   2.54   4.51   5.03   7.33
#    3   4.69   5.17   5.26   5.24   5.49

#Build new data frame
jeid_k3 <- data.frame(group = c("Inequality averse (27.7%)",
                                "Inequality averse (27.7%)",
                                "Inequality averse (27.7%)",
                                "Inequality averse (27.7%)",
                                "Inequality averse (27.7%)",
                                "Bottom-inequality averse (43.2%)",
                                "Bottom-inequality averse (43.2%)",
```

```
                    "Bottom-inequality averse (43.2%)",
                    "Bottom-inequality averse (43.2%)",
                    "Bottom-inequality averse (43.2%)",
                    "Status quo justification (29.1%)",
                    "Status quo justification (29.1%)",
                    "Status quo justification (29.1%)",
                    "Status quo justification (29.1%)",
                    "Status quo justification (29.1%)"),
                income = c("P10", "P50", "P80", "P90", "P99",
                    "P10", "P50", "P80", "P90", "P99",
                    "P10", "P50", "P80", "P90", "P99"),
                answer = c(1.74, 3.57, 7.02, 7.46, 8.66,
                    1.58, 2.54, 4.51, 5.03, 7.33,
                    4.69, 5.17, 5.26, 5.24, 5.49))
print(jeid_k3)

#Relevel group factor to change the order of the legend
jeid_k3$group <- factor(jeid_k3$group,
                levels = c("Inequality averse (27.7%)",
                    "Bottom-inequality averse (43.2%)",
                    "Status quo justification (29.1%)"))

#Visualize the mean values per group
jeid_k3 %>%
  ggplot(aes(x = factor(income, level = c('P10', 'P50', 'P80', 'P90', 'P99')),
        y = answer,
        group = group, color = group)) +
  geom_line() + geom_point() + theme_classic() +
  xlab("Income percentile") + ylab("Answer on the rating scale") +
  geom_line(aes(color = group), size = 1) +
  scale_color_manual(values = c("blue", "red", "green")) +
  guides(color = guide_legend("Justice evaluation group"))

detach("package:dplyr", unload = TRUE)

##############################################################################

############################
#Step 6: Construct validity
############################

##Value orientations

#Basic social justice orientation
describe(JEID_2$bsjne01)
JEID_2$need <- JEID_2$bsjne01r+JEID_2$bsjne05r+JEID_2$bsjne10r
```

```
JEID_2$equi <- JEID_2$bsjeq02r+JEID_2$bsjeq08r+JEID_2$bsjeq09r
JEID_2$equa <- JEID_2$bsjea03r+JEID_2$bsjea07r+JEID_2$bsjea11r
JEID_2$enti <- JEID_2$bsjen04r+JEID_2$bsjen06r+JEID_2$bsjen12r
describe(JEID_2$need)
describe(JEID_2$equi)
describe(JEID_2$equa)
describe(JEID_2$enti)

cor.test(JEID_2$jeid01, JEID_2$need, use = "pairwise.complete.obs")
cor.test(JEID_2$jeid02, JEID_2$need, use = "pairwise.complete.obs")
cor.test(JEID_2$jeid03, JEID_2$need, use = "pairwise.complete.obs")
cor.test(JEID_2$jeid04, JEID_2$need, use = "pairwise.complete.obs")
cor.test(JEID_2$jeid05, JEID_2$need, use = "pairwise.complete.obs")

cor.test(JEID_2$jeid01, JEID_2$equi, use = "pairwise.complete.obs")
cor.test(JEID_2$jeid02, JEID_2$equi, use = "pairwise.complete.obs")
cor.test(JEID_2$jeid03, JEID_2$equi, use = "pairwise.complete.obs")
cor.test(JEID_2$jeid04, JEID_2$equi, use = "pairwise.complete.obs")
cor.test(JEID_2$jeid05, JEID_2$equi, use = "pairwise.complete.obs")

cor.test(JEID_2$jeid01, JEID_2$equa, use = "pairwise.complete.obs")
cor.test(JEID_2$jeid02, JEID_2$equa, use = "pairwise.complete.obs")
cor.test(JEID_2$jeid03, JEID_2$equa, use = "pairwise.complete.obs")
cor.test(JEID_2$jeid04, JEID_2$equa, use = "pairwise.complete.obs")
cor.test(JEID_2$jeid05, JEID_2$equa, use = "pairwise.complete.obs")

cor.test(JEID_2$jeid01, JEID_2$enti, use = "pairwise.complete.obs")
cor.test(JEID_2$jeid02, JEID_2$enti, use = "pairwise.complete.obs")
cor.test(JEID_2$jeid03, JEID_2$enti, use = "pairwise.complete.obs")
cor.test(JEID_2$jeid04, JEID_2$enti, use = "pairwise.complete.obs")
cor.test(JEID_2$jeid05, JEID_2$enti, use = "pairwise.complete.obs")

#Left–right self-placement
describe(JEID_2$leri)
JEID_2$pleri <- recode(JEID_2$leri, "1 = 1; 2 = 2; 3 = 3; 4 = 4; 5 = 5; 6 = 6;
                          7 = 7; 8 = 8; 9 = 9; 10 = 10; 11 = NA")
describe(JEID_2$pleri)

cor.test(JEID_2$jeid01, JEID_2$pleri, use = "pairwise.complete.obs")
cor.test(JEID_2$jeid02, JEID_2$pleri, use = "pairwise.complete.obs")
cor.test(JEID_2$jeid03, JEID_2$pleri, use = "pairwise.complete.obs")
cor.test(JEID_2$jeid04, JEID_2$pleri, use = "pairwise.complete.obs")
cor.test(JEID_2$jeid05, JEID_2$pleri, use = "pairwise.complete.obs")

#Human values
describe(JEID_2$hvesd01)
```

```
JEID_2$hv01 <- recode(JEID_2$hvesd01, "1 = 6;  2 = 5; 3 = 4; 4 = 3; 5 = 2; 6 = 1; else = NA")
JEID_2$hv02 <- recode(JEID_2$hvepo02, "1 = 6;  2 = 5; 3 = 4; 4 = 3; 5 = 2; 6 = 1; else = NA")
JEID_2$hv03 <- recode(JEID_2$hveun03, "1 = 6;  2 = 5; 3 = 4; 4 = 3; 5 = 2; 6 = 1; else = NA")
JEID_2$hv04 <- recode(JEID_2$hveac04, "1 = 6;  2 = 5; 3 = 4; 4 = 3; 5 = 2; 6 = 1; else = NA")
JEID_2$hv05 <- recode(JEID_2$hvesc05, "1 = 6;  2 = 5; 3 = 4; 4 = 3; 5 = 2; 6 = 1; else = NA")
JEID_2$hv06 <- recode(JEID_2$hvest06, "1 = 6;  2 = 5; 3 = 4; 4 = 3; 5 = 2; 6 = 1; else = NA")
JEID_2$hv07 <- recode(JEID_2$hveco07, "1 = 6;  2 = 5; 3 = 4; 4 = 3; 5 = 2; 6 = 1; else = NA")
JEID_2$hv08 <- recode(JEID_2$hveun08, "1 = 6;  2 = 5; 3 = 4; 4 = 3; 5 = 2; 6 = 1; else = NA")
JEID_2$hv09 <- recode(JEID_2$hvetr09, "1 = 6;  2 = 5; 3 = 4; 4 = 3; 5 = 2; 6 = 1; else = NA")
JEID_2$hv10 <- recode(JEID_2$hvehe10, "1 = 6;  2 = 5; 3 = 4; 4 = 3; 5 = 2; 6 = 1; else = NA")
JEID_2$hv11 <- recode(JEID_2$hvesd11, "1 = 6;  2 = 5; 3 = 4; 4 = 3; 5 = 2; 6 = 1; else = NA")
JEID_2$hv12 <- recode(JEID_2$hvebe12, "1 = 6;  2 = 5; 3 = 4; 4 = 3; 5 = 2; 6 = 1; else = NA")
JEID_2$hv13 <- recode(JEID_2$hveac13, "1 = 6;  2 = 5; 3 = 4; 4 = 3; 5 = 2; 6 = 1; else = NA")
JEID_2$hv14 <- recode(JEID_2$hvesc14, "1 = 6;  2 = 5; 3 = 4; 4 = 3; 5 = 2; 6 = 1; else = NA")
JEID_2$hv15 <- recode(JEID_2$hvest15, "1 = 6;  2 = 5; 3 = 4; 4 = 3; 5 = 2; 6 = 1; else = NA")
JEID_2$hv16 <- recode(JEID_2$hveco16, "1 = 6;  2 = 5; 3 = 4; 4 = 3; 5 = 2; 6 = 1; else = NA")
JEID_2$hv17 <- recode(JEID_2$hvepo17, "1 = 6;  2 = 5; 3 = 4; 4 = 3; 5 = 2; 6 = 1; else = NA")
JEID_2$hv18 <- recode(JEID_2$hvebe18, "1 = 6;  2 = 5; 3 = 4; 4 = 3; 5 = 2; 6 = 1; else = NA")
JEID_2$hv19 <- recode(JEID_2$hveun19, "1 = 6;  2 = 5; 3 = 4; 4 = 3; 5 = 2; 6 = 1; else = NA")
JEID_2$hv20 <- recode(JEID_2$hvetr20, "1 = 6;  2 = 5; 3 = 4; 4 = 3; 5 = 2; 6 = 1; else = NA")
JEID_2$hv21 <- recode(JEID_2$hvehe21, "1 = 6;  2 = 5; 3 = 4; 4 = 3; 5 = 2; 6 = 1; else = NA")

JEID_2$selfd <- (JEID_2$hv01+JEID_2$hv11)/2
JEID_2$power <- (JEID_2$hv02+JEID_2$hv17)/2
JEID_2$unive <- (JEID_2$hv03+JEID_2$hv08+JEID_2$hv19)/3
JEID_2$achie <- (JEID_2$hv04+JEID_2$hv13)/2
JEID_2$secur <- (JEID_2$hv05+JEID_2$hv14)/2
JEID_2$stimu <- (JEID_2$hv06+JEID_2$hv15)/2
JEID_2$confo <- (JEID_2$hv07+JEID_2$hv16)/2
JEID_2$tradi <- (JEID_2$hv09+JEID_2$hv20)/2
JEID_2$hedon <- (JEID_2$hv10+JEID_2$hv21)/2
JEID_2$benev <- (JEID_2$hv12+JEID_2$hv18)/2

describe(JEID_2$selfd)
describe(JEID_2$power)
describe(JEID_2$unive)
describe(JEID_2$achie)
describe(JEID_2$secur)
describe(JEID_2$stimu)
describe(JEID_2$confo)
describe(JEID_2$tradi)
describe(JEID_2$hedon)
describe(JEID_2$benev)

cor.test(JEID_2$jeid01, JEID_2$selfd, use = "pairwise.complete.obs")
cor.test(JEID_2$jeid02, JEID_2$selfd, use = "pairwise.complete.obs")
```

```
cor.test(JEID_2$jeid03, JEID_2$selfd, use = "pairwise.complete.obs")
cor.test(JEID_2$jeid04, JEID_2$selfd, use = "pairwise.complete.obs")
cor.test(JEID_2$jeid05, JEID_2$selfd, use = "pairwise.complete.obs")

cor.test(JEID_2$jeid01, JEID_2$power, use = "pairwise.complete.obs")
cor.test(JEID_2$jeid02, JEID_2$power, use = "pairwise.complete.obs")
cor.test(JEID_2$jeid03, JEID_2$power, use = "pairwise.complete.obs")
cor.test(JEID_2$jeid04, JEID_2$power, use = "pairwise.complete.obs")
cor.test(JEID_2$jeid05, JEID_2$power, use = "pairwise.complete.obs")

cor.test(JEID_2$jeid01, JEID_2$unive, use = "pairwise.complete.obs")
cor.test(JEID_2$jeid02, JEID_2$unive, use = "pairwise.complete.obs")
cor.test(JEID_2$jeid03, JEID_2$unive, use = "pairwise.complete.obs")
cor.test(JEID_2$jeid04, JEID_2$unive, use = "pairwise.complete.obs")
cor.test(JEID_2$jeid05, JEID_2$unive, use = "pairwise.complete.obs")

cor.test(JEID_2$jeid01, JEID_2$achie, use = "pairwise.complete.obs")
cor.test(JEID_2$jeid02, JEID_2$achie, use = "pairwise.complete.obs")
cor.test(JEID_2$jeid03, JEID_2$achie, use = "pairwise.complete.obs")
cor.test(JEID_2$jeid04, JEID_2$achie, use = "pairwise.complete.obs")
cor.test(JEID_2$jeid05, JEID_2$achie, use = "pairwise.complete.obs")

cor.test(JEID_2$jeid01, JEID_2$secur, use = "pairwise.complete.obs")
cor.test(JEID_2$jeid02, JEID_2$secur, use = "pairwise.complete.obs")
cor.test(JEID_2$jeid03, JEID_2$secur, use = "pairwise.complete.obs")
cor.test(JEID_2$jeid04, JEID_2$secur, use = "pairwise.complete.obs")
cor.test(JEID_2$jeid05, JEID_2$secur, use = "pairwise.complete.obs")

cor.test(JEID_2$jeid01, JEID_2$stimu, use = "pairwise.complete.obs")
cor.test(JEID_2$jeid02, JEID_2$stimu, use = "pairwise.complete.obs")
cor.test(JEID_2$jeid03, JEID_2$stimu, use = "pairwise.complete.obs")
cor.test(JEID_2$jeid04, JEID_2$stimu, use = "pairwise.complete.obs")
cor.test(JEID_2$jeid05, JEID_2$stimu, use = "pairwise.complete.obs")

cor.test(JEID_2$jeid01, JEID_2$confo, use = "pairwise.complete.obs")
cor.test(JEID_2$jeid02, JEID_2$confo, use = "pairwise.complete.obs")
cor.test(JEID_2$jeid03, JEID_2$confo, use = "pairwise.complete.obs")
cor.test(JEID_2$jeid04, JEID_2$confo, use = "pairwise.complete.obs")
cor.test(JEID_2$jeid05, JEID_2$confo, use = "pairwise.complete.obs")

cor.test(JEID_2$jeid01, JEID_2$tradi, use = "pairwise.complete.obs")
cor.test(JEID_2$jeid02, JEID_2$tradi, use = "pairwise.complete.obs")
cor.test(JEID_2$jeid03, JEID_2$tradi, use = "pairwise.complete.obs")
cor.test(JEID_2$jeid04, JEID_2$tradi, use = "pairwise.complete.obs")
cor.test(JEID_2$jeid05, JEID_2$tradi, use = "pairwise.complete.obs")
```

```
cor.test(JEID_2$jeid01, JEID_2$hedon, use = "pairwise.complete.obs")
cor.test(JEID_2$jeid02, JEID_2$hedon, use = "pairwise.complete.obs")
cor.test(JEID_2$jeid03, JEID_2$hedon, use = "pairwise.complete.obs")
cor.test(JEID_2$jeid04, JEID_2$hedon, use = "pairwise.complete.obs")
cor.test(JEID_2$jeid05, JEID_2$hedon, use = "pairwise.complete.obs")

cor.test(JEID_2$jeid01, JEID_2$benev, use = "pairwise.complete.obs")
cor.test(JEID_2$jeid02, JEID_2$benev, use = "pairwise.complete.obs")
cor.test(JEID_2$jeid03, JEID_2$benev, use = "pairwise.complete.obs")
cor.test(JEID_2$jeid04, JEID_2$benev, use = "pairwise.complete.obs")
cor.test(JEID_2$jeid05, JEID_2$benev, use = "pairwise.complete.obs")

##Sociodemographic characteristics

#Gender (1 = male, 2 = female)
describe(JEID_2$sex)
cor.test(JEID_2$jeid01, JEID_2$sex, use = "pairwise.complete.obs")
cor.test(JEID_2$jeid02, JEID_2$sex, use = "pairwise.complete.obs")
cor.test(JEID_2$jeid03, JEID_2$sex, use = "pairwise.complete.obs")
cor.test(JEID_2$jeid04, JEID_2$sex, use = "pairwise.complete.obs")
cor.test(JEID_2$jeid05, JEID_2$sex, use = "pairwise.complete.obs")

#Age
describe(JEID_2$age)
cor.test(JEID_2$jeid01, JEID_2$age, use = "pairwise.complete.obs")
cor.test(JEID_2$jeid02, JEID_2$age, use = "pairwise.complete.obs")
cor.test(JEID_2$jeid03, JEID_2$age, use = "pairwise.complete.obs")
cor.test(JEID_2$jeid04, JEID_2$age, use = "pairwise.complete.obs")
cor.test(JEID_2$jeid05, JEID_2$age, use = "pairwise.complete.obs")

#Educational attainment
describe(JEID_2$edu)
cor.test(JEID_2$jeid01, JEID_2$edu, use = "pairwise.complete.obs")
cor.test(JEID_2$jeid02, JEID_2$edu, use = "pairwise.complete.obs")
cor.test(JEID_2$jeid03, JEID_2$edu, use = "pairwise.complete.obs")
cor.test(JEID_2$jeid04, JEID_2$edu, use = "pairwise.complete.obs")
cor.test(JEID_2$jeid05, JEID_2$edu, use = "pairwise.complete.obs")

#Gross income
describe(JEID_2$inco02tb)
JEID_2$inco02 <- recode(JEID_2$inco02tb, "-99 = NA; else = JEID_2$inco02tb")
describe(JEID_2$inco02)

cor.test(JEID_2$jeid01, JEID_2$inco02, use = "pairwise.complete.obs")
cor.test(JEID_2$jeid02, JEID_2$inco02, use = "pairwise.complete.obs")
cor.test(JEID_2$jeid03, JEID_2$inco02, use = "pairwise.complete.obs")
```

```
cor.test(JEID_2$jeid04, JEID_2$inco02, use = "pairwise.complete.obs")
cor.test(JEID_2$jeid05, JEID_2$inco02, use = "pairwise.complete.obs")

##Justice consequences

#General life satisfaction
describe(JEID_2$gls)
cor.test(JEID_2$jeid01, JEID_2$gls, use = "pairwise.complete.obs")
cor.test(JEID_2$jeid02, JEID_2$gls, use = "pairwise.complete.obs")
cor.test(JEID_2$jeid03, JEID_2$gls, use = "pairwise.complete.obs")
cor.test(JEID_2$jeid04, JEID_2$gls, use = "pairwise.complete.obs")
cor.test(JEID_2$jeid05, JEID_2$gls, use = "pairwise.complete.obs")

#General health
describe(JEID_2$healthr)
cor.test(JEID_2$jeid01, JEID_2$healthr, use = "pairwise.complete.obs")
cor.test(JEID_2$jeid02, JEID_2$healthr, use = "pairwise.complete.obs")
cor.test(JEID_2$jeid03, JEID_2$healthr, use = "pairwise.complete.obs")
cor.test(JEID_2$jeid04, JEID_2$healthr, use = "pairwise.complete.obs")
cor.test(JEID_2$jeid05, JEID_2$healthr, use = "pairwise.complete.obs")

#Trust in government
describe(JEID_2$poltr)
JEID_2$poltrust <- recode(JEID_2$poltr, "1 = 1; 2 = 2; 3 = 3; 4 = 4; 5 = 5;
                          6 = 6; 7 = 7; 8 = 8; 9 = 9; 10 = 10;
                          11 = 11; else = NA")
describe(JEID_2$poltrust)

cor.test(JEID_2$jeid01, JEID_2$poltrust, use = "pairwise.complete.obs")
cor.test(JEID_2$jeid02, JEID_2$poltrust, use = "pairwise.complete.obs")
cor.test(JEID_2$jeid03, JEID_2$poltrust, use = "pairwise.complete.obs")
cor.test(JEID_2$jeid04, JEID_2$poltrust, use = "pairwise.complete.obs")
cor.test(JEID_2$jeid05, JEID_2$poltrust, use = "pairwise.complete.obs")

##Social desirability

describe(JEID_2$ksepq01)
JEID_2$SDPQ <- JEID_2$ksepq01+JEID_2$ksepq02+JEID_2$ksepq03
JEID_2$SDNQ <- JEID_2$ksenq04r+JEID_2$ksenq05r+JEID_2$ksenq06r
describe(JEID_2$SDPQ)
describe(JEID_2$SDNQ)

cor.test(JEID_2$jeid01, JEID_2$SDPQ, use = "pairwise.complete.obs")
cor.test(JEID_2$jeid02, JEID_2$SDPQ, use = "pairwise.complete.obs")
cor.test(JEID_2$jeid03, JEID_2$SDPQ, use = "pairwise.complete.obs")
cor.test(JEID_2$jeid04, JEID_2$SDPQ, use = "pairwise.complete.obs")
```

```
cor.test(JEID_2$jeid05, JEID_2$SDPQ, use = "pairwise.complete.obs")

cor.test(JEID_2$jeid01, JEID_2$SDNQ, use = "pairwise.complete.obs")
cor.test(JEID_2$jeid02, JEID_2$SDNQ, use = "pairwise.complete.obs")
cor.test(JEID_2$jeid03, JEID_2$SDNQ, use = "pairwise.complete.obs")
cor.test(JEID_2$jeid04, JEID_2$SDNQ, use = "pairwise.complete.obs")
cor.test(JEID_2$jeid05, JEID_2$SDNQ, use = "pairwise.complete.obs")

##########################################################################

################################
#Step 7: Descriptive statistics
################################

#Age
summary(JEID_2$age)
sd(JEID_2$age)

#Proportion of women
table(JEID_2$sex)

#Educational level
table(JEID_2$quota)
#Quote 1: male, lower education, 18-29
#Quote 2: male, lower education, 30-49
#Quote 3: male, lower education, 50-69
#Quote 4: male, middle education, 18-29
#Quote 5: male, middle education, 30-49
#Quote 6: male, middle education, 50-69
#Quote 7: male, upper education, 18-29
#Quote 8: male, upper education, 30-49
#Quote 9: male, upper education, 50-69
#Quote 10: female, lower education, 18-29
#Quote 11: female, lower education, 30-49
#Quote 12: female, lower education, 50-69
#Quote 13: female, middle education, 18-29
#Quote 14: female, middle education, 30-49
#Quote 15: female, middle education, 50-69
#Quote 16: female, upper education, 18-29
#Quote 17: female, upper education, 30-49
#Quote 18: female, upper education, 50-69
edu1 <- subset(JEID_2, quota == 1 | quota == 2 | quota == 3 | quota == 10 |
        quota == 11 | quota == 12)
edu2 <- subset(JEID_2, quota == 4 | quota == 5 | quota == 6 | quota == 13 |
        quota == 14 | quota == 15)
edu3 <- subset(JEID_2, quota == 7 | quota == 8 | quota == 9 | quota == 16 |
```

```
        quota == 17 | quota == 18)

describe(edu1$quota)
describe(edu2$quota)
describe(edu3$quota)

##############################################################################
```

```
####################
#Analysis of Study 3
####################

#Clear workspace (run if desired)
rm(list = ls())

# #List of project directories
# dirs <- list(
#   data = "...",
#   analysis = "...")

#Load required packages
if (!require(car)) {install.packages("car")}; library(car)
if (!require(psych)) {install.packages("psych")}; library(psych)
if (!require(data.table)) {install.packages("data.table")}; library(data.table)
if (!require(dplyr)) {install.packages("dplyr")}; library(dplyr)
if (!require(MplusAutomation)){install.packages("MplusAutomation")}; library(MplusAutomation)
library(foreign)
if (!require(factoextra)) {install.packages("factoextra")}; library(factoextra)
if (!require(cluster)) {install.packages("cluster")}; library(cluster)
if (!require(ggplot2)) {install.packages("ggplot2")}; library(ggplot2)
if (!require(ggridges)) {install.packages("ggridges")}; library(ggridges)

#Load dataset
load(paste0(dirs$data, "JEID_3.rda"))

######################################################################

############################
#Step 0: Preparation of data
############################

#Recode negative-worded items (and response scales)
JEID_3 <- mutate(JEID_3,
        bsjne01r = 6-bsjne01,
        bsjeq02r = 6-bsjeq02,
        bsjea03r = 6-bsjea03,
        bsjen04r = 6-bsjen04,
        bsjne05r = 6-bsjne05,
        bsjen06r = 6-bsjen06,
        bsjea07r = 6-bsjea07,
        bsjeq08r = 6-bsjeq08,
        bsjeq09r = 6-bsjeq09,
        bsjne10r = 6-bsjne10,
        bsjea11r = 6-bsjea11,
```

```
        bsjen12r = 6-bsjen12,
        healthr = 6-health,
        ksenq04r = 6-ksenq04,
        ksenq05r = 6-ksenq05,
        ksenq06r = 6-ksenq06)

#Build mean scale values
JEID_3 <- mutate(JEID_3, jeid = (jedgv01+jeddv02+jedgu03+jedbe04+jedtp05)/5)
JEID_3 <- mutate(JEID_3, jeidr = (jedgv01r+jeddv02r+jedgu03r+jedbe04r+jedtp05r)/5)

#Split samples
JEID_DE <- subset(JEID_3, country == 1)
JEID_UK <- subset(JEID_3, country == 2)

##########################################################################

##################################
#Step 1: Scale values and graphs
##################################

##Germany

describe(JEID_DE$jedgv01)
describe(JEID_DE$jeddv02)
describe(JEID_DE$jedgu03)
describe(JEID_DE$jedbe04)
describe(JEID_DE$jedtp05)
describe(JEID_DE$jeid)

barplot(table(JEID_DE$jedgv01), xlab = "Answer categories", ylab = "Frequencies")
barplot(table(JEID_DE$jeddv02), xlab = "Answer categories", ylab = "Frequencies")
barplot(table(JEID_DE$jedgu03), xlab = "Answer categories", ylab = "Frequencies")
barplot(table(JEID_DE$jedbe04), xlab = "Answer categories", ylab = "Frequencies")
barplot(table(JEID_DE$jedtp05), xlab = "Answer categories", ylab = "Frequencies")
barplot(table(JEID_DE$jeid), xlab = "Answer categories", ylab = "Frequencies")

#Reduce dataset to JEID items
JEID_DE_only <- JEID_DE%>% dplyr::select(jedgv01:jedtp05)

#Rename JEID items
JEID_DE_only <- rename(JEID_DE_only, "Low (P10)" = jedgv01)
JEID_DE_only <- rename(JEID_DE_only, "Middle (P50)" = jeddv02)
JEID_DE_only <- rename(JEID_DE_only, "Upper-middle (P80)" = jedgu03)
JEID_DE_only <- rename(JEID_DE_only, "High (P90)" = jedbe04)
JEID_DE_only <- rename(JEID_DE_only, "Top (P99)" = jedtp05)
```

```
#Save dataset
save(JEID_DE_only, file = paste0(dirs$data, "JEID_DE_only.Rda"))

#Convert dataset to Excel
write.csv2(JEID_DE_only, "JEID_DE_plot.csv")

#Restructure dataset in Excel so that
#the second column contains the answers to item 1 of all persons,
#below that the answers to item 2, etc.
#The first column contains the corresponding item name.

#Load Excel dataset
JEID_DE_plot <- read.csv(paste0(dirs$data, "JEID_DE_plot.csv"), header = TRUE, sep = ";")

#Specify item order
JEID_DE_plot <- JEID_DE_plot %>%
  mutate(Item = fct_relevel(Item, levels = "Top (P99)", "High (P90)",
                            "Upper-middle (P80)", "Middle (P50)","Low (P10)"))

#Create graph
ggplot(JEID_DE_plot, aes(x = Answer, y = fct_rev(Item), height = stat(density))) +
  xlab("Answer on the rating scale") + ylab("Income group") +
  geom_density_ridges(stat = "binline", bins = 7, scale = 0.85, draw_baseline = FALSE) +
  scale_x_continuous(breaks = seq(1, 7, 1))

##########################################################################

##UK

describe(JEID_UK$jedgv01)
describe(JEID_UK$jeddv02)
describe(JEID_UK$jedgu03)
describe(JEID_UK$jedbe04)
describe(JEID_UK$jedtp05)
describe(JEID_UK$jeid)

barplot(table(JEID_UK$jedgv01), xlab = "Answer categories", ylab = "Frequencies")
barplot(table(JEID_UK$jeddv02), xlab = "Answer categories", ylab = "Frequencies")
barplot(table(JEID_UK$jedgu03), xlab = "Answer categories", ylab = "Frequencies")
barplot(table(JEID_UK$jedbe04), xlab = "Answer categories", ylab = "Frequencies")
barplot(table(JEID_UK$jedtp05), xlab = "Answer categories", ylab = "Frequencies")
barplot(table(JEID_UK$jeid), xlab = "Answer categories", ylab = "Frequencies")

#Reduce dataset to JEID items
JEID_UK_only <- JEID_UK%>% dplyr::select(jedgv01:jedtp05)
```

```
#Rename JEID items
JEID_UK_only <- rename(JEID_UK_only, "Low (P10)" = jedgv01)
JEID_UK_only <- rename(JEID_UK_only, "Middle (P50)" = jeddv02)
JEID_UK_only <- rename(JEID_UK_only, "Upper-middle (P80)" = jedgu03)
JEID_UK_only <- rename(JEID_UK_only, "High (P90)" = jedbe04)
JEID_UK_only <- rename(JEID_UK_only, "Top (P99)" = jedtp05)

#Save dataset
save(JEID_UK_only, file = paste0(dirs$data, "JEID_UK_only.Rda"))

#Convert dataset to Excel
write.csv2(JEID_UK_only, "JEID_UK_plot.csv")

#Restructure dataset in Excel so that
#the second column contains the answers to item 1 of all persons,
#below that the answers to item 2, etc.
#The first column contains the corresponding item name.

#Load Excel dataset
JEID_UK_plot <- read.csv(paste0(dirs$data, "JEID_UK_plot.csv"), header = TRUE, sep = ";")

#Specify item order
JEID_UK_plot <- JEID_UK_plot %>%
  mutate(Item = fct_relevel(Item, levels = "Top (P99)", "High (P90)",
                    "Upper-middle (P80)", "Middle (P50)","Low (P10)"))
#Create graph
ggplot(JEID_UK_plot, aes(x = Answer, y = fct_rev(Item), height = stat(density))) +
  xlab("Answer on the rating scale") + ylab("Income group") +
  geom_density_ridges(stat = "binline", bins = 7, scale = 0.85, draw_baseline = FALSE) +
  scale_x_continuous(breaks = seq(1, 7, 1))

################################################################################

#####################
#Step 2: Reliability
#####################

###Retest reliability

##Germany
cor.test(JEID_DE$jedgv01, JEID_DE$jedgv01r, use = "pairwise.complete.obs")
cor.test(JEID_DE$jeddv02, JEID_DE$jeddv02r, use = "pairwise.complete.obs")
cor.test(JEID_DE$jedgu03, JEID_DE$jedgu03r, use = "pairwise.complete.obs")
cor.test(JEID_DE$jedbe04, JEID_DE$jedbe04r, use = "pairwise.complete.obs")
cor.test(JEID_DE$jedtp05, JEID_DE$jedtp05r, use = "pairwise.complete.obs")
```

```
##########################################################################

##UK
cor.test(JEID_UK$jedgv01, JEID_UK$jedgv01r, use = "pairwise.complete.obs")
cor.test(JEID_UK$jeddv02, JEID_UK$jeddv02r, use = "pairwise.complete.obs")
cor.test(JEID_UK$jedgu03, JEID_UK$jedgu03r, use = "pairwise.complete.obs")
cor.test(JEID_UK$jedbe04, JEID_UK$jedbe04r, use = "pairwise.complete.obs")
cor.test(JEID_UK$jedtp05, JEID_UK$jedtp05r, use = "pairwise.complete.obs")

##########################################################################

###Individual profile correlation

##Germany

#Reduce data to JEID items
JEID_DE_only <- JEID_DE %>% dplyr::select(lfdn, jedgv01:jedtp05, jedgv01r:jedtp05r)
save(JEID_DE_only, file = paste0(dirs$data, "JEID_DE_only.Rda"))

#Compute the correlation between two sets of items (1:5 and 6:10)
#for each person and append that "ipsative correlation" to the data
JEID_DE_only_r <- JEID_DE_only %>%
  group_by(lfdn) %>%
  mutate(ipscor = cor(x = c_across(jedgv01:jedtp05),
                    y = c_across(jedgv01r:jedtp05r))) %>%
  ungroup()
JEID_DE_only_r

#Delete cases with missing values
JEID_DE_only_r <- na.omit(JEID_DE_only_r)
save(JEID_DE_only_r, file = paste0(dirs$data, "JEID_DE_only_r.Rda"))

#Summarize this correlation in the sample
JEID_DE_only_r %>% summarize(mean_ipscor = mean(ipscor))

##########################################################################

##UK

#Reduce data to JEID items
JEID_UK_only <- JEID_UK %>% dplyr::select(lfdn, jedgv01:jedtp05, jedgv01r:jedtp05r)
save(JEID_UK_only, file = paste0(dirs$data, "JEID_UK_only.Rda"))

#Compute the correlation between two sets of items (1:5 and 6:10)
#for each person and append that "ipsative correlation" to the data
JEID_UK_only_r <- JEID_UK_only %>%
```

```
  group_by(lfdn) %>%
  mutate(ipscor = cor(x = c_across(jedgv01:jedtp05),
                      y = c_across(jedgv01r:jedtp05r))) %>%
  ungroup()
JEID_UK_only_r

#Delete cases with missing values
JEID_UK_only_r <- na.omit(JEID_UK_only_r)
save(JEID_UK_only_r, file = paste0(dirs$data, "JEID_UK_only_r.Rda"))

#Summarize this correlation in the sample
JEID_UK_only_r %>% summarize(mean_ipscor = mean(ipscor))

###############################################################################

##############################################
#Step 3: Multigroup Latent Profile Analysis
##############################################

###Prepare dataset for MG-LPAs in Mplus
prepareMplusData(df = JEID_3, filename = "JEID_3.dat", inpfile = TRUE,
                 keepCols = c("country", "jedgv01", "jeddv02", "jedgu03", "jedbe04", "jedtp05"))

##Sample Mplus code (3 profile solution)

#Step 1

# TITLE:          JEID - Latent profile analysis (3 categories)
#
# DATA:           FILE = "JEID_3.dat";
#
# VARIABLE:
# NAMES = country jedgv01 jeddv02 jedgu03 jedbe04 jedtp05;
# CLASSES = c(3);
# MISSING = .;
#
# ANALYSIS:
# TYPE = MIXTURE;
# STARTS = 20000 5000;
# STITERATIONS = 50;
#
# MODEL:
# %overall%
# c on country@0;

#Step 2
```

```
# TITLE:          JEID - Latent profile analysis (3 categories)
#
# DATA:           FILE = "JEID_3.dat";
#
# VARIABLE:
# NAMES = country jedgv01 jeddv02 jedgu03 jedbe04 jedtp05;
# CLASSES = c(3);
# MISSING = .;
#
# ANALYSIS:
# TYPE = MIXTURE;
# STARTS = 0;
# OPTSEED = 627952;        !the best loglikelihood value found in Step 1
# LRTSTARTS =  0 0 1000 250;
#
# MODEL:
# %overall%
# c on country@0;
#
# PLOT:
# TYPE = plot3;
# SERIES = jedgv01 (1) jeddv02 (2) jedgu03 (3) jedbe04 (4) jedtp05 (5);
#
# OUTPUT:         TECH11 TECH14;

###################################
#Step 4: k-means cluster analysis
###################################

###Germany

##Prepare data

#Reduce dataset to JEID-variables
JEID_DE_reduced <- JEID_DE %>% dplyr::select(jedgv01:jedtp05)

#Identifiy missing values
df <- na.omit(JEID_DE_reduced)

#Standardize the variables
df <- scale(df)
head(df, n = 20)

##Determine the optimal number of clusters
```

```
#Elbow method
fviz_nbclust(df, kmeans, method = "wss")

#Average silhouette method
fviz_nbclust(df, kmeans, method = "silhouette")

#Gap statistic method
gap_stat <- clusGap(df, FUN = kmeans, nstart = 25,
                    K.max = 10, B = 50)
print(gap_stat, method = "firstmax")
fviz_gap_stat(gap_stat)

##Extracting results

set.seed(123)

#k = 3
final <- kmeans(df, 3, nstart = 25)
print(final)

#Visualize the clusters
fviz_cluster(final, data = df)

#Add the point classifications to the original data
#and compute the mean of each variable by clusters
JEID_DE_reduced %>%
  mutate(Cluster = final$cluster) %>%
  group_by(Cluster) %>%
  summarise_all("mean")
# Cluster jedgv01 jeddv02 jedgu03 jedbe04 jedtp05
# <int>   <dbl>   <dbl>   <dbl>   <dbl>   <dbl>
# 1       1.47    2.44    3.78    4.17    5.66
# 2       1.54    2.72    5.46    5.79    6.83
# 3       4.16    4.01    4.18    4.08    4.03

#Build new data frame
jeid_k3 <- data.frame(group = c("Inequality averse (31,9%)",
                                "Inequality averse (31,9%)",
                                "Inequality averse (31,9%)",
                                "Inequality averse (31,9%)",
                                "Inequality averse (31,9%)",
                                "Bottom-inequality averse (49.1%)",
                                "Bottom-inequality averse (49.1%)",
                                "Bottom-inequality averse (49.1%)",
                                "Bottom-inequality averse (49.1%)",
                                "Bottom-inequality averse (49.1%)",
```

```
                              "Status quo justification (19.0%)",
                              "Status quo justification (19.0%)",
                              "Status quo justification (19.0%)",
                              "Status quo justification (19.0%)",
                              "Status quo justification (19.0%)"),
                    income = c("P10", "P50", "P80", "P90", "P99",
                               "P10", "P50", "P80", "P90", "P99",
                               "P10", "P50", "P80", "P90", "P99"),
                    answer = c(1.54, 2.72, 5.46, 5.79, 6.83,
                               1.47, 2.44, 3.78, 4.17, 5.66,
                               4.16, 4.01, 4.1, 4.08, 4.03))
print(jeid_k3)

#Relevel group factor to change the order of the legend
jeid_k3$group <- factor(jeid_k3$group,
                      levels = c("Inequality averse (31,9%)",
                                 "Bottom-inequality averse (49.1%)",
                                 "Status quo justification (19.0%)"))

#Visualize the mean values per group
jeid_k3 %>%
  ggplot(aes(x = factor(income, level = c('P10', 'P50', 'P80', 'P90', 'P99')),
             y = answer,
             group = group, color = group)) +
  geom_line() + geom_point() + theme_classic() +
  xlab("Income percentile") + ylab("Answer on the rating scale") +
  geom_line(aes(color = group), size = 1) +
  scale_color_manual(values = c("blue", "red", "green")) +
  guides(color = guide_legend("Justice evaluation group"))

##########################################################################

##UK

##Prepare data

#Reduce dataset to JEID-variables
JEID_UK_reduced <- JEID_UK %>% dplyr::select(jedgv01:jedtp05)

#Identifiy missing values
df <- na.omit(JEID_UK_reduced)

#Standardize the variables
df <- scale(df)
head(df, n = 20)
```

```
##Determine the optimal number of clusters

#Elbow method
fviz_nbclust(df, kmeans, method = "wss")

#Average silhouette method
fviz_nbclust(df, kmeans, method = "silhouette")

#Gap statistic method
gap_stat <- clusGap(df, FUN = kmeans, nstart = 25,
                    K.max = 10, B = 50)
print(gap_stat, method = "firstmax")
fviz_gap_stat(gap_stat)

##Extracting results

set.seed(123)

#k = 3
final <- kmeans(df, 3, nstart = 25)
print(final)

#Visualize the clusters
fviz_cluster(final, data = df)

#Add the point classifications to the original data
#and compute the mean of each variable by clusters
JEID_UK_reduced %>%
  mutate(Cluster = final$cluster) %>%
  group_by(Cluster) %>%
  summarise_all("mean")
# Cluster jedgv01 jeddv02 jedgu03 jedbe04 jedtp05
# <int>   <dbl>   <dbl>   <dbl>   <dbl>   <dbl>
# 1       3.83    4.14    4.33    4.28    4.46
# 2       1.54    1.94    3.29    4.18    5.72
# 3       1.71    3.28    4.74    5.72    6.73

#Build new data frame
jeid_k3 <- data.frame(group = c("Inequality averse (35.5%)",
                                "Inequality averse (35.5%)",
                                "Inequality averse (35.5%)",
                                "Inequality averse (35.5%)",
                                "Inequality averse (35.5%)",
                                "Bottom-inequality averse (37.0%)",
                                "Bottom-inequality averse (37.0%)",
                                "Bottom-inequality averse (37.0%)",
```

```
                "Bottom-inequality averse (37.0%)",
                "Bottom-inequality averse (37.0%)",
                "Status quo justification (27.5%)",
                "Status quo justification (27.5%)",
                "Status quo justification (27.5%)",
                "Status quo justification (27.5%)",
                "Status quo justification (27.5%)"),
           income = c("P10", "P50", "P80", "P90", "P99",
                "P10", "P50", "P80", "P90", "P99",
                "P10", "P50", "P80", "P90", "P99"),
           answer = c(1.71, 3.28, 4.74, 5.72, 6.73,
                1.54, 1.94, 3.29, 4.18, 5.72,
                3.83, 4.14, 4.33, 4.28, 4.46))

print(jeid_k3)

#Relevel group factor to change the order of the legend
jeid_k3$group <- factor(jeid_k3$group,
            levels = c("Inequality averse (35.5%)",
                 "Bottom-inequality averse (37.0%)",
                 "Status quo justification (27.5%)"))

#Visualize the mean values per group
jeid_k3 %>%
  ggplot(aes(x = factor(income, level = c('P10', 'P50', 'P80', 'P90', 'P99')),
       y = answer,
       group = group, color = group)) +
  geom_line() + geom_point() + theme_classic() +
  xlab("Income percentile") + ylab("Answer on the rating scale") +
  geom_line(aes(color = group), size = 1) +
  scale_color_manual(values = c("blue", "red", "green")) +
  guides(color = guide_legend("Justice evaluation group"))

detach("package:dplyr", unload = TRUE)

##########################################################################

#############################
#Step 5: Construct validity
#############################

###Germany

##Value orientations

#Left–right self-placement
```

```
describe(JEID_DE$leri)
JEID_DE$pleri <- recode(JEID_DE$leri, "1 = 1; 2 = 2; 3 = 3; 4 = 4; 5 = 5; 6 = 6;
                        7 = 7; 8 = 8; 9 = 9; 10 = 10; 11 = NA")
describe(JEID_DE$pleri)

cor.test(JEID_DE$jedgv01, JEID_DE$pleri, use = "pairwise.complete.obs")
cor.test(JEID_DE$jeddv02, JEID_DE$pleri, use = "pairwise.complete.obs")
cor.test(JEID_DE$jedgu03, JEID_DE$pleri, use = "pairwise.complete.obs")
cor.test(JEID_DE$jedbe04, JEID_DE$pleri, use = "pairwise.complete.obs")
cor.test(JEID_DE$jedtp05, JEID_DE$pleri, use = "pairwise.complete.obs")

##Sociodemographic characteristics

#Gender (1 = male, 2 = female)
describe(JEID_DE$sex)
cor.test(JEID_DE$jedgv01, JEID_DE$sex, use = "pairwise.complete.obs")
cor.test(JEID_DE$jeddv02, JEID_DE$sex, use = "pairwise.complete.obs")
cor.test(JEID_DE$jedgu03, JEID_DE$sex, use = "pairwise.complete.obs")
cor.test(JEID_DE$jedbe04, JEID_DE$sex, use = "pairwise.complete.obs")
cor.test(JEID_DE$jedtp05, JEID_DE$sex, use = "pairwise.complete.obs")

#Age
describe(JEID_DE$age)
cor.test(JEID_DE$jedgv01, JEID_DE$age, use = "pairwise.complete.obs")
cor.test(JEID_DE$jeddv02, JEID_DE$age, use = "pairwise.complete.obs")
cor.test(JEID_DE$jedgu03, JEID_DE$age, use = "pairwise.complete.obs")
cor.test(JEID_DE$jedbe04, JEID_DE$age, use = "pairwise.complete.obs")
cor.test(JEID_DE$jedtp05, JEID_DE$age, use = "pairwise.complete.obs")

#Educational attainment
describe(JEID_DE$edu)
cor.test(JEID_DE$jedgv01, JEID_DE$edu, use = "pairwise.complete.obs")
cor.test(JEID_DE$jeddv02, JEID_DE$edu, use = "pairwise.complete.obs")
cor.test(JEID_DE$jedgu03, JEID_DE$edu, use = "pairwise.complete.obs")
cor.test(JEID_DE$jedbe04, JEID_DE$edu, use = "pairwise.complete.obs")
cor.test(JEID_DE$jedtp05, JEID_DE$edu, use = "pairwise.complete.obs")

#Gross income
describe(JEID_DE$inco02tb)
JEID_DE$inco02 <- recode(JEID_DE$inco02tb, "-99 = NA; else = JEID_DE$inco02tb")
describe(JEID_DE$inco02)

cor.test(JEID_DE$jedgv01, JEID_DE$inco02, use = "pairwise.complete.obs")
cor.test(JEID_DE$jeddv02, JEID_DE$inco02, use = "pairwise.complete.obs")
cor.test(JEID_DE$jedgu03, JEID_DE$inco02, use = "pairwise.complete.obs")
cor.test(JEID_DE$jedbe04, JEID_DE$inco02, use = "pairwise.complete.obs")
```

```
cor.test(JEID_DE$jedtp05, JEID_DE$inco02, use = "pairwise.complete.obs")

##Justice consequences

#General life satisfaction
describe(JEID_DE$gls)
cor.test(JEID_DE$jedgv01, JEID_DE$gls, use = "pairwise.complete.obs")
cor.test(JEID_DE$jeddv02, JEID_DE$gls, use = "pairwise.complete.obs")
cor.test(JEID_DE$jedgu03, JEID_DE$gls, use = "pairwise.complete.obs")
cor.test(JEID_DE$jedbe04, JEID_DE$gls, use = "pairwise.complete.obs")
cor.test(JEID_DE$jedtp05, JEID_DE$gls, use = "pairwise.complete.obs")

#General health
describe(JEID_DE$healthr)
cor.test(JEID_DE$jedgv01, JEID_DE$healthr, use = "pairwise.complete.obs")
cor.test(JEID_DE$jeddv02, JEID_DE$healthr, use = "pairwise.complete.obs")
cor.test(JEID_DE$jedgu03, JEID_DE$healthr, use = "pairwise.complete.obs")
cor.test(JEID_DE$jedbe04, JEID_DE$healthr, use = "pairwise.complete.obs")
cor.test(JEID_DE$jedtp05, JEID_DE$healthr, use = "pairwise.complete.obs")

#########################################################################

###UK

##Value orientations

#Basic social justice orientation
if (JEID_UK$form [1]) JEID_UK$need1 <- JEID_UK$bsjne01r+JEID_UK$bsjne10r
if (JEID_UK$form [2]) JEID_UK$need2 <- JEID_UK$bsjne01r+JEID_UK$bsjne05r
if (JEID_UK$form [3]) JEID_UK$need3 <- JEID_UK$bsjne05r+JEID_UK$bsjne10r

if (JEID_UK$form [1]) JEID_UK$equi1 <- JEID_UK$bsjeq02r+JEID_UK$bsjeq09r
if (JEID_UK$form [2]) JEID_UK$equi2 <- JEID_UK$bsjeq02r+JEID_UK$bsjeq08r
if (JEID_UK$form [3]) JEID_UK$equi3 <- JEID_UK$bsjeq08r+JEID_UK$bsjeq09r

if (JEID_UK$form [1]) JEID_UK$equa1 <- JEID_UK$bsjea03r+JEID_UK$bsjea11r
if (JEID_UK$form [2]) JEID_UK$equa2 <- JEID_UK$bsjea03r+JEID_UK$bsjea07r
if (JEID_UK$form [3]) JEID_UK$equa3 <- JEID_UK$bsjea07r+JEID_UK$bsjea11r

if (JEID_UK$form [1]) JEID_UK$enti1 <- JEID_UK$bsjen04r+JEID_UK$bsjen12r
if (JEID_UK$form [2]) JEID_UK$enti2 <- JEID_UK$bsjen04r+JEID_UK$bsjen06r
if (JEID_UK$form [3]) JEID_UK$enti3 <- JEID_UK$bsjen06r+JEID_UK$bsjen12r

library(dplyr)

JEID_UK <- mutate(JEID_UK, need = coalesce(need1, need2, need3))
```

```
JEID_UK <- mutate(JEID_UK, equi = coalesce(equi1, equi2, equi3))
JEID_UK <- mutate(JEID_UK, equa = coalesce(equa1, equa2, equa3))
JEID_UK <- mutate(JEID_UK, enti = coalesce(enti1, enti2, enti3))

describe(JEID_UK$need)
describe(JEID_UK$equi)
describe(JEID_UK$equa)
describe(JEID_UK$enti)

cor.test(JEID_UK$jedgv01, JEID_UK$need, use = "pairwise.complete.obs")
cor.test(JEID_UK$jeddv02, JEID_UK$need, use = "pairwise.complete.obs")
cor.test(JEID_UK$jedgu03, JEID_UK$need, use = "pairwise.complete.obs")
cor.test(JEID_UK$jedbe04, JEID_UK$need, use = "pairwise.complete.obs")
cor.test(JEID_UK$jedtp05, JEID_UK$need, use = "pairwise.complete.obs")

cor.test(JEID_UK$jedgv01, JEID_UK$equi, use = "pairwise.complete.obs")
cor.test(JEID_UK$jeddv02, JEID_UK$equi, use = "pairwise.complete.obs")
cor.test(JEID_UK$jedgu03, JEID_UK$equi, use = "pairwise.complete.obs")
cor.test(JEID_UK$jedbe04, JEID_UK$equi, use = "pairwise.complete.obs")
cor.test(JEID_UK$jedtp05, JEID_UK$equi, use = "pairwise.complete.obs")

cor.test(JEID_UK$jedgv01, JEID_UK$equa, use = "pairwise.complete.obs")
cor.test(JEID_UK$jeddv02, JEID_UK$equa, use = "pairwise.complete.obs")
cor.test(JEID_UK$jedgu03, JEID_UK$equa, use = "pairwise.complete.obs")
cor.test(JEID_UK$jedbe04, JEID_UK$equa, use = "pairwise.complete.obs")
cor.test(JEID_UK$jedtp05, JEID_UK$equa, use = "pairwise.complete.obs")

cor.test(JEID_UK$jedgv01, JEID_UK$enti, use = "pairwise.complete.obs")
cor.test(JEID_UK$jeddv02, JEID_UK$enti, use = "pairwise.complete.obs")
cor.test(JEID_UK$jedgu03, JEID_UK$enti, use = "pairwise.complete.obs")
cor.test(JEID_UK$jedbe04, JEID_UK$enti, use = "pairwise.complete.obs")
cor.test(JEID_UK$jedtp05, JEID_UK$enti, use = "pairwise.complete.obs")

#Left–right self-placement
detach("package:dplyr", unload = TRUE)
describe(JEID_UK$leri)
JEID_UK$pleri <- recode(JEID_UK$leri, "1 = 1; 2 = 2; 3 = 3; 4 = 4; 5 = 5; 6 = 6;
                               7 = 7; 8 = 8; 9 = 9; 10 = 10; 11 = NA")
describe(JEID_UK$pleri)

cor.test(JEID_UK$jedgv01, JEID_UK$pleri, use = "pairwise.complete.obs")
cor.test(JEID_UK$jeddv02, JEID_UK$pleri, use = "pairwise.complete.obs")
cor.test(JEID_UK$jedgu03, JEID_UK$pleri, use = "pairwise.complete.obs")
cor.test(JEID_UK$jedbe04, JEID_UK$pleri, use = "pairwise.complete.obs")
cor.test(JEID_UK$jedtp05, JEID_UK$pleri, use = "pairwise.complete.obs")
```

```
#Human values
JEID_UK$hv01 <- recode(JEID_UK$hvesd01, "1 = 6;  2 = 5; 3 = 4; 4 = 3; 5 = 2; 6 = 1; else = NA")
JEID_UK$hv02 <- recode(JEID_UK$hvepo02, "1 = 6;  2 = 5; 3 = 4; 4 = 3; 5 = 2; 6 = 1; else = NA")
JEID_UK$hv03 <- recode(JEID_UK$hveun03, "1 = 6;  2 = 5; 3 = 4; 4 = 3; 5 = 2; 6 = 1; else = NA")
JEID_UK$hv04 <- recode(JEID_UK$hveac04, "1 = 6;  2 = 5; 3 = 4; 4 = 3; 5 = 2; 6 = 1; else = NA")
JEID_UK$hv05 <- recode(JEID_UK$hvesc05, "1 = 6;  2 = 5; 3 = 4; 4 = 3; 5 = 2; 6 = 1; else = NA")
JEID_UK$hv06 <- recode(JEID_UK$hvest06, "1 = 6;  2 = 5; 3 = 4; 4 = 3; 5 = 2; 6 = 1; else = NA")
JEID_UK$hv07 <- recode(JEID_UK$hveco07, "1 = 6;  2 = 5; 3 = 4; 4 = 3; 5 = 2; 6 = 1; else = NA")
JEID_UK$hv08 <- recode(JEID_UK$hveun08, "1 = 6;  2 = 5; 3 = 4; 4 = 3; 5 = 2; 6 = 1; else = NA")
JEID_UK$hv09 <- recode(JEID_UK$hvetr09, "1 = 6;  2 = 5; 3 = 4; 4 = 3; 5 = 2; 6 = 1; else = NA")
JEID_UK$hv10 <- recode(JEID_UK$hvehe10, "1 = 6;  2 = 5; 3 = 4; 4 = 3; 5 = 2; 6 = 1; else = NA")
JEID_UK$hv11 <- recode(JEID_UK$hvesd11, "1 = 6;  2 = 5; 3 = 4; 4 = 3; 5 = 2; 6 = 1; else = NA")
JEID_UK$hv12 <- recode(JEID_UK$hvebe12, "1 = 6;  2 = 5; 3 = 4; 4 = 3; 5 = 2; 6 = 1; else = NA")
JEID_UK$hv13 <- recode(JEID_UK$hveac13, "1 = 6;  2 = 5; 3 = 4; 4 = 3; 5 = 2; 6 = 1; else = NA")
JEID_UK$hv14 <- recode(JEID_UK$hvesc14, "1 = 6;  2 = 5; 3 = 4; 4 = 3; 5 = 2; 6 = 1; else = NA")
JEID_UK$hv15 <- recode(JEID_UK$hvest15, "1 = 6;  2 = 5; 3 = 4; 4 = 3; 5 = 2; 6 = 1; else = NA")
JEID_UK$hv16 <- recode(JEID_UK$hveco16, "1 = 6;  2 = 5; 3 = 4; 4 = 3; 5 = 2; 6 = 1; else = NA")
JEID_UK$hv17 <- recode(JEID_UK$hvepo17, "1 = 6;  2 = 5; 3 = 4; 4 = 3; 5 = 2; 6 = 1; else = NA")
JEID_UK$hv18 <- recode(JEID_UK$hvebe18, "1 = 6;  2 = 5; 3 = 4; 4 = 3; 5 = 2; 6 = 1; else = NA")
JEID_UK$hv19 <- recode(JEID_UK$hveun19, "1 = 6;  2 = 5; 3 = 4; 4 = 3; 5 = 2; 6 = 1; else = NA")
JEID_UK$hv20 <- recode(JEID_UK$hvetr20, "1 = 6;  2 = 5; 3 = 4; 4 = 3; 5 = 2; 6 = 1; else = NA")
JEID_UK$hv21 <- recode(JEID_UK$hvehe21, "1 = 6;  2 = 5; 3 = 4; 4 = 3; 5 = 2; 6 = 1; else = NA")

if (JEID_UK$form [1]) JEID_UK$selfd1 <- JEID_UK$hv01
if (JEID_UK$form [2]) JEID_UK$selfd2 <- (JEID_UK$hv01+JEID_UK$hv11)/2
if (JEID_UK$form [3]) JEID_UK$selfd3 <- JEID_UK$hv11

if (JEID_UK$form [1]) JEID_UK$power1 <- (JEID_UK$hv02+JEID_UK$hv17)/2
if (JEID_UK$form [2]) JEID_UK$power2 <- JEID_UK$hv02
if (JEID_UK$form [3]) JEID_UK$power3 <- JEID_UK$hv17

if (JEID_UK$form [1]) JEID_UK$unive1 <- (JEID_UK$hv03+JEID_UK$hv19)/2
if (JEID_UK$form [2]) JEID_UK$unive2 <- (JEID_UK$hv03+JEID_UK$hv08)/2
if (JEID_UK$form [3]) JEID_UK$unive3 <- (JEID_UK$hv08+JEID_UK$hv19)/2

if (JEID_UK$form [1]) JEID_UK$achie1 <- JEID_UK$hv04
if (JEID_UK$form [2]) JEID_UK$achie2 <- (JEID_UK$hv04+JEID_UK$hv13)/2
if (JEID_UK$form [3]) JEID_UK$achie3 <- JEID_UK$hv13

if (JEID_UK$form [1]) JEID_UK$secur1 <- JEID_UK$hv05
if (JEID_UK$form [2]) JEID_UK$secur2 <- (JEID_UK$hv05+JEID_UK$hv14)/2
if (JEID_UK$form [3]) JEID_UK$secur3 <- JEID_UK$hv14

if (JEID_UK$form [1]) JEID_UK$stimu1 <- (JEID_UK$hv06+JEID_UK$hv15)/2
if (JEID_UK$form [2]) JEID_UK$stimu2 <- JEID_UK$hv06
if (JEID_UK$form [3]) JEID_UK$stimu3 <- JEID_UK$hv15
```

```
if (JEID_UK$form [1]) JEID_UK$confo1 <- (JEID_UK$hv07+JEID_UK$hv16)/2
if (JEID_UK$form [2]) JEID_UK$confo2 <- JEID_UK$hv07
if (JEID_UK$form [3]) JEID_UK$confo3 <- JEID_UK$hv16

if (JEID_UK$form [1]) JEID_UK$tradi1 <- JEID_UK$hv20
if (JEID_UK$form [2]) JEID_UK$tradi2 <- JEID_UK$hv09
if (JEID_UK$form [3]) JEID_UK$tradi3 <- (JEID_UK$hv09+JEID_UK$hv20)/2

if (JEID_UK$form [1]) JEID_UK$hedon1 <- JEID_UK$hv21
if (JEID_UK$form [2]) JEID_UK$hedon2 <- JEID_UK$hv10
if (JEID_UK$form [3]) JEID_UK$hedon3 <- (JEID_UK$hv10+JEID_UK$hv21)/2

if (JEID_UK$form [1]) JEID_UK$benev1 <- JEID_UK$hv18
if (JEID_UK$form [2]) JEID_UK$benev2 <- JEID_UK$hv12
if (JEID_UK$form [3]) JEID_UK$benev3 <- (JEID_UK$hv12+JEID_UK$hv18)/2

library(dplyr)

JEID_UK <- mutate(JEID_UK, selfd = coalesce(selfd1, selfd2, selfd3))
JEID_UK <- mutate(JEID_UK, power = coalesce(power1, power2, power3))
JEID_UK <- mutate(JEID_UK, unive = coalesce(unive1, unive2, unive3))
JEID_UK <- mutate(JEID_UK, achie = coalesce(achie1, achie2, achie3))
JEID_UK <- mutate(JEID_UK, secur = coalesce(secur1, secur2, secur3))
JEID_UK <- mutate(JEID_UK, stimu = coalesce(stimu1, stimu2, stimu3))
JEID_UK <- mutate(JEID_UK, confo = coalesce(confo1, confo2, confo3))
JEID_UK <- mutate(JEID_UK, tradi = coalesce(tradi1, tradi2, tradi3))
JEID_UK <- mutate(JEID_UK, hedon = coalesce(hedon1, hedon2, hedon3))
JEID_UK <- mutate(JEID_UK, benev = coalesce(benev1, benev2, benev3))

describe(JEID_UK$selfd)
describe(JEID_UK$power)
describe(JEID_UK$unive)
describe(JEID_UK$achie)
describe(JEID_UK$secur)
describe(JEID_UK$stimu)
describe(JEID_UK$confo)
describe(JEID_UK$tradi)
describe(JEID_UK$hedon)
describe(JEID_UK$benev)

cor.test(JEID_UK$jedgv01, JEID_UK$selfd, use = "pairwise.complete.obs")
cor.test(JEID_UK$jeddv02, JEID_UK$selfd, use = "pairwise.complete.obs")
cor.test(JEID_UK$jedgu03, JEID_UK$selfd, use = "pairwise.complete.obs")
cor.test(JEID_UK$jedbe04, JEID_UK$selfd, use = "pairwise.complete.obs")
cor.test(JEID_UK$jedtp05, JEID_UK$selfd, use = "pairwise.complete.obs")
```

```
cor.test(JEID_UK$jedgv01, JEID_UK$power, use = "pairwise.complete.obs")
cor.test(JEID_UK$jeddv02, JEID_UK$power, use = "pairwise.complete.obs")
cor.test(JEID_UK$jedgu03, JEID_UK$power, use = "pairwise.complete.obs")
cor.test(JEID_UK$jedbe04, JEID_UK$power, use = "pairwise.complete.obs")
cor.test(JEID_UK$jedtp05, JEID_UK$power, use = "pairwise.complete.obs")

cor.test(JEID_UK$jedgv01, JEID_UK$unive, use = "pairwise.complete.obs")
cor.test(JEID_UK$jeddv02, JEID_UK$unive, use = "pairwise.complete.obs")
cor.test(JEID_UK$jedgu03, JEID_UK$unive, use = "pairwise.complete.obs")
cor.test(JEID_UK$jedbe04, JEID_UK$unive, use = "pairwise.complete.obs")
cor.test(JEID_UK$jedtp05, JEID_UK$unive, use = "pairwise.complete.obs")

cor.test(JEID_UK$jedgv01, JEID_UK$achie, use = "pairwise.complete.obs")
cor.test(JEID_UK$jeddv02, JEID_UK$achie, use = "pairwise.complete.obs")
cor.test(JEID_UK$jedgu03, JEID_UK$achie, use = "pairwise.complete.obs")
cor.test(JEID_UK$jedbe04, JEID_UK$achie, use = "pairwise.complete.obs")
cor.test(JEID_UK$jedtp05, JEID_UK$achie, use = "pairwise.complete.obs")

cor.test(JEID_UK$jedgv01, JEID_UK$secur, use = "pairwise.complete.obs")
cor.test(JEID_UK$jeddv02, JEID_UK$secur, use = "pairwise.complete.obs")
cor.test(JEID_UK$jedgu03, JEID_UK$secur, use = "pairwise.complete.obs")
cor.test(JEID_UK$jedbe04, JEID_UK$secur, use = "pairwise.complete.obs")
cor.test(JEID_UK$jedtp05, JEID_UK$secur, use = "pairwise.complete.obs")

cor.test(JEID_UK$jedgv01, JEID_UK$stimu, use = "pairwise.complete.obs")
cor.test(JEID_UK$jeddv02, JEID_UK$stimu, use = "pairwise.complete.obs")
cor.test(JEID_UK$jedgu03, JEID_UK$stimu, use = "pairwise.complete.obs")
cor.test(JEID_UK$jedbe04, JEID_UK$stimu, use = "pairwise.complete.obs")
cor.test(JEID_UK$jedtp05, JEID_UK$stimu, use = "pairwise.complete.obs")

cor.test(JEID_UK$jedgv01, JEID_UK$confo, use = "pairwise.complete.obs")
cor.test(JEID_UK$jeddv02, JEID_UK$confo, use = "pairwise.complete.obs")
cor.test(JEID_UK$jedgu03, JEID_UK$confo, use = "pairwise.complete.obs")
cor.test(JEID_UK$jedbe04, JEID_UK$confo, use = "pairwise.complete.obs")
cor.test(JEID_UK$jedtp05, JEID_UK$confo, use = "pairwise.complete.obs")

cor.test(JEID_UK$jedgv01, JEID_UK$tradi, use = "pairwise.complete.obs")
cor.test(JEID_UK$jeddv02, JEID_UK$tradi, use = "pairwise.complete.obs")
cor.test(JEID_UK$jedgu03, JEID_UK$tradi, use = "pairwise.complete.obs")
cor.test(JEID_UK$jedbe04, JEID_UK$tradi, use = "pairwise.complete.obs")
cor.test(JEID_UK$jedtp05, JEID_UK$tradi, use = "pairwise.complete.obs")

cor.test(JEID_UK$jedgv01, JEID_UK$hedon, use = "pairwise.complete.obs")
cor.test(JEID_UK$jeddv02, JEID_UK$hedon, use = "pairwise.complete.obs")
cor.test(JEID_UK$jedgu03, JEID_UK$hedon, use = "pairwise.complete.obs")
```

```
cor.test(JEID_UK$jedbe04, JEID_UK$hedon, use = "pairwise.complete.obs")
cor.test(JEID_UK$jedtp05, JEID_UK$hedon, use = "pairwise.complete.obs")

cor.test(JEID_UK$jedgv01, JEID_UK$benev, use = "pairwise.complete.obs")
cor.test(JEID_UK$jeddv02, JEID_UK$benev, use = "pairwise.complete.obs")
cor.test(JEID_UK$jedgu03, JEID_UK$benev, use = "pairwise.complete.obs")
cor.test(JEID_UK$jedbe04, JEID_UK$benev, use = "pairwise.complete.obs")
cor.test(JEID_UK$jedtp05, JEID_UK$benev, use = "pairwise.complete.obs")

##Sociodemographic characteristics

#Gender (1 = male, 2 = female)
describe(JEID_UK$sex)
cor.test(JEID_UK$jedgv01, JEID_UK$sex, use = "pairwise.complete.obs")
cor.test(JEID_UK$jeddv02, JEID_UK$sex, use = "pairwise.complete.obs")
cor.test(JEID_UK$jedgu03, JEID_UK$sex, use = "pairwise.complete.obs")
cor.test(JEID_UK$jedbe04, JEID_UK$sex, use = "pairwise.complete.obs")
cor.test(JEID_UK$jedtp05, JEID_UK$sex, use = "pairwise.complete.obs")

#Age
describe(JEID_UK$age)
cor.test(JEID_UK$jedgv01, JEID_UK$age, use = "pairwise.complete.obs")
cor.test(JEID_UK$jeddv02, JEID_UK$age, use = "pairwise.complete.obs")
cor.test(JEID_UK$jedgu03, JEID_UK$age, use = "pairwise.complete.obs")
cor.test(JEID_UK$jedbe04, JEID_UK$age, use = "pairwise.complete.obs")
cor.test(JEID_UK$jedtp05, JEID_UK$age, use = "pairwise.complete.obs")

#Educational attainment
describe(JEID_UK$edu)
cor.test(JEID_UK$jedgv01, JEID_UK$edu, use = "pairwise.complete.obs")
cor.test(JEID_UK$jeddv02, JEID_UK$edu, use = "pairwise.complete.obs")
cor.test(JEID_UK$jedgu03, JEID_UK$edu, use = "pairwise.complete.obs")
cor.test(JEID_UK$jedbe04, JEID_UK$edu, use = "pairwise.complete.obs")
cor.test(JEID_UK$jedtp05, JEID_UK$edu, use = "pairwise.complete.obs")

#Gross income
detach("package:dplyr", unload = TRUE)
describe(JEID_UK$inco02tb)
JEID_UK$inco02 <- recode(JEID_UK$inco02tb, "-99 = NA; else = JEID_UK$inco02tb")
describe(JEID_UK$inco02)

cor.test(JEID_UK$jedgv01, JEID_UK$inco02, use = "pairwise.complete.obs")
cor.test(JEID_UK$jeddv02, JEID_UK$inco02, use = "pairwise.complete.obs")
cor.test(JEID_UK$jedgu03, JEID_UK$inco02, use = "pairwise.complete.obs")
cor.test(JEID_UK$jedbe04, JEID_UK$inco02, use = "pairwise.complete.obs")
cor.test(JEID_UK$jedtp05, JEID_UK$inco02, use = "pairwise.complete.obs")
```

```
##Justice consequences

#General life satisfaction
describe(JEID_UK$gls)
cor.test(JEID_UK$jedgv01, JEID_UK$gls, use = "pairwise.complete.obs")
cor.test(JEID_UK$jeddv02, JEID_UK$gls, use = "pairwise.complete.obs")
cor.test(JEID_UK$jedgu03, JEID_UK$gls, use = "pairwise.complete.obs")
cor.test(JEID_UK$jedbe04, JEID_UK$gls, use = "pairwise.complete.obs")
cor.test(JEID_UK$jedtp05, JEID_UK$gls, use = "pairwise.complete.obs")

#General health
describe(JEID_UK$healthr)
cor.test(JEID_UK$jedgv01, JEID_UK$healthr, use = "pairwise.complete.obs")
cor.test(JEID_UK$jeddv02, JEID_UK$healthr, use = "pairwise.complete.obs")
cor.test(JEID_UK$jedgu03, JEID_UK$healthr, use = "pairwise.complete.obs")
cor.test(JEID_UK$jedbe04, JEID_UK$healthr, use = "pairwise.complete.obs")
cor.test(JEID_UK$jedtp05, JEID_UK$healthr, use = "pairwise.complete.obs")

##Social desirability

if (JEID_UK$form [1]) JEID_UK$SDPQ1 <- JEID_UK$ksepq01+JEID_UK$ksepq03
if (JEID_UK$form [2]) JEID_UK$SDPQ2 <- JEID_UK$ksepq01+JEID_UK$ksepq02
if (JEID_UK$form [3]) JEID_UK$SDPQ3 <- JEID_UK$ksepq02+JEID_UK$ksepq03

if (JEID_UK$form [1]) JEID_UK$SDNQ1 <- JEID_UK$ksenq04r+JEID_UK$ksenq06r
if (JEID_UK$form [2]) JEID_UK$SDNQ2 <- JEID_UK$ksenq04r+JEID_UK$ksenq05r
if (JEID_UK$form [3]) JEID_UK$SDNQ3 <- JEID_UK$ksenq05r+JEID_UK$ksenq06r

library(dplyr)

JEID_UK <- mutate(JEID_UK, SDPQ = coalesce(SDPQ1, SDPQ2, SDPQ3))
JEID_UK <- mutate(JEID_UK, SDNQ = coalesce(SDNQ1, SDNQ2, SDNQ3))

describe(JEID_UK$SDPQ)
describe(JEID_UK$SDNQ)

cor.test(JEID_UK$jedgv01, JEID_UK$SDPQ, use = "pairwise.complete.obs")
cor.test(JEID_UK$jeddv02, JEID_UK$SDPQ, use = "pairwise.complete.obs")
cor.test(JEID_UK$jedgu03, JEID_UK$SDPQ, use = "pairwise.complete.obs")
cor.test(JEID_UK$jedbe04, JEID_UK$SDPQ, use = "pairwise.complete.obs")
cor.test(JEID_UK$jedtp05, JEID_UK$SDPQ, use = "pairwise.complete.obs")

cor.test(JEID_UK$jedgv01, JEID_UK$SDNQ, use = "pairwise.complete.obs")
cor.test(JEID_UK$jeddv02, JEID_UK$SDNQ, use = "pairwise.complete.obs")
cor.test(JEID_UK$jedgu03, JEID_UK$SDNQ, use = "pairwise.complete.obs")
```

```
cor.test(JEID_UK$jedbe04, JEID_UK$SDNQ, use = "pairwise.complete.obs")
cor.test(JEID_UK$jedtp05, JEID_UK$SDNQ, use = "pairwise.complete.obs")

detach("package:dplyr", unload = TRUE)

###############################################################################

################################
#Step 6: Descriptive statistics
################################

##Germany

#Age
describe(JEID_DE$age)

#Proportion of women
table(JEID_DE$sex)

#Educational level
table(JEID_DE$quota)
#Quote 1: male, lower education, 18-29
#Quote 2: male, lower education, 30-49
#Quote 3: male, lower education, 50-65
#Quote 4: male, middle education, 18-29
#Quote 5: male, middle education, 30-49
#Quote 6: male, middle education, 50-65
#Quote 7: male, upper education, 18-29
#Quote 8: male, upper education, 30-49
#Quote 9: male, upper education, 50-65
#Quote 10: female, lower education, 18-29
#Quote 11: female, lower education, 30-49
#Quote 12: female, lower education, 50-65
#Quote 13: female, middle education, 18-29
#Quote 14: female, middle education, 30-49
#Quote 15: female, middle education, 50-65
#Quote 16: female, upper education, 18-29
#Quote 17: female, upper education, 30-49
#Quote 18: female, upper education, 50-65
edu1 <- subset(JEID_DE, quota == 1 | quota == 2 | quota == 3 | quota == 10 |
         quota == 11 | quota == 12)
edu2 <- subset(JEID_DE, quota == 4 | quota == 5 | quota == 6 | quota == 13 |
         quota == 14 | quota == 15)
edu3 <- subset(JEID_DE, quota == 7 | quota == 8 | quota == 9 | quota == 16 |
         quota == 17 | quota == 18)
```

```
describe(edu1$quota)
describe(edu2$quota)
describe(edu3$quota)

#Completion time
describe(JEID_DE$rtsJEID)
describe(JEID_DE$rtsJEIDr)

##########################################################

##UK

#Age
describe(JEID_UK$age)

#Proportion of women
table(JEID_UK$sex)

#Educational level
table(JEID_UK$quota)
#Quote 1: male, lower education, 18-29
#Quote 2: male, lower education, 30-49
#Quote 3: male, lower education, 50-65
#Quote 4: male, middle education, 18-29
#Quote 5: male, middle education, 30-49
#Quote 6: male, middle education, 50-659
#Quote 7: male, upper education, 18-29
#Quote 8: male, upper education, 30-49
#Quote 9: male, upper education, 50-65
#Quote 10: female, lower education, 18-29
#Quote 11: female, lower education, 30-49
#Quote 12: female, lower education, 50-65
#Quote 13: female, middle education, 18-29
#Quote 14: female, middle education, 30-49
#Quote 15: female, middle education, 50-65
#Quote 16: female, upper education, 18-29
#Quote 17: female, upper education, 30-49
#Quote 18: female, upper education, 50-65
edu1 <- subset(JEID_UK, quota == 1 | quota == 2 | quota == 3 | quota == 10 |
        quota == 11 | quota == 12)
edu2 <- subset(JEID_UK, quota == 4 | quota == 5 | quota == 6 | quota == 13 |
        quota == 14 | quota == 15)
edu3 <- subset(JEID_UK, quota == 7 | quota == 8 | quota == 9 | quota == 16 |
        quota == 17 | quota == 18)

describe(edu1$quota)
```

```
describe(edu2$quota)
describe(edu3$quota)

#Completion time
describe(JEID_UK$rtsJEID)
describe(JEID_UK$rtsJEIDr)

################################################################################

##########################
#Step 6: Reference values
##########################

##Germany

#Gender
tapply(JEID_DE$jedgv01, JEID_DE$sex, describe)
tapply(JEID_DE$jeddv02, JEID_DE$sex, describe)
tapply(JEID_DE$jedgu03, JEID_DE$sex, describe)
tapply(JEID_DE$jedbe04, JEID_DE$sex, describe)
tapply(JEID_DE$jedtp05, JEID_DE$sex, describe)

#Age
AGE1 <- subset(JEID_DE, age == 18 | age == 19 | age == 20 | age == 21 |
        age == 22 | age == 23 | age == 24 | age == 25 | age == 26 |
        age == 27 | age == 28 | age == 29)
AGE2 <- subset(JEID_DE, age == 30 | age == 31 | age == 32 | age == 33 |
        age == 34 | age == 35 | age == 36 | age == 37 |  age == 38 |
        age == 39 | age == 40 | age == 41 | age == 42 | age == 43 |
        age == 44 | age == 45 | age == 46 | age == 47 | age == 48 |
        age == 49)
AGE3 <- subset(JEID_DE, age == 50 | age == 51 | age == 52 | age == 53 |
        age == 54 | age == 55 | age == 56 | age == 57 | age == 58 |
        age == 59 | age == 60 | age == 61 | age == 62 | age == 63 |
        age == 64 | age == 65)

describe(AGE1$jedgv01)
describe(AGE1$jeddv02)
describe(AGE1$jedgu03)
describe(AGE1$jedbe04)
describe(AGE1$jedtp05)

describe(AGE2$jedgv01)
describe(AGE2$jeddv02)
describe(AGE2$jedgu03)
describe(AGE2$jedbe04)
```

```
describe(AGE2$jedtp05)

describe(AGE3$jedgv01)
describe(AGE3$jeddv02)
describe(AGE3$jedgu03)
describe(AGE3$jedbe04)
describe(AGE3$jedtp05)

#############################################################################

##UK

#Gender
tapply(JEID_UK$jedgv01, JEID_UK$sex, describe)
tapply(JEID_UK$jeddv02, JEID_UK$sex, describe)
tapply(JEID_UK$jedgu03, JEID_UK$sex, describe)
tapply(JEID_UK$jedbe04, JEID_UK$sex, describe)
tapply(JEID_UK$jedtp05, JEID_UK$sex, describe)

#Age
AGE1 <- subset(JEID_UK, age == 18 | age == 19 | age == 20 | age == 21 |
        age == 22 | age == 23 | age == 24 | age == 25 | age == 26 |
        age == 27 | age == 28 | age == 29)
AGE2 <- subset(JEID_UK, age == 30 | age == 31 | age == 32 | age == 33 |
        age == 34 | age == 35 | age == 36 | age == 37 |  age == 38 |
        age == 39 | age == 40 | age == 41 | age == 42 | age == 43 |
        age == 44 | age == 45 | age == 46 | age == 47 | age == 48 |
        age == 49)
AGE3 <- subset(JEID_UK, age == 50 | age == 51 | age == 52 | age == 53 |
        age == 54 | age == 55 | age == 56 | age == 57 | age == 58 |
        age == 59 | age == 60 | age == 61 | age == 62 | age == 63 |
        age == 64 | age == 65)

describe(AGE1$jedgv01)
describe(AGE1$jeddv02)
describe(AGE1$jedgu03)
describe(AGE1$jedbe04)
describe(AGE1$jedtp05)

describe(AGE2$jedgv01)
describe(AGE2$jeddv02)
describe(AGE2$jedgu03)
describe(AGE2$jedbe04)
describe(AGE2$jedtp05)

describe(AGE3$jedgv01)
```

```
describe(AGE3$jeddv02)
describe(AGE3$jedgu03)
describe(AGE3$jedbe04)
describe(AGE3$jedtp05)
```
