# Supplementary material for: Justice Evaluation of the Income Distribution (JEID): Development and validation of a short scale for the subjective assessment of objective differences in earnings
Source: PLoS One. 2023 Jan 26;18(1):e0281021. doi: 10.1371/journal.pone.0281021 (PMC9879472; doi:10.1371/journal.pone.0281021)
Supplement: S5 Appendix — (PDF) [file pone.0281021.s005.pdf]

S5 Appendix

Reference Ranges of the JEID Items Based on Study 3 for the Total Population and for Sex and Age Groups, Separately for Germany and the UK

	M		*SD*		Skewness		Kurtosis	
	DE	UK	DE	UK	DE	UK	DE	UK
Total population [N_{DE} = 420; N_{UK} = 440]								
Item 1	2.00	2.23	1.41	1.42	1.77	1.20	2.94	1.08
Item 2	2.83	3.02	1.26	1.30	0.30	0.24	0.07	−0.09
Item 3	4.39	4.09	1.15	1.19	0.13	0.05	1.02	1.35
Item 4	4.67	4.75	1.22	1.19	0.14	−0.01	0.19	0.44
Item 5	5.72	5.73	1.59	1.49	−1.19	−1.11	0.53	0.65
Male [n_{DE} = 205; n_{UK} = 221]								
Item 1	2.00	2.46	1.32	1.58	1.68	1.03	2.76	0.49
Item 2	2.88	3.27	1.26	1.31	0.20	0.20	−0.04	0.09
Item 3	4.28	4.21	1.16	1.13	0.13	0.29	1.18	1.44
Item 4	4.63	4.71	1.20	1.18	0.33	0.14	0.04	0.13
Item 5	5.58	5.62	1.65	1.48	−1.07	−0.82	0.21	−0.12
Female [n_{DE} = 215; n_{UK} = 219]								
Item 1	2.00	2.00	1.50	1.20	1.81	1.21	2.85	1.07
Item 2	2.78	2.77	1.27	1.24	0.39	0.26	0.16	−0.34
Item 3	4.49	3.97	1.13	1.24	0.16	−0.08	0.83	1.12
Item 4	4.71	4.80	1.23	1.21	−0.03	−0.15	0.33	0.73
Item 5	5.86	5.58	1.52	1.50	−1.30	−1.42	0.87	1.57
Age 18−29 [n_{DE} = 84; n_{UK} = 81]								
Item 1	2.44	2.16	1.75	1.32	1.31	0.97	0.77	0.51
Item 2	3.02	2.83	1.51	1.40	0.60	0.44	0.04	−0.34
Item 3	4.48	4.04	1.18	1.30	0.12	−0.03	0.29	0.56
Item 4	4.54	4.64	1.28	1.19	0.06	0.22	0.28	0.10
Item 5	5.52	5.63	1.69	1.43	−1.00	−0.74	−0.04	−0.19
Age 30−49 [n_{DE} = 173; n_{UK} = 186]								
Item 1	2.02	2.52	1.28	1.67	1.50	1.02	2.31	0.22
Item 2	2.81	3.21	1.21	1.37	0.09	0.29	−0.17	−0.02
Item 3	4.34	4.25	1.22	1.24	−0.29	0.09	1.17	0.99
Item 4	4.60	4.80	1.20	1.20	0.08	−0.07	0.24	0.32
Item 5	5.50	5.72	1.61	1.45	−0.91	−0.92	−0.05	−0.01
Age 50−65 [n_{DE} = 163; n_{UK} = 173]								
Item 1	1.76	1.94	1.30	1.09	2.25	0.92	5.13	0.08
Item 2	2.75	2.91	1.17	1.13	0.01	−0.16	−0.72	−0.76
Item 3	4.40	3.94	1.05	1.07	0.87	−0.09	0.77	2.30
Item 4	4.81	4.76	1.19	1.20	0.30	−0.06	−0.14	0.69
Item 5	6.06	5.79	1.48	1.57	−1.70	−1.41	2.18	1.42

Note. DE = Germany, UK = United Kingdom.
