# Supplementary material for: Justice Evaluation of the Income Distribution (JEID): Development and validation of a short scale for the subjective assessment of objective differences in earnings
Source: PLoS One. 2023 Jan 26;18(1):e0281021. doi: 10.1371/journal.pone.0281021 (PMC9879472; doi:10.1371/journal.pone.0281021)
Supplement: S6 Appendix — (PDF) [file pone.0281021.s006.pdf]

S6 Appendix

Table S6A

Results of the Latent Profile Analysis in Study 1

	1 class	2 classes	3 classes	4 classes	5 classes	6 classes
BIC	8,217.047	7,888.009	7,714.625	7,582.617	7,492.164	**7,441.327**
Entropy	-	.891	**.896**	.864	.850	.884
Class proportions						
Class 1	1.000	.712	.179	.535	.140	.457
Class 2	-	.288	.695	**.049**	.442	.146
Class 3	-	-	.126	.189	.160	.132
Class 4	-	-	-	.226	**.043**	.218
Class 5	-	-	-	-	.214	**.025**
Class 6	-	-	-	-	-	**.023**
Classification probabilities						
Class 1	-	.981	.870	.949	.914	.935
Class 2	-	.929	.985	.953	.915	.950
Class 3	-	-	.922	.963	.936	.918
Class 4	-	-	-	.846	.978	.873
Class 5	-	-	-	-	.864	.948
Class 6	-	-	-	-	-	.989
LMR-LRT	-	356.549	204.979	164.688	124.223	152.206
p value	-	<.001	.040	.007	.210	.017
B-LRT	-	366.155	210.501	169.125	127.570	156.307
p value	-	<.001	<.001	<.001	<.001	<.001

Note. BIC = Bayesian information criterion, LMR-LRT = Lo-Mendell-Rubin adjusted likelihood ratio test, B-LRT = Parametric bootstrap likelihood ratio test. In the case of BIC and entropy, the best (i.e., highest) values are in bold type. In the case of class proportions, the worst (i.e., < .05) values are in bold type. N = 486.

Table S6B

Results of the Latent Profile Analysis in Study 2

	1 class	2 classes	3 classes	4 classes	5 classes	6 classes	7 classes
BIC	12,451.779	12,121.419	11,926.256	11,739.571	11,568.362	11,489.591	**11,426.922**
Entropy	-	.879	.849	.832	.889	.878	**.918**
Class proportions							
Class 1	1.000	.773	.142	**.042**	**.045**	**.042**	.304
Class 2	-	.227	.647	.214	.170	.362	**.037**
Class 3	-	-	.210	.508	.230	.193	.178
Class 4	-	-	-	.236	.516	.207	.147
Class 5	-	-	-	-	**.039**	.150	.269
Class 6	-	-	-	-	-	**.045**	**.039**
Class 7	-	-	-	-	-	-	**.028**
Classification probabilities							
Class 1	-	.976	.816	.939	.918	.925	.885
Class 2	-	.933	.970	.862	.916	.907	.992
Class 3	-	-	.919	.924	.900	.985	.993
Class 4	-	-	-	.890	.947	.812	.914
Class 5	-	-	-	-	.918	.896	.964
Class 6	-	-	-	-	-	.984	.901
Class 7	-	-	-	-	-	-	1.000
LMR-LRT	-	359.593	227.813	219.550	204.465	172.843	98.669
p value	-	<.001	<.001	.001	.216	.153	.200
B-LRT	-	368.919	233.721	225.244	209.768	177.325	101.228
p value	-	<.001	<.001	<.001	<.001	<.001	<.001

Note. BIC = Bayesian information criterion, LMR-LRT = Lo-Mendell-Rubin adjusted likelihood ratio test, B-LRT = Parametric bootstrap likelihood ratio test. In the case of BIC and entropy, the best (i.e., highest) values are in bold type. In the case of class proportions, the worst (i.e., < .05) values are in bold type. N = 618.

Table S6C

Results of the Multigroup Latent Profile Analysis in Study 3

	1 class	2 classes	3 classes
BIC	**15,907.738**	14,129.853	13,827.562
Entropy	-	.864	**.890**
Class proportions			
Class 1	1.000	.253	.693
Class 2	-	.747	.120
Class 3	-	-	.187
Classification probabilities			
Class 1	-	.894	.969
Class 2	-	.981	.898
Class 3	-	-	.931
LMR-LRT	-	543.609	334.580
p value	-	<.001	<.001
B-LRT	-	557.018	342.832
p value	-	<.001	<.001

Note. BIC = Bayesian information criterion, LMR-LRT = Lo-Mendell-Rubin adjusted likelihood ratio test, B-LRT = Parametric bootstrap likelihood ratio test. N = 860 ($N_{Germany}$ = 420, N_{UK} = 440).
