# Supplementary material for: Justice Evaluation of the Income Distribution (JEID): Development and validation of a short scale for the subjective assessment of objective differences in earnings
Source: PLoS One. 2023 Jan 26;18(1):e0281021. doi: 10.1371/journal.pone.0281021 (PMC9879472; doi:10.1371/journal.pone.0281021)
Supplement: S7 Appendix — (PDF) [file pone.0281021.s007.pdf]

S7 Appendix

Sample Mean Distribution of the JEID Items Resulting From Latent Profile Analysis, by Profile Number and Study

Profile no.	Study 1	Study 2	Study 3
1	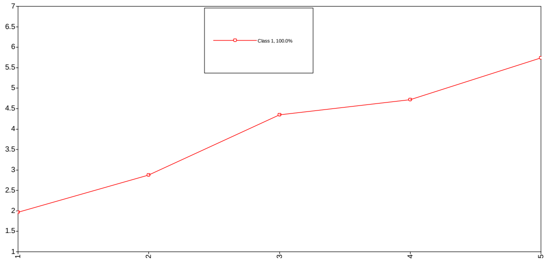	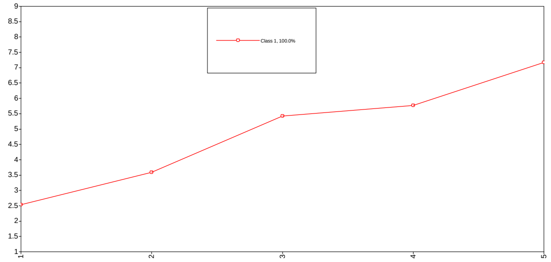	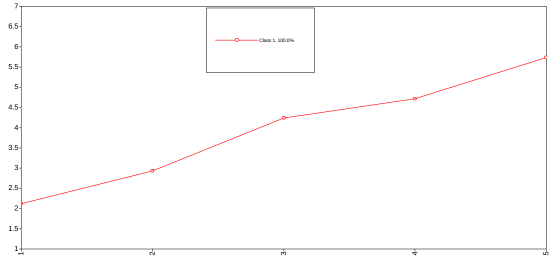
2	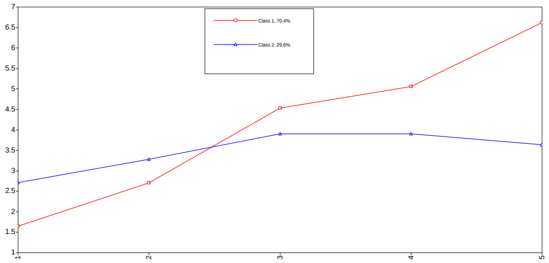	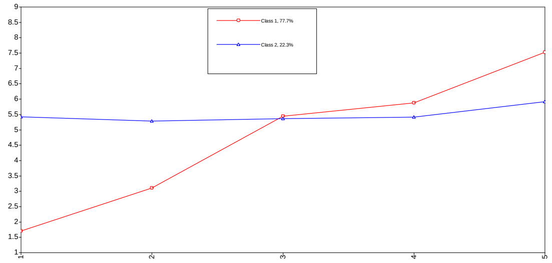	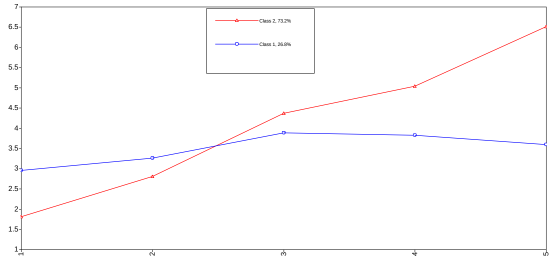
3	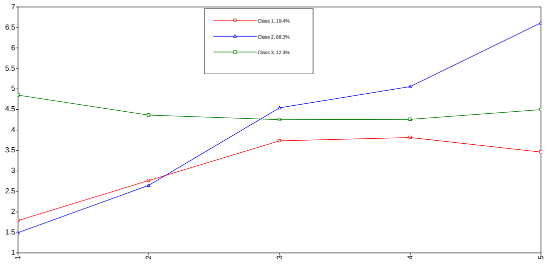	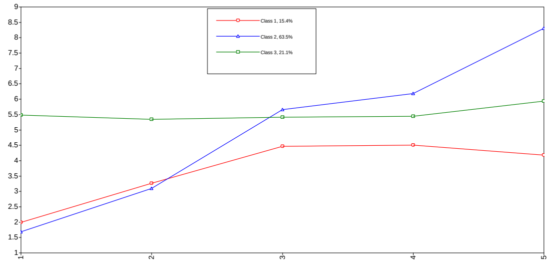	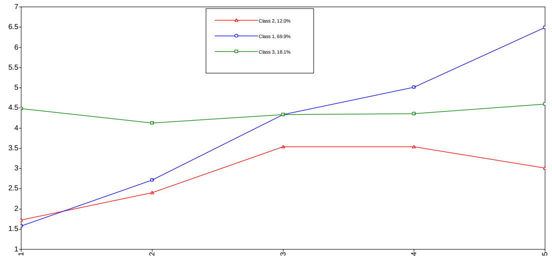

(continued)

<table>
<tr><th>Profile no.</th><th>Study 1</th><th>Study 2</th><th>Study 3</th></tr>
<tr><td>4</td><td>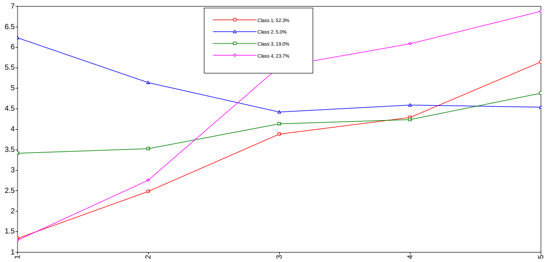
</td><td>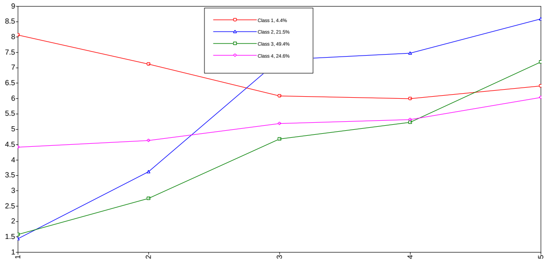
</td><td>-</td></tr>
<tr><td>5</td><td>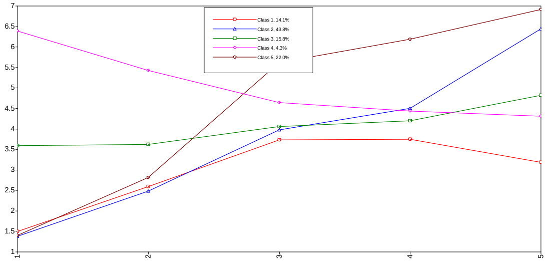
</td><td>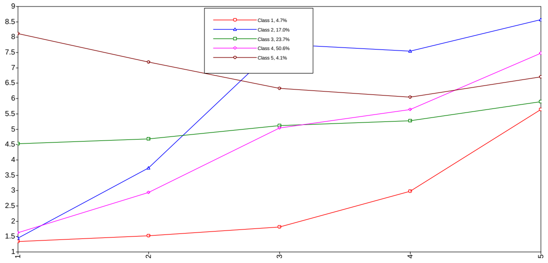
</td><td>-</td></tr>
<tr><td>6</td><td>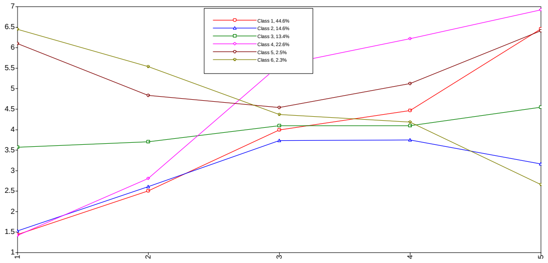
</td><td>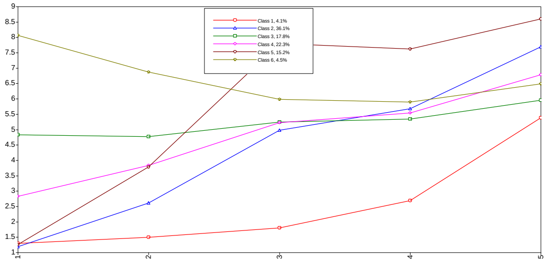
</td><td>-</td></tr>
<tr><td>7</td><td>-</td><td>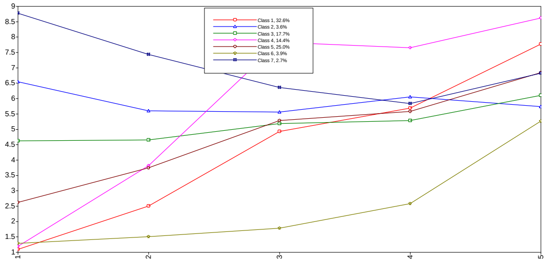
</td><td>-</td></tr>
</table>

Note. $N_{\text{Study 1}}$ = 489; $N_{\text{Study 2}}$ = 618; $N_{\text{Study 3}}$ = 860 (N_{Germany} 420, N_{UK} = 440).
