# Supplementary material for: Justice Evaluation of the Income Distribution (JEID): Development and validation of a short scale for the subjective assessment of objective differences in earnings
Source: PLoS One. 2023 Jan 26;18(1):e0281021. doi: 10.1371/journal.pone.0281021 (PMC9879472; doi:10.1371/journal.pone.0281021)
Supplement: S8 Appendix — (PDF) [file pone.0281021.s008.pdf]

S8 Appendix

Results of Three Methods to Extract the Optimal Number of Clusters, by Study

	Study 1	Study 2	Study 3	
			Germany	UK
Elbow method	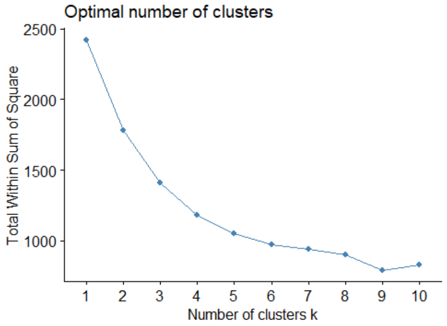	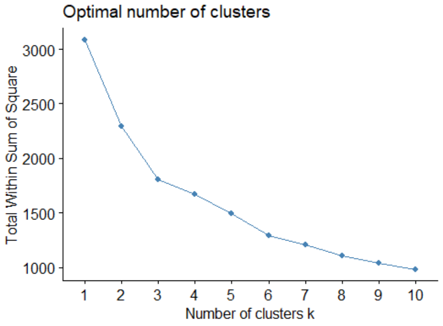	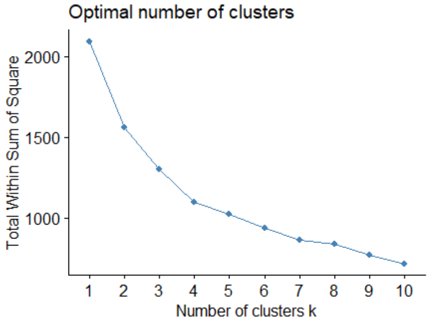	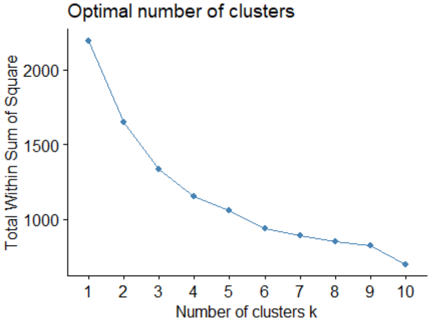
Average silhouette method	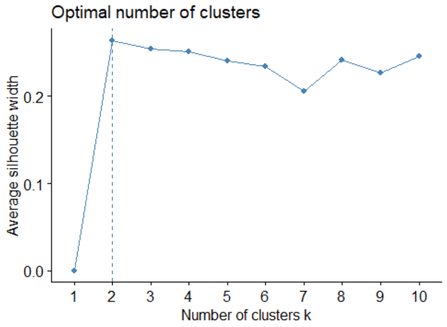	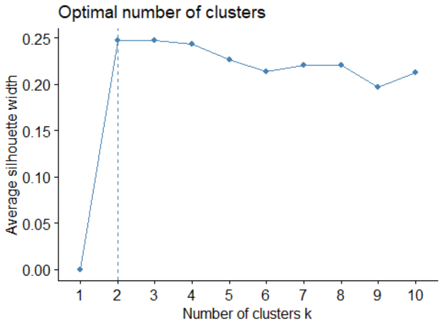	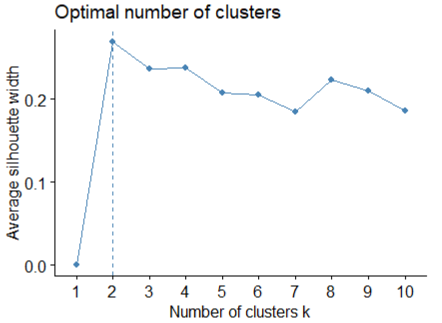	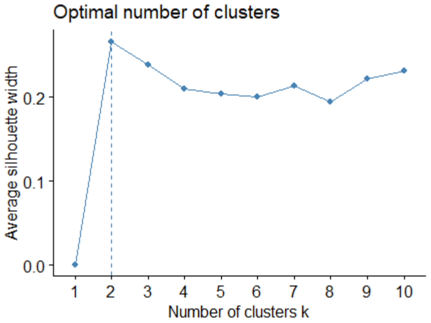

(continued)

	Study 1	Study 2	Study 3	
			Germany	UK
Gap statistic method	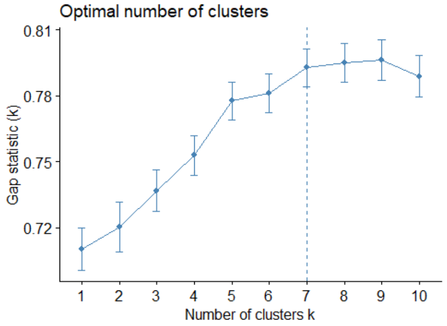	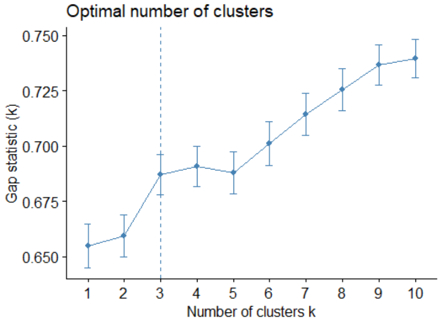	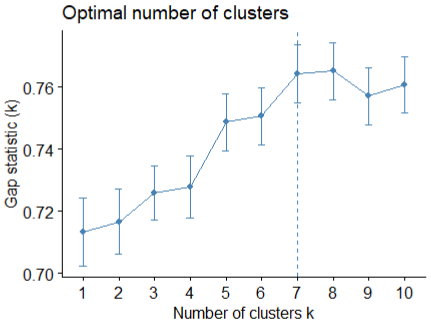	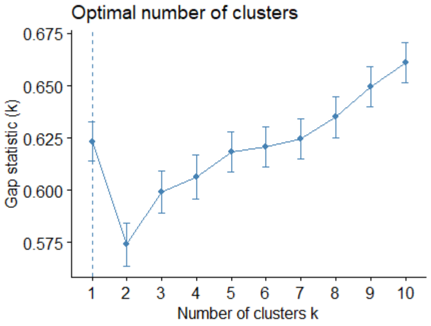

Note. $N_{\text{Study 1}}$ = 489, $N_{\text{Study 2}}$ = 618, $N_{\text{Study 3, Germany}}$= 420, $N_{\text{Study 3, UK}}$ = 440.
